# Mixed triplet-singlet order parameter in decoupled superconducting 1H monolayers of transition-metal dichalcogenides

**DOI:** 10.1038/s41467-026-75677-3

**Published:** 2026-07-16

**Authors:** Avior Almoalem, Sajilesh K. P., Roni Anna Gofman, Yuval Nitzav, Ilay Mangel, Nitzan Ragoler, Jun Fujii, Ivana Vobornik, Francois Bertran, Amit Kanigel, Jonathan Ruhman, Vidya Madhavan

**Affiliations:** 1 Department of Physics and Materials Research Laboratory, Grainger College of Engineering, University of Illinois at Urbana-Champaign, Urbana, IL USA; 2 Department of Physics, Technion, Haifa, 3200003 Israel; 3 Istituto Officina dei Materiali (IOM)-CNR, Area Science Park, S.S.14, Km 163.5, 34149 Trieste, Italy; 4 SOLEIL Synchrotron, ĽOrme des Merisiers, D’epartementale 128, 91190 Saint-Aubin, France; 5 Department of Physics, Bar-Ilan University, 52900 Ramat Gan, Israel

**Keywords:** Superconducting properties and materials, Two-dimensional materials, Superconducting properties and materials, Surfaces, interfaces and thin films, Electronic structure

## Abstract

Understanding the emergence of unconventional superconductivity, where the order parameter deviates from simple isotropic s-wave pairing, is a central challenge in condensed matter physics. Some transition-metal dichalcogenides (TMDCs), though generally regarded as conventional superconductors, display signatures of unconventional pairing and thus provide a particularly intriguing platform to explore how exotic states arise. Here we investigate the misfit compound (SnS)_1.15_(TaS_2_), a heterostructure composed of alternating SnS and 1H-TaS_2_ layers. Using transport, photoemission, and scanning tunneling spectroscopy, we demonstrate that the SnS layers effectively decouple the TaS_2_ into electronically isolated 1H sheets. In this limit, the tunneling density of states on the 1H layer reveals a clear two-gap superconducting spectrum with *T*_*c*_*≃*3.1 K. A theoretical model based on lack of inversion symmetry of the system and finite-range attraction reproduces the observed multi-gap structure as a mixed singlet-triplet state. These results establish misfit compounds as a powerful platform for studying unconventional superconductivity in isolated 1H layers and for realizing multiple uncoupled superconductors within a single crystal.

## Abstract (web summary)

The authors study single crystals of “misfit” compound (SnS)1.15(TaS2), a heterostructure composed of alternating SnS and 1H-TaS2 layers where SnS layers effectively decouple the TaS2 into electronically isolated 1H sheets. The spectroscopic data are consistent with a theoretical model of a mixed-singlet triplet superconducting state based on the non-centrosymmetric structure of the 1H sheets.

## Introduction

The origin of unconventional pairing states remains one of the most compelling open questions in superconductivity (SC). While most superconductors host simple, isotropic s-wave Cooper pairs, analogous to spherical atomic orbitals, many materials depart from this paradigm. In such systems, the superconducting order parameter can have reduced symmetry, sign changes, or nodes, enabling a rich variety of emergent phenomena. These include nodal quasiparticles with characteristic power-law thermodynamics, spin-triplet states^1–3^, spatially modulated pair-density waves^4–6^, and topological phases supporting Majorana excitations and chiral edge currents^7–9^. Prominent examples are the d-wave cuprates^10,11^, certain heavy-fermion compounds^12,13^, layered oxide superconductors^2,14,15^, and more recently, van der Waals (vdW) heterostructures^16–19^. Despite extensive experimental evidence, the microscopic ingredients driving such pairing remain elusive.

It is widely believed that strong, short-range Coulomb repulsion and the resulting magnetic fluctuations are crucial for these pairing states^11,20–22^. This suggests a connection between non-s-wave superconductivity and phenomena like strong correlations and quantum criticality^23–28^. However, not all candidate materials fit neatly into these categories, and even when they do, the precise mechanisms driving the pairing remain elusive.

A compelling platform for investigating these unconventional states is found in the transition metal dichalcogenide (TMDC) family^29–32^. These are van der Waals materials, essentially stacks of two-dimensional crystals, which can exist in various polymorphs. The 2H polymorphs, such as 2H-NbSe_2_, 2H-TaS_2_, and 2H-TaSe_2_, are superconductors with a *T*_*C*_ ranging between 0.7 and 7 K^31,33–35^. While they are often described by BCS theory, they also exhibit a low-temperature charge density wave (CDW) transition, or at least a significant softening of their phonon spectra towards such an order^36–38^. The basic 1H layers possess a non-centrosymmetric structure and strong spin-orbit coupling, which results in strong spin-valley locking (SVL)^34,39–42^.

In stark contrast, the 1T polymorphs of the same compounds undergo a much stronger CDW transition, leading to significant band reconstruction. For example, 1T-TaS_2_ becomes insulating at low temperatures and is a candidate quantum spin liquid^43–45^. These two behaviors converge in the 4Hb polymorph of TaS_2_, a unique hybrid structure composed of an alternating stack of 1H-TaS_2_ and 1T-TaS_2_ layers^18,46^. This unique stacking is believed to be the origin of the host of unconventional phenomena^47,48^, including multi-component and topological superconductivity^49–53^, absent in its individual constituent layer.

Recent measurements on exfoliated H-TaS_2_ flakes have further complicated this picture by showing evidence for a multi-gap superconducting state in the monolayer limit, which disappears for two layers or more^35^. In contrast, a monolayer grown by molecular beam epitaxy (MBE) has signs of a nodal order parameter, suggested to be f-wave^54^. This leads to a crucial overarching question: what drives the emergence of an unconventional order parameter in these materials, and is the exotic superconducting state intrinsic to the 1H layer, or is it strongly influenced by its environment?

To answer this question and differentiate between an isolated monolayer and a system involving strongly correlated layers, we performed a low-temperature study using bulk and local probes of the superconducting phase in the misfit material (SnS)_1.15_(TaS_2_). Misfit compounds, such as (SnS)_1.15_(TaS_2_), (SnSe)_1.16_(NbSe_2_), and (PbS)_1.13_(TaS_2_), are heterostructures of TMDC’s and semi-conducting mono-chalcogen layers, with a mismatch between the lattice vectors of the different layers. This mismatch effectively decouples the layers into true two-dimensional systems^55^.

In our specific case, the decoupling of the 1H layers not only create an electronic structure with reduced dimensionality but also provides us with a non-centrosymmetric superconductor. The lack of global inversion symmetry permits the mixing of gaps, otherwise distinct by symmetry, giving rise to a superconducting gap more complicated than the mundane singlet s-wave, with a complex spin configuration. This new paved way of achieving superconductivity based on pairing-channels with higher angular momentum, therefore, differentiates the system from other centrosymmetric TMDC’s superconductors, such as 4Hb-TaS_2_ or NbSe_2_. Although misfit materials have been known for a long time, recent interest in superconductivity in reduced-dimensional systems has renewed attention to these compounds, establishing them as a versatile platform for exploring two-dimensional superconductivity, where lattice incommensurability and vanishing inter-layer coupling can fundamentally reshape electronic and superconducting properties^56,57^. In such systems, superconductivity often emerges as an intrinsically two-dimensional state with enhanced anisotropy and Ising characteristics, strongly influenced by electronic correlation effects^55,58–60^.

This two-dimensional state was shown and explored previously using transport, STM and ARPES measurements in previous studies of misfit compounds^55,60–62^. Yet, these studies did not address the question of the nature of the superconducting order parameter in the TMD layer, and its structure in k-space. Moreover, in all previous studies, the mono-chalcogen layer (SnS, PbS, and SnSe) was assumed to be insulating.

Interestingly, the present study of (SnS)_1.15_(TaS_2_) reveals that the low-energy density of states (DOS) in the superconducting phase of the 1H layers is characterized by two distinct superconducting energy scales, i.e., a two-gap structure, which is absent in bulk 2H-TaS_2_. Moreover, we also observe the emergence of superconductivity within the SnS layer itself, which appears to be intrinsic to that layer rather than induced by proximity from the 1H-TaS_2_ layers. By considering our tunneling data on the 1H layer, together with data on exfoliated layers of 1H-TaS_2_^35^, we argue that the key factor underlying this difference is symmetry. Specifically, both the misfit compound and the monolayer lack inversion symmetry, which leads to strong singlet–triplet mixing.

This mixing of gaps with different symmetries induces a gap asymmetry between the inner and outer Fermi surfaces surrounding **K** (and **K’**). In contrast, when a monolayer is coupled to an inversion partner through inter-layer tunneling, as in the 2H-TaS_2_ structure, the singlet–triplet mixing is suppressed, and the multi-gap feature disappears. The 1H-monolayer notion in the misfit compound is also evident by our ARPES results, which indicate vanishing inter-layer coupling between the 1H layers, and the observed superconducting transition temperature, *T*_*c*_ = 3.1 K, which is close to the transition temperature measured in exfoliated monolayer studies^34–36^. This suggests that the SnS layer acts as an effective buffer between adjacent 1H layers, rendering the material equivalent to a stack of isolated 1H-TaS_2_ monolayers.

## Multi-gap spectrum in 1H monolayer

**Fig. 1:**
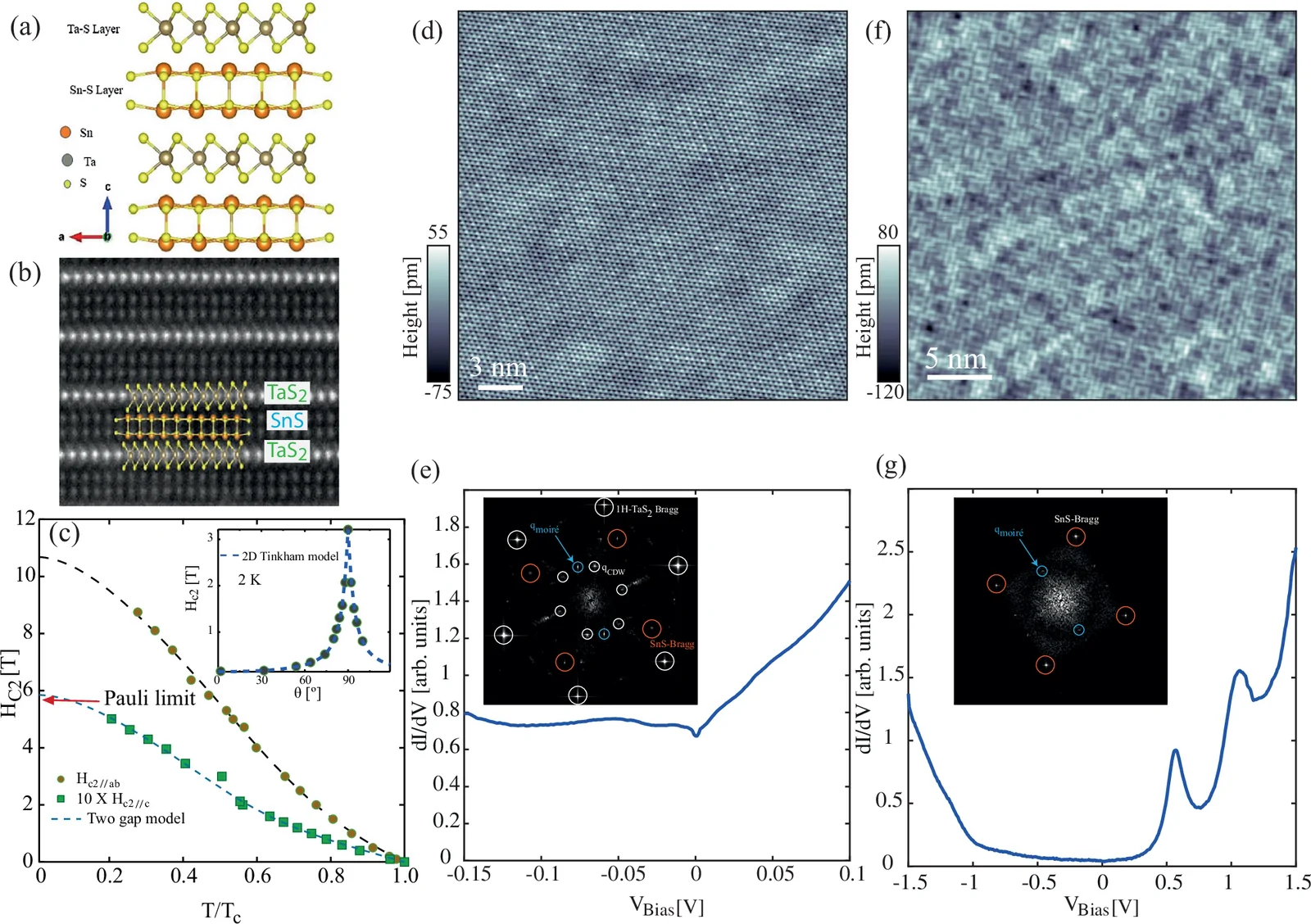
Structure of the different layers in (SnS)_1.15_(TaS_2_) single crystal. **a** Crystal structure of (SnS)_1.15_(TaS_2_) viewed along the *b* axis. **b** High-resolution TEM image showing the alternating stacking of TaS_2_ and SnS layers. **c** Temperature dependence of upper-critical field for out-of-plane (green squares) and in-plane directions (brown circles). The dotted line shows the fitting using the two-gap model. The inset shows the angular dependence of the upper-critical field fitted using the 2D-Tinkham model^65^. **d** Topography of 1H-TaS_2_ layer in (SnS)_1.15_(TaS_2_) (*I*_set_ = 250 pA and *V*_Bias_ = *−*30 mV). **e** Spectra taken at the same location as (**d**). The spectrum is reminiscent of that of the 1H-TaS_2_ layer in 4Hb-TaS_2_. The small dip at the Fermi level is reminiscent of the SC gap. The modulation amplitude and step size smear the gap, leaving only a small dip. (*I*_set_ = 300 pA, *V*_Bias_ = *−*150 mV, *V*_mod_ = 0.5 mV). Inset: FFT of the topography shown in (**d**); 1H-TaS_2_ Bragg and CDW peaks marked by white circles, SnS Bragg peaks in red circles, and the moiré peak marked with a cyan arrow. **f**, **g** Same for the SnS layer. The lack of a clear square lattice in the topography is probably due to damage to the layer in the cleaving process, where some atoms remain on the opposing cleaved surface. For **f** *I*_set_ = 250 pA and *V*_Bias_ = *−*1.5 V; for **g** *I*_set_ = 150 pA, *V*_Bias_ = *−*1.5 V, *V*_mod_ = 6 mV.

The compound (SnS)_1.15_TaS_2_ consists of alternating layers of SnS and 1H layers, as shown in Fig. 1a. Bulk SnS is a semiconductor with an indirect band gap of 1.3 eV^63^, and 1H-TaS_2_ is a metal that undergoes consecutive CDW and SC transitions. The SnS layers adopt a distorted NaCl structure with a double layer of tin and sulfur, while the 1H layers crystallize in a non-centrosymmetric structure, where Ta atoms are trigonal prismatically coordinated by six sulfur atoms.

Unlike in 4Hb-TaS_2_, where trigonal prismatic Ta-S layers are rotated by 180^◦^ relative to each other, here the 1H layers maintain the same orientation, making the crystal lattice lack overall inversion symmetry. Furthermore, the 1H-TaS_2_ layers are shifted by half a unit cell along the *b*-axis. Due to the different symmetries of the two lattices, an irrational ratio exists between the lattice constants along one of the in-plane directions while maintaining a perfect stacking order along the *c*-axis, thus creating a misfit structure.

The samples’ high quality is evident in the peak sharpness in X-ray diffraction measurements. The existence of the (00 *l*) reflections confirms that the *c*-axis is perpendicular to the sample surface, shown in Supplementary Fig. 1. High-resolution transmission electron microscopy (HRTEM) results confirm the crystal structure of alternate stacking of 1H and SnS layers, as shown in Fig. 1b. The two-dimensional behavior of the superconducting state in the sample is suggested by **H**_C2_ anisotropy, inset of Fig. 1c.

Upon cleaving, the exposed surface includes areas of both SnS and 1H-TaS_2_ terminations. Figure 1d, e show the topography and high-energy tunneling spectra of the 1H termination, respectively. Bragg, CDW, and moiré peaks are marked in the FFT shown in the inset of Fig. 1e. High energy spectra are similar to previously measured 1H layer in the heterostructure 4Hb-TaS_2_^49^. The CDW **Q** vector is similar to other studies with ${{{{\bf{Q}}}}}_{{CDW}}=0.3543\cdot \,{{{{\bf{Q}}}}}_{{Bragg}}$. Figure 1f, g similarly show the topography and spectra of the SnS layer. Bragg peaks of the square lattice, with the same moiré peak, are marked in the FFT. The moiré peaks in both layers originate from the mismatch and rotation of the two basic unit cells. The moiré peaks were also observed in a high-temperature STM study of the same material^64^.

In the SnS layer, the d*I/*d*V* high-energy spectrum is reminiscent of an insulator, as expected from the semiconducting SnS. The semiconducting gap, which exists from −0.5 eV up to 0.5 eV, is not full and has a finite DOS at the Fermi level. The residual DOS is distributed across the SnS termination, indicating that it is an intrinsic property of the layer.

Electrical transport measurements were performed on single crystals in a four-probe configuration, and the resistivity as a function of temperature is shown in Supplementary Fig. 2. The monotonic decrease in resistivity emphasizes the metallic nature of the material. Interestingly, no sign of the CDW transition appears in resistivity measurements, although CDW peaks are clearly visible in the FFT of the 1H topography, emphasizing the role of the SnS layer in transport and the need of a local probe differentiating between the two layers. The high residual resistivity ratio (RRR), ${\rho }_{300}/{\rho }_{10}$ = 12, is comparable to that of other misfit compounds and serves as another indicator of the high quality of the crystals. At low temperatures, a sharp drop in resistivity occurs around *T* = 3.05 K, indicating the SC transition (inset). This marks a considerable increase in *T*_*c*_ compared to pristine 2H-TaS_2_ (*T*_*c*_ = 0.8 K), bringing it closer to the value observed for monolayer TaS_2_ (*T*_*c*_ = 3.4 K)^36^.

We define the critical field as the field at which resistivity drops to 50% of its normal state value. Temperature dependence of both in- and out-of-plane critical fields is shown in Fig. 1c, with the raw data shown in Supplementary Fig. 3. The inset shows the angular dependence of the critical field at 2 K, with the data fitted to the 2D Tinkham model^65^. We find an anisotropy ratio of 19 between the in-plane and out-of-plane critical fields.

Remarkably, we find an increasing concave temperature dependence of **H**_c2_ near *T*_c_ for both field orientations, instead of the conventional Werthamer–Helfand–Hohenberg (WHH) behavior. This behavior is most commonly attributed to the presence of two superconducting gaps, as observed in MgB_2_^66^, YNi_2_B_2_C^67^, LuNi_2_B_2_C^68^, and 2H-NbSe_2_^69^, although alternative explanations have also been proposed^70–73^. A two-gap model, described in the Supplementary Information, is used to fit the data. The fit results are shown as dashed lines in Fig. 1c. We find that the in-plane critical field surpasses the Pauli limit by a factor of 1.95, an indicator of Ising superconductivity^74^.

Ising SC is a result of spin-valley locking, a phenomenon where the electron’s spin and valley degrees of freedom are intrinsically coupled due to strong spin-orbit interaction and broken inversion symmetry. This coupling ensures that the spin orientation is uniquely tied to the valley index, resulting in opposite spin polarizations at different valleys (e.g., **K** and **K’** points in the Brillouin zone). Cooper pairs forming the Ising SC inherit the spin-valley locking and can withstand large in-plane magnetic fields.

**Fig. 2:**
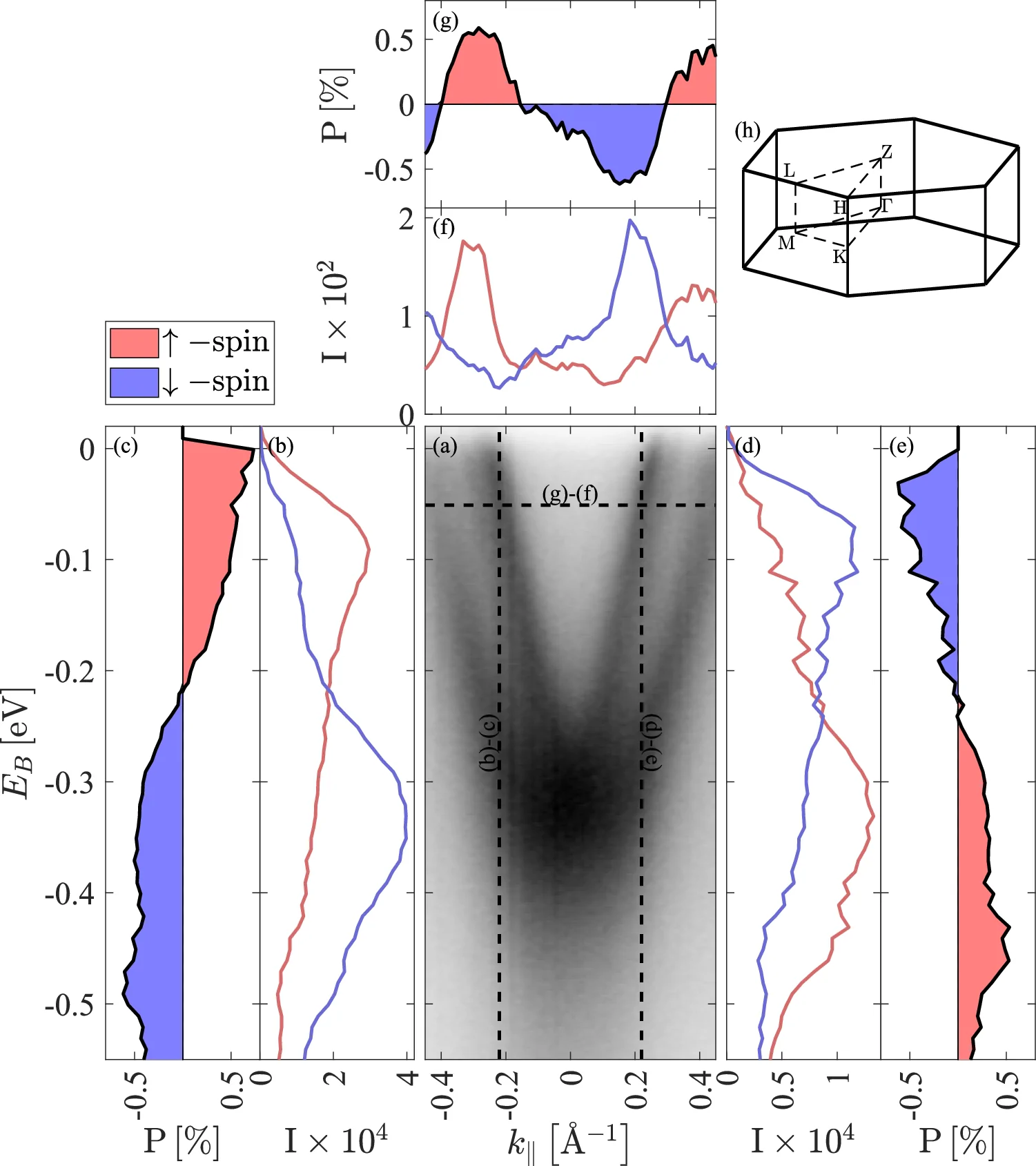
Spin polarized ARPES data acquired on 1H layer in (SnS)_1.15_(TaS_2_) single crystal. **a** ARPES dispersion measured at 56 eV, corresponding to the true M point (i.e., *k*_*z*_ ≃  0). Dashed black lines indicate the momentum- and energy-distribution cuts used for the spin-resolved measurements, whose results are shown in panels (**b**–**g**). Spin-resolved EDCs acquired at 25 eV for the left vertical cut: **b** spin-up (red) and spin-down (blue) intensities, and **c** the corresponding spin polarization (black), with positive/negative polarization indicated by red/blue shading. **d**, **e** Same as (**b**, **c**), but for the right vertical cut. Spin-resolved MDCs acquired at 25 eV along the horizontal dashed line: **f** spin-up (red) and spin-down (blue) intensities, and **g** the corresponding spin polarization (black). **h** Hexagonal Brillouin zone of 1H-TaS_2_ with high-symmetry points labeled. In **b**–**g**, red and blue curves represent spin-up and spin-down intensities along the out-of-plane direction, respectively.

We show spin valley locking in (SnS)_1.15_(TaS_2_) directly, by using spin-resolved angle resolved photoemission spectroscopy (ARPES) measurements, shown in Fig. 2. Spin-polarized data measure the projection of the spin in the out-of-plane direction for each state in the momentum space. Figure 2a shows ARPES spectra along the in-plane projected **K-M-K’** momentum direction (see Brillouin zone in Fig. 2h), with clear polarization of the bands seen in both the energy distribution curves (EDCs) (panels b–e) and the momentum distribution curve (MDC) (panels f, g). The MDC is taken 50 meV below the Fermi level (panels f, g), thus giving the polarization for the occupied portion of the band structure close to the Fermi level. This is consistent with previous measured TMDCs with a 2H crystal structure^39–42^.

We further show that the band structure at the **M** point of the Brillouin zone exhibits deeper electron pockets and the absence of a gap between the inner and outer pockets around *−*0.325 eV, in contrast to bulk 2H-TaS_2_^42^. These observations have two immediate conclusions: (i) a vanishing inter-layer coupling of the 1H bands and (ii) a charge-transfer from the SnS layer to the 1H-TaS_2_ layer. The former is a natural consequence predicted by tight-binding calculations and observed in previous studies (see Supplementary Fig. 4)^39,41,42^, while the latter arises from the deeper band around the **M** point^42^. The vanishing inter-layer coupling is further shown by comparing the EDC at the **L** point, where the gap is closed from symmetry considerations^41,42^, and the **M** point, where the gap is the largest for a given coupling. The two EDC are identical for the misfit material, as opposed to pristine 2H-TaS_2_, thus reinforcing the conclusion that the inter-layer coupling is smaller than our ARPES resolution of 10 meV, Supplementary Figs. 4 and 5.

**Fig. 3:**
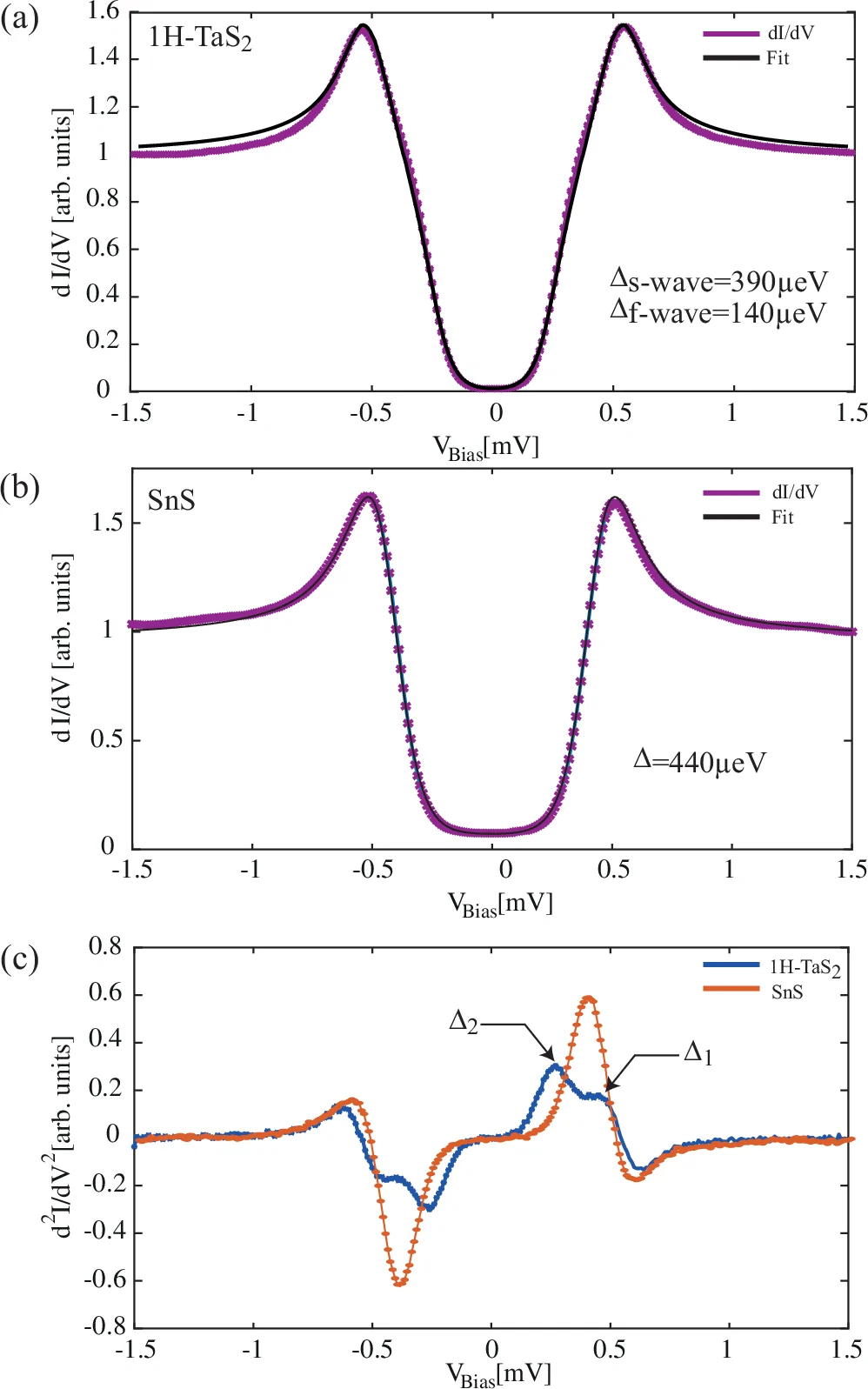
Superconducting spectra acquired on 1H and SnS layers in (SnS)_1.15_(TaS_2_). **a** The superconducting gap in 1H-TaS_2_ layer. dI/dV spectrum obtained on the 1H-TaS_2_ layer shown in Fig. 1d. The superconducting gap has no residual DOS, unlike the case of 4Hb-TaS_2_. Overlying the data is a fit to a two-gap function with different sizes and symmetries. The clear change in the slope of the spectra around 300 µeV emphasize the anisotropic behavior (*I*_*set*_ = 50 pA and *V*_*Bias*_ = −1.5 meV, *T* = 0.3 K). The two gaps used are s-wave and f-wave order parameters as used in the theoretical model. **b** The superconducting gap in the SnS layer. d*I*/d*V* spectrum obtained on the SnS layer shown in Fig. 1f. The SC gap is well fitted using a single BCS gap and a residual DOS (*I*_*set*_ = 50 pA and *V*_*Bias*_ = 2 meV, *T* = 0.3 K). **c** d^2^*I/*d*V* ^2^ taken from the data in (**a**, **b**). The two arrows mark the energies of the two gaps acquired on the 1H-TaS_2_ layer, matching the energies of the change in the slope of the d*I*/d*V*. In **a**, **b**, purple and black curves represents experimental acquired data and fits, respectively.

We now turn to low temperature, low energy STM/S data. We directly measure the superconducting gap at low temperatures with high resolution. The spectra obtained from the 1H layer reveal a distinct gap structure that resembles a two-gap spectrum of different magnitudes. Fitting the data to a two-gap order parameter of s-wave and f-wave symmetries yields an excellent match (see Supplementary Information for details), as shown in Fig. 3a, whereas a single s-wave gap provides a poor fit (see Supplementary Fig. 6). The spectrum remains uniform through the scan area (see Supplementary Fig. 7), with the average spectrum displayed in Fig. 3a. Pronounced coherence peaks are observed at an experimental value of 560 µeV, above and below the Fermi level, with the fit yielding 390 and 140 µeV for the large s-wave and smaller f-wave components. The slope of the spectra, representing the change in DOS and thus the disappearance of states in the gap, steepens around 300 µeV, therefore signaling the energy at which the gap is now full across all points on the Fermi surface. To highlight this feature, we present the first derivative of the spectra, which reveals two distinct “kinks” corresponding to the onset of the two gap features, as shown in Fig. 3c.

Alongside the 1H layer, we perform similar measurements on the SnS layer. Bulk SnS is semiconducting and therefore does not suppose to host superconductivity. Nevertheless, a clear gap can be observed in the spectrum acquired on the SnS layer, as shown in Fig. 3b. The magnitude of the gap in the SnS layer is slightly higher than the larger gap in the 1H layer, as given by a fit to a single s-wave model. Such a result raise two questions: whether this gap is of superconducting origin? And if so, whether it is of proximity origin? To answer this question, we first image vortices in the SnS layer (Supplementary Fig. 8), which stand in contrast to 4Hb-TaS_2_, where the non-SC 1T layer shows no vortices, although a vortex exists below the layer as measured on a step edge in an STM study^49^. This confirms the gap to be of superconducting origin. Next, we confirm that the gap on the SnS layer is of intrinsic superconducting origin by applying magnetic field and study the gap behavior (Supplementary Fig. 9). The magnetic field dependence of the gaps on the two layers shows that the gap in the SnS layer is deeper under applied field of **B** = 0.1 T, which is unlikely to occur if the gap in the SnS layer was of proximity origin. These observations confirm the SnS layer to be independent superconducting layer.

Imaging the vortex in the SnS layer also allows us to extract the coherence length by fitting the Fermi-level DOS to $N({E}_{F},\,x)={N}_{0}{{{{\rm{e}}}}}^{-\frac{x}{\xi }}+{N}_{\infty }$^49^, yielding ${\xi }_{{vortex}}$ ≃ 35 ± 5 nm, where *x* is the distance from the core and *N*_0_ and *N*_∞_ denote the DOS at the vortex core and far from the vortex, respectively. This value is consistent with the coherence length estimated from the upper critical field, ${{{{\bf{H}}}}}_{C2}={\varPhi }_{0}/(2\pi {\xi }^{2})$, which gives ${\xi }_{{{{{\bf{H}}}}}_{C2}}$ ≃ 30 nm at *T* = 400 mK and similar to other studies^75^. The agreement between the coherence length extracted from the vortex size and that inferred from *H*_*c*2_ highlights the importance of directly measuring the superconducting gap in the SnS layer. The influence of the SnS superconducting gap on thermodynamic quantities obtained from bulk-averaged measurements is substantial. We therefore attribute the two-gap behavior observed in the **H**_*c*2_ transport data to the coexistence of superconducting gaps in the 1H-TaS_2_ layer and in the SnS layer.

The bulk transport measurements and the tunneling spectrum in the 1H and SnS layers are consistent with a two-gap scenario of the overall system and cannot be explained within a single-gap framework. Furthermore, unlike the s-wave gap seen in the SnS layer, local spectra obtained on the 1H layer shows that the order parameter in that layer is by itself not consistent with a single gap and deserve more attention. Considering that STM results show a multi-gap feature in 1H-TaS_2_ along with two-dimensional electronic structure revealed by ARPES, a theoretical model, grounded in simple symmetry considerations and realistic band structure parameters, of a multi-gap structure naturally follows.

## Theory of spin triplet-singlet mixing

Comparison of our tunneling density of states with monolayer 1H-TaS_2_ flakes^35^, where sub-gap features were observed, suggests a correlation between inter-layer coupling and the prominence of multi-gap features. Specifically, multi-gap behavior appears in SnS-TaS_2_, monolayer 1H-TaS_2_, and arguably in 4Hb-TaS_2_^76,77^; systems in which inter-layer coupling is effectively quenched, for example via increased inter-layer separation. In contrast, the multi-gap spectral signature disappears in 2H-TaS_2_. The importance of inter-layer coupling effects in bulk 2H-TaS_2_, as opposed to the other TaS_2_-based heterostructures, was demonstrated by spin-ARPES measurements^42^. This raises two key questions: why does this material host potential multi-gap superconductivity, and why is it suppressed by inter- layer coupling?

**Fig. 4:**
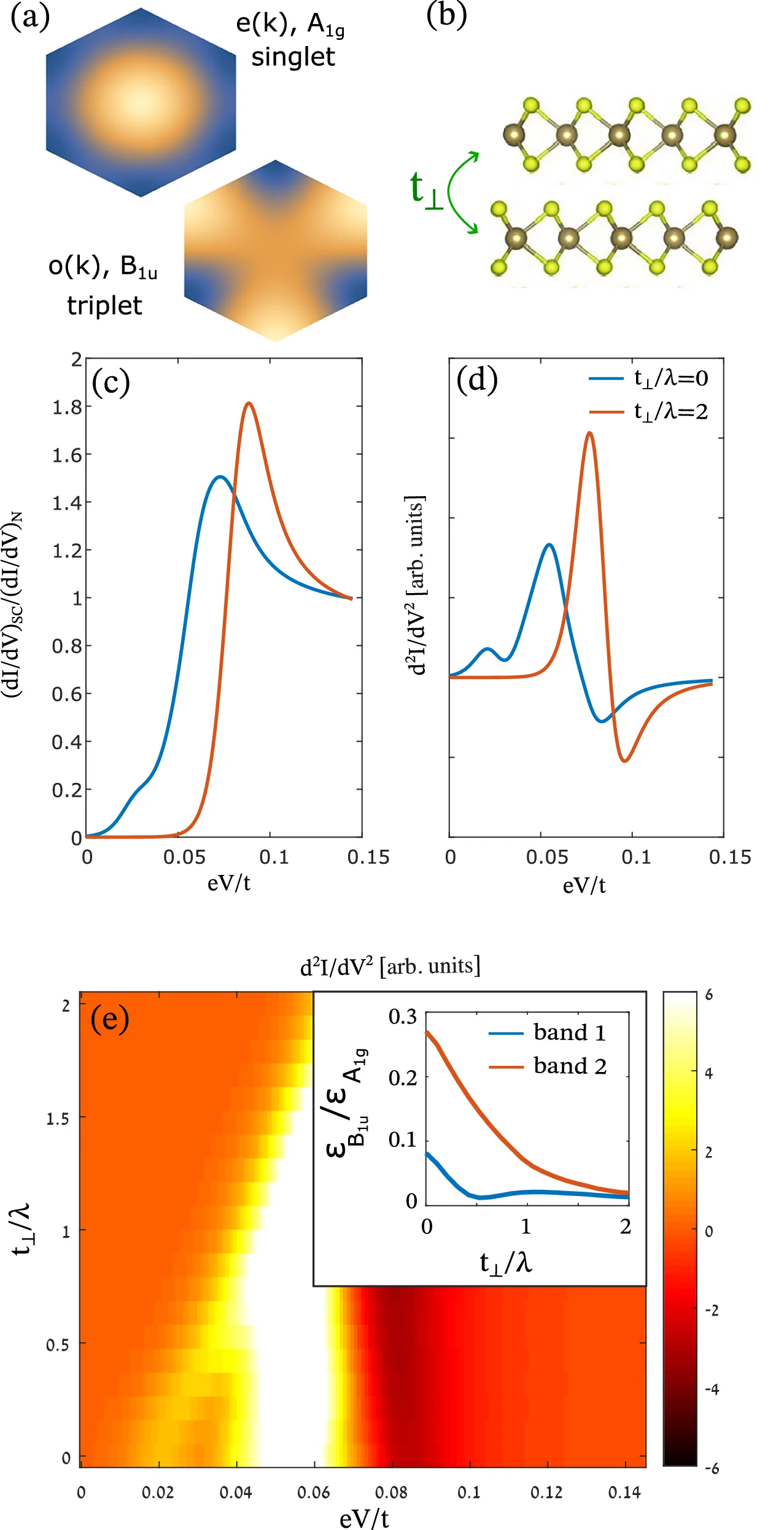
Theoretical model of mixed s and f gaps in the broken inversion 1H layer. **a** Schematic of the theoretical model. We consider 2H-TaS_2_ with a tunable inter-layer hopping *t*_⊥_ and Ising spin–orbit coupling *λ*. The attractive interactions in each layer are assumed to be finite range (on-site, nearest-neighbor, and next-nearest-neighbor). **b** Cartoon of the model consisting of two 1H layers, with inter-layer coupling *t*_⊥_. **c** The resulting tunneling density of states (DOS) for *t*_⊥_ = 0 (blue) and *t*_⊥_*/λ* = 2 as a function of *eV/t*, the bias voltage in units of the hopping parameter (see Supplementary Information). **d** Derivative of the DOS shown in (**c**), revealing two peaks corresponding to two distinct gaps. **e** Color map of the derivative of the DOS for various values of the ratio *t*_⊥_*/λ*. The smaller gap continuously grows and eventually merges with the main gap around *t*_⊥_ ≃ 0.8 *λ*. The inset shows the ratio between the triplet and singlet components of the gap functions, *ε*_*B*1*u*_*/ε*_*A*1*g*_, as a function of *t*_⊥_*/λ* for the two bands of 2H-TaS_2_, indicating that mixing with the *f* -wave order parameter is responsible for the two-gap feature.

A natural scenario that accounts for these observations is singlet–triplet mixing due to local inversion-symmetry breaking^78^. To explore this idea, it is helpful to consider irreducible representations (irreps) of the higher point-group symmetry ${D}_{6h}$. In particular, we consider two one-dimensional irreps: ${A}_{1g}$, which includes the constant function 1 and the even function $e\left({{{\bf{k}}}}\right)$, and ${B}_{1u}$, which includes the odd function o(**k**). The latter can be viewed as a lattice version of an “*f*-wave,” while the former correspond to “*s*-wave” and “extended *s*-wave” components, respectively (see Fig. 4a and ref. ^78^ for more details). Both irreps are symmetric under ${C}_{3}$ rotations and mirror-*x* but differ in their parity: 1 and $e\left({{{\bf{k}}}}\right)$ are even under inversion, while o(**k**) is odd. Thus, all three basis function mix in the isolated the *H*-layer given that the inversion symmetry is locally broken.

The superconducting order parameter in a two-dimensional trigonal crystal can be decomposed into contributions from these irreps via

1 $${\hat{\Delta }}^{\sigma {\sigma }^{{\prime} }}\left({{{\bf{k}}}}\right)={\sum }_{\Gamma }{\varepsilon }_{\Gamma }{f}_{\Gamma }\left({{{\bf{k}}}}\right){\chi }_{\sigma {\sigma }^{{\prime} }}^{\Gamma },$$

where ${f}_{\varGamma }$(**k**) denotes the orbital form factor, ${\chi }^{\varGamma }$ is the corresponding spin matrix, and ${\varepsilon }_{\varGamma }$ are the amplitudes. For example, the even-parity (*s*-wave) basis function ${f}_{{A}_{1g}}$ (**k**) (a superposition of 1 and *e*(**k**)) is associated with the singlet matrix ${\chi }^{{A}_{1g}}$ = $-i{\sigma }^{y}$, while the odd-parity (*f*-wave) function ${f}_{{B}_{1u}}\,$= o(**k**) is paired with the triplet matrix ${\chi }^{{B}_{1u}}=\,-i{\sigma }^{y}{\sigma }^{z}$^79^.

In a 1H monolayer, inversion symmetry is absent, removing the symmetry distinction between the ${A}_{1g}$ and ${B}_{1u}$ channels and allowing them to mix. This situation is analogous to the Stark effect, where an electric field along **z** mixes atomic *s* and ${p}_{z}$ orbitals, producing split superpositions. As a result, the superconducting order parameter in 1H-TaS_2_ generically contains both singlet and triplet components. The relative weights of these components, ${\varepsilon }_{{A}_{1g}}$ and ${\varepsilon }_{{B}_{1u}}$, are controlled by material parameters, such as spin–orbit coupling and the pairing interaction.

For the specific Fermi-surface geometry of 1H-TaS_2_, this mixed-parity order parameter can manifest as a multi-gap structure, as observed in Fig. 3a. The key point is that, due to Ising spin–orbit coupling, the Fermi surfaces near **K** and **K’** are split into helicity bands with opposite out-of-plane spin polarization. Upon projection onto these helicity bands, the ${A}_{1g}-{B}_{1u}$ superposition yields gap amplitudes ${\varepsilon }_{{A}_{1g}}\,{f}_{{A}_{1g}}\,({{{\boldsymbol{k}}}})\,\pm \,{\varepsilon }_{{B}_{1u}}{f}_{{B}_{1u}}\,({{{\boldsymbol{k}}}})$. Consequently, the two SOC-split Fermi-surface sheets acquire different superconducting gap magnitudes. Time-reversal symmetry relates the helicity sectors at **K** and **K’**, so that the splitting is helicity-dependent and appears as a valley-resolved multi-gap structure. Therefore, when the weights ${\varepsilon }_{{A}_{1g}}$ and ${\varepsilon }_{{B}_{1u}}$ are comparable, the difference between the gaps can be significant, and a multi-gap feature in the density of states is expected. This behavior differs from a superposition within the *A*_1*g*_ irrep alone, which, by symmetry, produces gap anisotropy between the *Γ* and **K** Fermi surfaces. Nonetheless, although both scenarios yield similar multi-gap signatures in the tunneling density of states, they arise from fundamentally different symmetry mechanisms, motivating the need for experimental probes that can distinguish between them.

A natural way to distinguish between these scenarios is to tune the coupling between layers in an inversion-symmetric manner, as in 2H-TaS_2_. In the limit of weak inter-layer coupling, it is still reasonable to describe the order parameter as a superposition of monolayer states with singlet–triplet mixing^80^. In this way, one can construct gap functions that are eigenmodes of inversion but that locally, on each layer, still exhibit singlet–triplet mixing. As the inter-layer coupling increases, the system becomes increasingly sensitive to global inversion symmetry, suppressing local inversion-breaking effects and consequently reducing their admixture. In contrast, if the origin of the multi-gap feature in the density of states is not related to inversion-symmetry breaking, no such sensitivity to inter-layer coupling is expected.

To test this hypothesis, we construct a minimal single-orbital tight-binding model^81^ for the band structure of two H-TaS_2_ layers. Because our goal is to demonstrate how inter-layer coupling can diminish singlet–triplet mixing, we deliberately construct the model to mimic the inversion-symmetric structure of 2H-TaS_2_, rather than the situation realized in STS. The model incorporates three crucial ingredients: (i) a tunable inter-layer coupling *t*_⊥_ (see Fig. 4b); (ii) an Ising spin–orbit coupling *λ*; and (iii) a finite-range pairing interaction, including on-site, nearest-neighbor, and next-nearest-neighbor attraction. We suppress the on-site attraction relative to the longer-range terms due to screened Coulomb repulsion. All three ingredients are essential to explain the transition from multi-gap to single-gap superconductivity.

In Fig. 4c, we plot the resulting tunneling DOS for *t*_⊥_ = 0 (blue) and *t*_⊥_*/λ* = 2 as a function of *eV/t*, the bias voltage in units of the hopping parameter (see Supplementary Information). A clear two-gap feature emerges in the absence of inter-layer coupling and disappears for large *t*_⊥_*/λ*. In panel (d), we plot the derivative of the DOS shown in panel (c), revealing two peaks corresponding to two gaps. In panel (e), we show a color map of the derivative of the DOS for various values of the ratio *t*_⊥_*/λ*. The smaller gap continuously grows and eventually merges with the main gap around *t*_⊥_ ≃ 0.8 *λ*. The inset shows the ratio between the triplet and singlet components, ${\varepsilon }_{{B}_{1u}}/{\varepsilon }_{{A}_{1g}}$, as a function of *t*_⊥_*/λ*. The two traces correspond to the two spin-degenerate bands of a single 1H layer. The strong correlation between singlet–triplet mixing and the appearance of two gaps demonstrates that the theoretical model reproduces the main experimental observation: the multi-gap feature emerges in the limit where inter-layer tunneling is suppressed or where the inversion symmetry is broken, indicating that strong singlet–triplet mixing is its microscopic origin.

## Discussion

The misfit compound (SnS)_1.15_(TaS_2_) provides a natural realization of electronically decoupled 1H-TaS_2_ layers. The absence of **k**_*z*_ dispersion (Supplementary Fig. 4), the closing of the gap in the M point and the existence of spin-valley locking in ARPES data^39,42^ and strong transport anisotropy, confirm that inter-layer hybridization is strongly suppressed. The electronic structure, which emerges is characteristic of isolated 1H layers. In this regime, tunneling spectroscopy of the 1H layers reveals a multi-gap density of states.

This behavior can be placed in context with other TMDC systems. In bulk 2H-TaS_2_, STM measurements report a single gap of about 0.28 meV^35,82^. In MBE-grown monolayer 1H-TaS_2_, a single V-shaped gap has been reported^54^, suggesting a nodal gap structure, although with a low *T*_*c*_ around 1 K. Lower *T*_*C*_ suggests that other mechanisms might affect the observed superconducting characteristics, such as disorder. Contrary to the MBE grown monolayer, we do not observe the formation of a single component nodal order-parameter, exclusively in ultra-clean samples, rather a two-gap structure uniformly at all locations. This may point to the influence that the substrate has on the sample quality and the observed signatures. As an example, strain from the HOPG substrate, due to mismatch of the lattice parameters, might influence the electron-phonon coupling or the phonons themselves to create different order parameters^83^. Moreover, different interactions (on- site, nearest-neighbor, etc.), due to different ion distances, can lead to different admixtures of the order parameters. In contrast, exfoliated monolayers with *T*_*c*_ near 3.5 K display two distinct gaps of approximately 0.25 and 0.46 meV^35^, in close agreement with our findings. We emphasize that all data were acquired in strain-free regions. Any strain, arising from the lattice mismatch between the SnS and 1H layers, appears to be relieved through the formation of large-scale line features observed in the topography (Supplementary Fig. 7). The measurements were performed in regions away from such lines (Supplementary Fig. 7), as well as adjacent to a step edge where no visible strain is detected (Supplementary Fig. 10).

Furthermore, the data were collected along line cuts extending over 30 and 50 nm, such as the data sets in Fig. 3 and Supplementary Fig. 10, respectively. The high degree of spectral uniformity along these cuts supports the conclusion that the observed spectra are intrinsic to the sample and not induced by strain.

Related behavior is also observed in 4Hb-TaS_2_, where the 1H layers are decoupled by intervening 1T layers^42^. Specific heat^18^ and STM measurements^49^ show a residual density of states below the superconducting gap, consistent with a second gap that is destroyed by pair-breaking impurities^76,77^. Thus, the observation of gapless sub-gap states in 4Hb-TaS_2_ can be seen as another example of two-gap superconductivity. The overall picture emerging from these comparisons is that multi-gap superconductivity is an intrinsic property of isolated 1H-TaS_2_ layers suppressed by inter-layer coupling.

To investigate the origin of this behavior we constructed a theoretical model based on a tight-binding Hamiltonian of a 2H bilayer with varying inter-layer coupling, Ising spin-orbit coupling, finite-range attraction, and weak on-site repulsion, naturally accounts for this phenomenology (Supplementary Figs. 11–13). The resulting superconducting order is a triplet-singlet mixture of *f*-wave and *s*-wave components, which depends on the inter-layer coupling (Supplementary Figs. 14–16). In the limit of strongly coupled layers, like the 2H system, the triplet component is small, and the gap is more or less uniform. In contrast, in the decoupled case, the *f*-wave component becomes comparable with the *s*-wave and leads to large variations in gap size across the Fermi pockets. This scenario agrees well with values obtained from the fit, yielding ${\triangle }_{{{\mathrm{1,2}}}}=s\pm f$, ${\triangle }_{1}$ = 530 µeV and ${\triangle }_{2}=$250 µeV corresponding to the coherence peaks and slope change energies. We also note that the resolution of our ARPES measurements constrains the inter-layer coupling to be below 10 meV (Supplementary Figs. 4 and 5). Given a spin-orbit coupling of approximately 120 meV^34,42^, our theoretical analysis indicates that even an inter-layer coupling as large as *t*_⊥_ = 10 meV leads to substantial mixing of singlet and triplet components, owing to the absence of inversion symmetry in the crystal.

The absence of inversion symmetry in 1H layers is central to this result, and the framework should be broadly applied to superconducting 1H TMDs, such as NbSe_2_. While bulk NbSe_2_ shows two gaps due to an additional Se-derived band, recent studies on monolayer NbSe_2_, and with S substitution, support a role for inversion-symmetry breaking in generating multi-component order parameter^31,84–88^. It is important to note that the model requires a substantial Coulomb repulsion such that the on-site attraction is reduced below the strength of the nearest- or next-nearest-neighbor attraction. This, in turn, implies that we are effectively considering a longer-range attractive interaction, which is plausible given the presence of soft phonon modes associated with the CDW transition.

We note that other mechanisms also have the potential to give rise to the two-gap signature in spectroscopic data. Elimination of the inter-layer coupling might affect the band structure in a way to modify the superconducting state. However, as ARPES data shows^42^, altering the inter-layer coupling produces only minor modifications to the band structure. For example, the M-point extremum is shifted by a few tens of meV over a bandwidth of 300 meV, while leaving the Fermi-level band structure essentially unchanged.

Other possible mechanisms are the McMillan model and anisotropic electron-phonon coupling. In the former case, a multi-band superconductor with intrinsic gap on each band can have quasi-particle coupling between the different bands. The coupling, essentially scattering of quasi-particles from one band to another due to impurities, results in spectroscopic signature similar to Fig. 3a^89^. This scenario becomes less plausible in clean samples, as our single crystals, shown in Fig. 1d. To emphasize this point, we compare spectroscopic data on and off a step edge. The presence of an edge increase electron scattering and should result in a stronger effect, yet this is absent in our measured spectroscopy (Supplementary Fig. 16). Finally, due to the absence of a two-gap spectroscopy in bulk 2H-TaS_2_^35^, it is unreasonable for this mechanism to have major effect exclusively in the 2D case.

For the latter scenario, an anisotropic electron-phonon coupling (EPC) originates from strong **K-K’** scattering^83^. Anisotropic EPC was proposed to explain the gap spectra in NbSe_2_, both in bulk and monolayer forms, and was measured experimentally using ARPES in both 2H-NbSe_2_ and 2H-TaS_2_^90,91^. Yet, unlike 2H-NbSe_2_, for 2H-TaS_2_, the DOS structure presented in this study formed only in the monolayer limit and does not exist in bulk 2H^35^, which points on a critical role of the inter-layer coupling. Scattering between **K** and **K’** in this model is independent of the inter-layer coupling, suggesting this mechanism is not responsible for our observed spectroscopy.

It should be noted that our study also indicates that the superconducting gap observed in the SnS layers is intrinsic and is not induced by proximity to the 1H-TaS_2_. The slightly larger magnitude of the SnS gap, the absence of a second proximity gap, the deeper SnS gap under applied magnetic field, and the high anisotropy of transport all argue against a proximity scenario. Moreover, theory predicts that a proximitized semiconductor-superconductor bilayer with a Fermi surface mismatch should exhibit a strongly reduced induced gap^92^, unlike what we observe.

Interestingly, bulk SnS becomes superconducting under high pressure^93,94^ above 40 GPa, with *T*_*c*_ up to 6 K, and a metallic phase that starts at pressures as low as 8 GPa. This result is consistent with theoretical expectations that finite DOS at the Fermi level can drive superconductivity when engineered using defects, electric-field effect or chemical substitution^93,94^. In the misfit compound, we propose that the cumulative effect of structural arrangement, which effectively applies an internal pressure to the SnS layers, together with the charge transfer between the layers, as appears in our ARPES data, give rise to finite DOS in the Fermi level, thus stabilizing an intrinsic superconducting phase even at ambient conditions.

In summary, (SnS)_1.15_(TaS_2_) emerges as a unique platform where decoupled 1H-TaS_2_ layers exhibit multigap superconductivity and where SnS layers host an independent superconducting state. This duality not only makes misfit compounds valuable for studying 1H physics in isolation but also opens opportunities to explore coupled and decoupled superconductors within a single crystalline system.

## Methods

### Material growth

Single crystals of (SnS)_1.15_(TaS_2_) were grown using the chemical vapor transport (CVT) method. The starting materials, weighed in their nominal stoichiometric ratios, were thoroughly mixed, pre-sintered, and sealed in an evacuated quartz ampoule along with SnCl_2_ as the transport agent. The sealed ampoule was then placed in a two-zone tube furnace with a temperature gradient of 100 °C, and the transport reaction was carried out for 2 weeks. Shiny, plate-like crystals formed in the cold end of the ampoule were collected and subsequently washed with ethanol to remove residual transport agent.

### STM

STM measurements were performed using a Unisoku STM at an instrument base temperature of 270 mK, using chemically etched and annealed tungsten tips. Spectra were acquired using a standard lock-in technique at a frequency of 907 Hz.

### ARPES

Spin-resolved ARPES data was acquired in the APE-LE beamline at Elettra, Italy. The light used at APE-LE is s polarized. Samples were cleaved in situ and measured at temperatures ranging from 10 to 30 K. The photon beamline entrance slit was set to 300 µm and the analyzer entrance slit to 400 µm.

To define the experimental geometry, the sample was first aligned in normal emission to locate the **Γ** point. The sample was then tilted so that the in-plane **K-M-K**^**′**^ high-symmetry direction lay along the analyzer slit. The final orientation was refined by acquiring Fermi-surface maps in deflector mode over a range of *−*15^◦^ to +15^◦^ in 0.75^◦^ steps (pass energy 20 eV, photon energies 20–20.97 eV in 10 meV steps, three frames per slice).

Spin-resolved MDCs were acquired at the final sample orientation with *Θ*_*x*_ = 0.8^◦^ and *Θ*_*y*_ scanned from *−*15^◦^ to +15^◦^ in steps of ∆*Θ*_*y*_ ≃ 0.39^◦^, at kinetic energies of 20.60 and 20.58 eV with 4 s integration per point. Spin-resolved EDCs were recorded at the two peak positions, with *Θ*_*x*_ = 0.8^◦^ fixed and *Θ*_*y*_ = 0.8^◦^ and 4.5^◦^, over an energy window of 20–20.97 eV in 10 meV steps and 4 s integration per point.

For the spin-resolved measurements, a VLEED detector was used. The polarization was calculated using a Sherman function of *S* = 0.27, with the band polarization given by refs. ^40–42^:

2 $$P=\frac{1}{S}\frac{{I}_{{pos}}-{I}_{{neg}}}{{I}_{{pos}}+{I}_{{neg}}}$$

where *I*_pos_ and *I*_neg_ are the measured intensities with the VLEED detector magnetized in the positive and negative directions, respectively. To average over temporal fluctuations in the photon flux and to remove systematic errors due to detector magnetization, we performed a measurement sequence with alternating field directions: positive, negative, negative, positive.

Accordingly, the spin-resolved intensities are obtained as

3 $${I}_{\uparrow }=\frac{1+P}{2}\left({I}_{{pos}}+{I}_{{neg}}\right)=\frac{{I}_{{pos}}}{2}\left(1+\frac{1}{S}\right)+\frac{{I}_{{neg}}}{2}\left(1-\frac{1}{S}\right)$$

4 $${I}_{\downarrow }=\frac{1-P}{2}\left({I}_{{pos}}+{I}_{{neg}}\right)=\frac{{I}_{{pos}}}{2}\left(1-\frac{1}{S}\right)+\frac{{I}_{{neg}}}{2}\left(1+\frac{1}{S}\right)$$

where ${I}_{\uparrow }$ and ${I}_{\downarrow }$ denote the spin-up and spin-down components, respectively. Note that if the Sherman function were ideal (*S* = 1), then ${I}_{\uparrow }$ would equal ${I}_{{pos}}$ and ${I}_{\downarrow }$ would equal ${I}_{{neg}}$, as the detector would perfectly distinguish spin.

Photon-energy dependent ARPES data was measured at the CASSIOPEE beamline at Soleil, France. To determine the ${k}_{z}$ dispersion from photon-energy-dependent ARPES, we use the free electron final state approximation for normal emission^40,42,95^:

5 $${k}_{z}=\sqrt{\left(2m/{\hslash }^{2}\left({E}_{{kin}}-{V}_{0}\right)\right)}$$

where ${V}_{0}\,$= 9 eV is the inner potential^42^ and ${E}_{{kin}}$ is the kinetic energy of the photo-emitted electron.

### Transport

Electrical transport measurements were carried out using a Quantum Design PPMS equipped with a dilution refrigerator, employing the standard four-probe technique. The sample was rotated relative to the applied magnetic field using an integrated rotator module for transport anisotropy measurements.

### Simulation of the expected DOS

The simulation is done using MATLAB as given in the Supplementary software file. The pseudo-code is as follows:

- Initiate grid and material parameters.
- Calculate band structure.
- Add interactions.
- Calculate the superconducting gap self-consistently.
- Extract the expected tunneling spectrum.

## Supplementary information

Supplementary Information

Transparent Peer Review File

## Data Availability

Due the complexity and volume generated in an ARPES and STM experiment, additional raw data is available from the corresponding author upon request. Processed data representing all the data in the published figures are openly available in the Zenodo repository under the following 10.5281/zenodo.20799749.

## Code availability

The code that supports the findings of this study is provided as a Supplementary software file. This code is to run on MATLAB.
