# Supplementary material for: Mixed triplet-singlet order parameter in decoupled superconducting 1H monolayers of transition-metal dichalcogenides
Source: Nat Commun. 2026 Jul 16;17:8718. doi: 10.1038/s41467-026-75677-3 (PMC13490457; doi:10.1038/s41467-026-75677-3)
Supplement: Supplementary file 1 [file 41467_2026_75677_MOESM1_ESM.pdf]

Supplementary Information for Mixed Triplet-Singlet Order Parameter in Decoupled Superconducting 1H Monolayer of Transition-Metal Dichalcogenides

Avior Almoalem,[1,*] Sajilesh K. P.,[2,*] Roni Anna Gofman,[2] Yuval Nitzav,[2] Ilay Mangel,[2] Nitzan Ragoler,[2] Jun Fujii,[3] Ivana Vobornik,[3] Francois Bertran,[4] Amit Kanigel,[2] Jonathan Ruhman,[5] and Vidya Madhavan[1]

[1]*Department of Physics and Materials Research Laboratory, Grainger College of Engineering, University of Illinois at Urbana-Champaign, Urbana, IL, USA*

[2]*Department of Physics, Technion, Haifa, 3200003, Israel*

[3]*Istituto Officina dei Materiali (IOM)-CNR, Area Science Park, S.S.14, Km 163.5, 34149 Trieste, Italy*

[4]*SOLEIL Synchrotron, L'Orme des Merisiers, Départementale 128, 91190 Saint-Aubin, France*

[5]*Department of Physics, Bar-Ilan University, 52900, Ramat Gan, Israel*

(Dated: July 1, 2026)

I. CRYSTALLOGRAPHY

X-ray diffraction pattern of the single crystal is shown in Supplementary Figure 1. The $(00l)$ reflections indicate the plane of the single crystal is perpendicular to the crystallographic c-axis and the sharpness of the peaks demonstrates the good quality of the single crystals. This data was taken using Rigaku, SmartLab 9kW high resolution system, employing Cu K_α ($\lambda = 1.54056$ Å) radiation. All of the reflection planes seen in the data are from the $(00l)$ family, indicating a high quality single crystal with one crystallographic orientation.

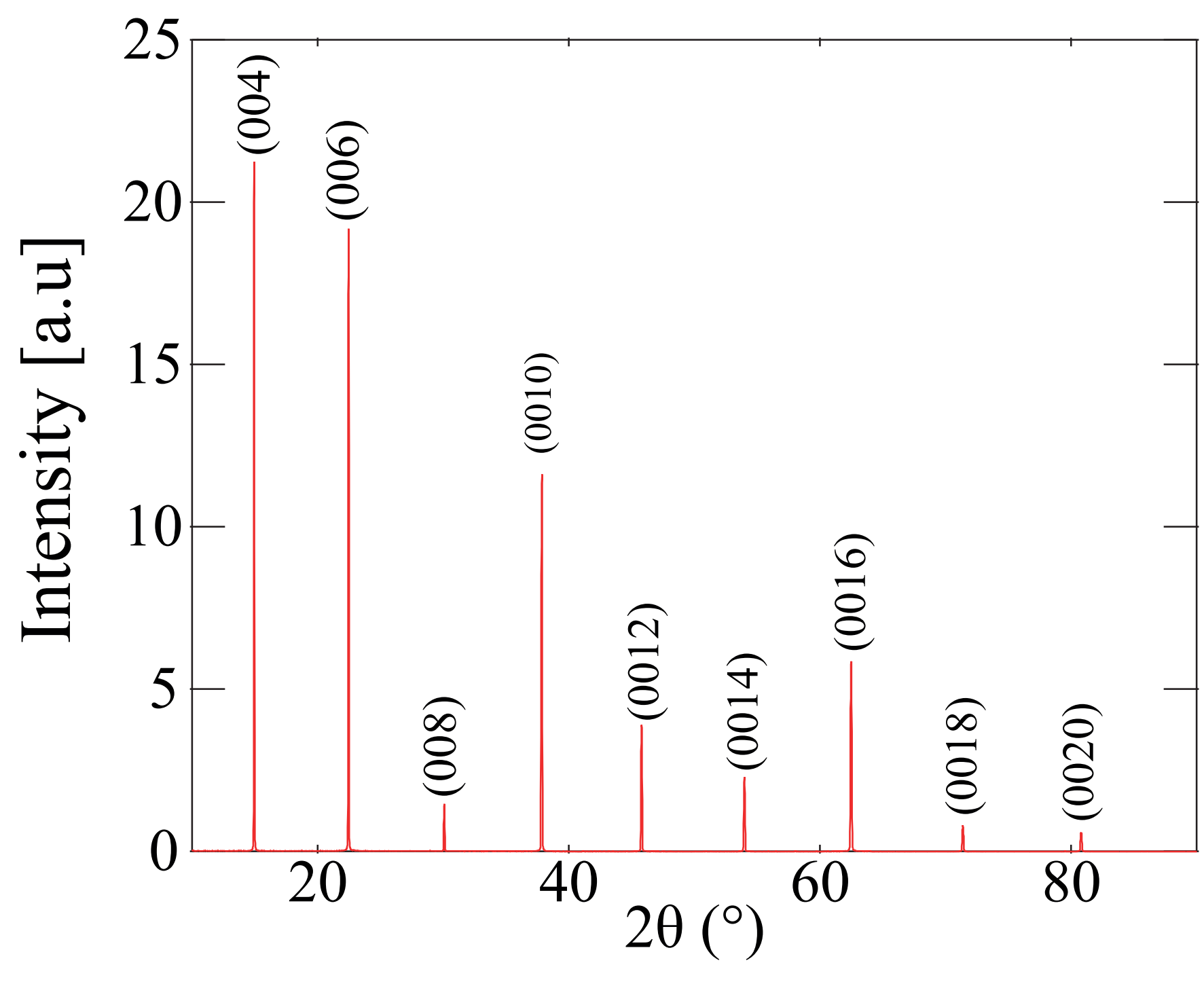

Supplementary Figure 1: Diffraction data from a $(SnS)_{1.15}(TaS_2)$ single crystal. X-rat diffraction lines for $(SnS)_{1.15}(TaS_2)$ samples measured using Cu K_α ($\lambda = 1.54056$ Å) radiation. The $(00l)$ reflections indicate the plane of the single crystal is perpendicular to the crystallographic c-axis and the sharpness of the peaks demonstrates the good quality of the single crystals.

II. RESISTIVITY

Closely observing the resistivity data for 5 K $\leq$ T $\leq$ 300 K shows a saturating resistivity at high temperatures, Supplementary Figure 2. Resistivity saturation is often observed in materials with multiple conduction channels, in particular for many intermetallic materials [1]. This characteristic behavior can be well described by a parallel resistivity model. At high temperatures, due to increased thermal energy, the mean free path of the electrons decreases and becomes close to a few interatomic spacings. The dominant scattering mechanism at these temperatures is due to electron-phonon interaction. The scattering cross section will no longer be linear in scattering perturbation. So the resistivity will not be proportional to mean square atomic displacement, which is proportional to temperature for harmonic potentials. Hence, with further increase in temperature, the slope of $\rho(T)$ is decreasing and eventually resistivity saturates. For a metallic material, $\rho(T)$ can be written as $\rho_i(T) = \rho_{i,0} + \rho_{i,L}(T)$, where the first term indicates the residual resistivity that arises from impurities and disorder, which is temperature-independent. The second term addresses the temperature-dependent general resistivity which can be written according to the Bloch-Gruneison model as

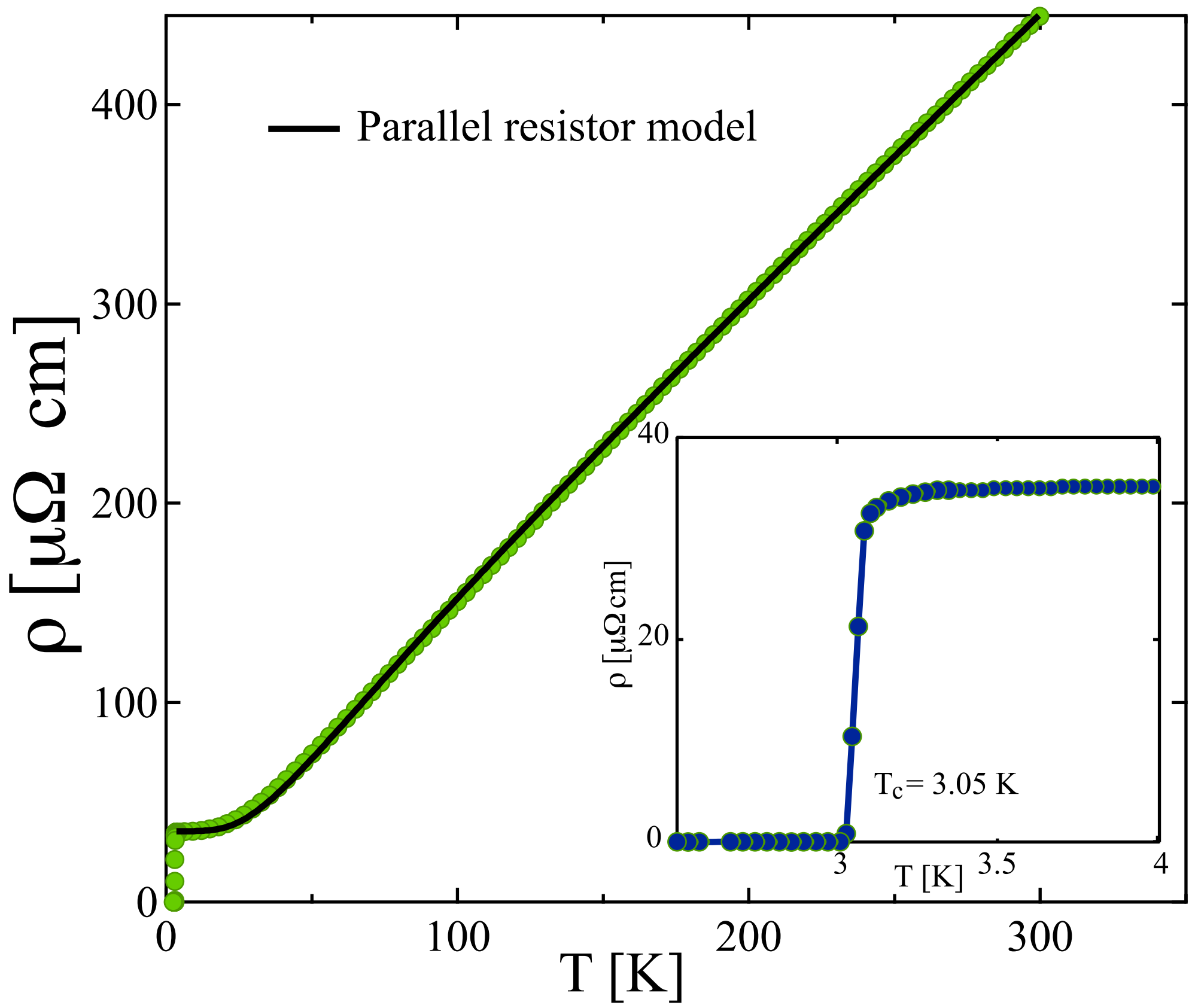

Supplementary Figure 2: Resistivity data of $(SnS)_{1.15}(TaS_2)$. Electrical resistivity for the $(SnS)_{1.15}(TaS_2)$ in the range 1.7 K $\geq T \geq$ 300 K. The dotted line shows the fit to the data using the parallel resistor model. The inset shows the expanded view, showing the drop in resistivity around 3.05 K.

$$\rho_{i,L}(T) = C\left(\frac{T}{\Theta_D}\right)^5 \int_0^{\Theta_D/T} \frac{x^5}{(e^x-1)(1-e^{-1})}dx \tag{1}$$

where Θ_D is the Debye temperature and C is the material-dependent prefactor. Considering the resistivity saturation at high temperatures, the resistivity curve can be explained by

$$\rho(T) = \left[\frac{1}{\rho_s} + \frac{1}{\rho_i(T)}\right]^{-1} \tag{2}$$

where ρ_s is the saturation value of resistivity at high temperatures. The data show an excellent fit of this model as shown in Fig.2a, and yields Debye temperature $\Theta_D = 168 \pm 2$ K, C = 3.7 ± 5 $m\Omega$cm, residual resistivity $\rho_{i,0} = 145$ ± 1 $\mu\Omega$cm, $\rho_s = 23302 \pm 4$ $\mu\Omega$cm.

III. CRITICAL FIELD FITTING

To fit the critical fit data as in Fig.2c, we make use of the following parametric equation to extract the behavior, and extrapolated critical fields at T=0K.

$$ln\left(\frac{1}{t}\right) = \left[U(s) + U(\eta s) + \frac{\lambda_0}{w}\right] + \left(\frac{1}{4}\left[U(s) - U(\eta s) - \frac{\lambda_-}{w}\right]^2 + \frac{\lambda_{12}\lambda_{21}}{w^2}\right)^{1/2} \tag{3}$$

$$H_{c2} = \frac{2\phi_0 T s}{D_e} \qquad \eta = \frac{D_h}{D_e} \qquad U(s) = \psi(s+1/2) - \psi(1/2)$$

Here, $\lambda_- = \lambda_{11} - \lambda_{22}$, $\lambda_0 = (\lambda_-^2 + 4\lambda_{12}\lambda_{21})$, $w = \lambda_{11}\lambda_{22} - \lambda_{21}\lambda_{12}$. The variables, $\lambda_{11}, \lambda_{22}, \lambda_{21}, \lambda_{21}$ represents the matrix elements of the BCS coupling constants. D_1 and D_2 are the electron and hole diffusivity, respectively. ϕ_0 is the flux quantum and $\psi(s)$ is the digamma function. The fitting of the experimental data using this equation, and extrapolating the upper critical field to T=0 K gives 0.59 T for out of plane magnetic field and 10.68 T for an in-plane field. The table I shows the fitting parameters obtained. The fitting has converged to $\lambda_{22} = \lambda_{11}$, $\lambda_{12} = \lambda_{21}$ with λ_{11} higher than λ_{12}, indicating the mostly decoupled nature of the bands. $T_c = 3.05$ K was kept fixed during the fitting.

type	D_1	η	λ_{11}	λ_{12}
$H_{c2\parallel c}$	11.01	0.19	0.88	0.35
$H_{c2\parallel ab}$	0.67	0.19	1.37	0.49

TABLE I: Fitting parameters from two-gap model for H_{c2}

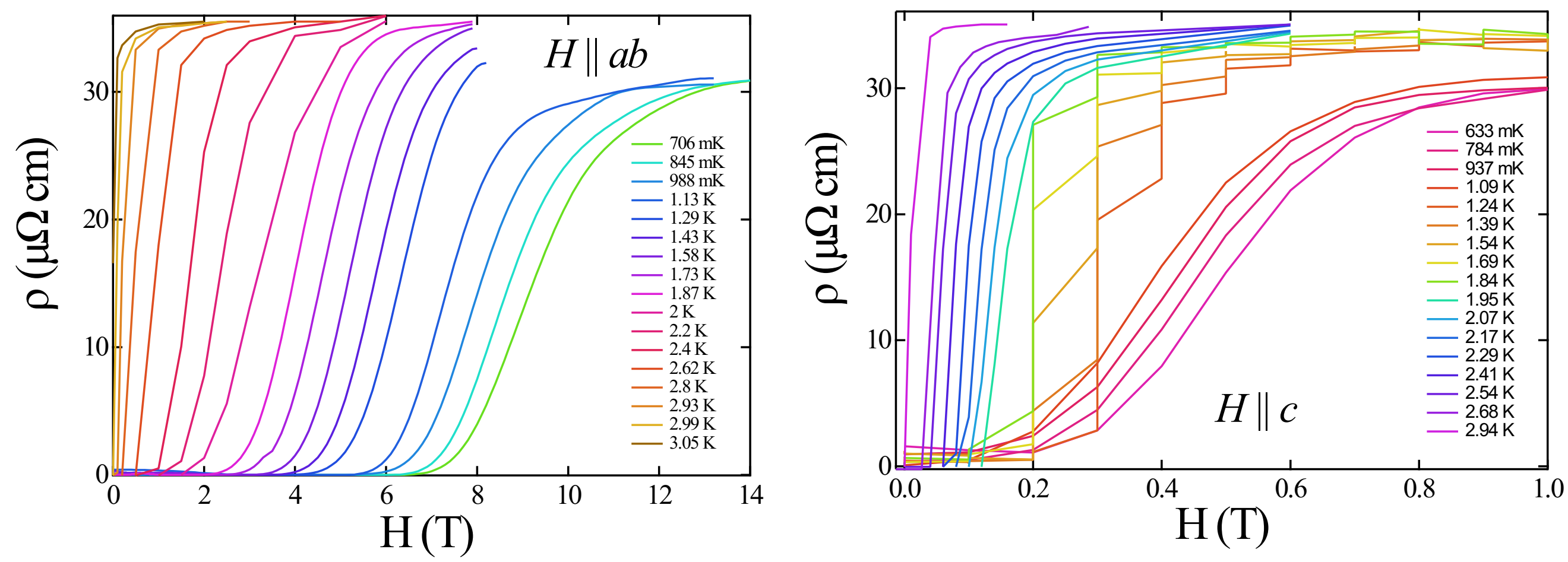

Supplementary Figure 3: **Resistivity data under applied magnetic field.** Resistivity vs field data taken at different temperatures for in-plane and out of plane direction from a $(SnS)_{1.15}(TaS_2)$ single crystal. Data was acquired using a Physical property measurement system (PPMS).

IV. ADDITIONAL ARPES DATA

By varying the photon energy in an ARPES experiment, we can access different values of k_z and thereby track the out-of-plane band dispersion [2–4]. Supplementary Figure 4 shows the measured dispersion along the M-L line for both the SnS-TaS_2 misfit compound and the pristine 2H-TaS_2 sample.

To quantify the effect of a possible inter-layer coupling between the two 1H-TaS_2 layers in the misfit compound, we start from the single-layer 1H-TaS_2 tight-binding Hamiltonian $H_0(\mathbf{k}_\parallel)$ [5], which includes the d_{z^2}, d_{xy}, and $d_{x^2-y^2}$ orbitals and incorporates spin-orbit coupling via the term $\frac{\lambda}{2}\,\sigma_z \otimes L_z$. Here σ_i are Pauli matrices in spin space, L_z is the orbital angular-momentum operator in the three-orbital basis, and λ is the spin-orbit coupling strength.

To describe two 1H layers per unit cell, we introduce a layer-pseudospin represented by Pauli matrices τ_i, where $\tau_z = \pm 1$ labels the upper and lower 1H-TaS_2 layers. A possible inter-layer hopping between identical orbitals and identical spin components on the two layers is incorporated through the full Hamiltonian

$$H(k_x, k_y, k_z) = \tau_0 \otimes \sigma_0 \otimes H_0(k_x, k_y) + \frac{\lambda}{2}\,\tau_0 \otimes \sigma_z \otimes L_z + t\cos(k_z c)\,\tau_x \otimes \sigma_0 \otimes I_{3\times 3}, \tag{4}$$

where t is the inter-layer hopping amplitude, c is the lattice spacing along the z direction, and $I_{3\times 3}$ is the identity in orbital space.

We evaluate the eigenvalues of this Hamiltonian at the in-plane M point $(k_x, k_y) = (k_\mathrm{M})$ while varying k_z from 0 to $\pi/(2c)$, corresponding to the experimental M-L trajectory probed in the photon-energy-dependent ARPES measurements. We start by calculating the dispersion for $t = 0$ meV corresponding to vanishing interlayer coupling where the bands are k_z-independent, and no gap opens along the M-L line, Supplementary Figure 4,a, and comparing it to experimental data, Supplementary Figure 4,b. In Supplementary Figure 4,c we use $t = 65$ meV, the interlayer hopping amplitude previously extracted from ARPES measurements for bulk 2H-TaS_2 [2], and from transport data [6]. In this case the calculation shows a clear k_z-dependent bonding-antibonding splitting whose magnitude is maximal at the M point and decreases towards L.

Comparing these two limiting cases with the experimental data in Supplementary Figure 4b and d, we find that the measured bands show no detectable k_z-dependent splitting and are only compatible with the $t = 0$ calculation, demonstrating that the effective inter-layer coupling between the two 1H-TaS_2 layers in the misfit compound is negligible.

In Supplementary Fig. 5 we show a simulation of the expected EDCs at the high symmetry points M (blue) and L (red) of the Brillouin zone. The simulation is for the case of a $t = 15$ meV with experimental parameters as extracted in previous studies [2]. The clear change in the EDC shape and the dispersion along k_Z is a hallmark of finite inter-layer coupling, and stand in contrast to the data acquired and shown in Supplementary Figure 4. The small value of the inter-layer coupling used, emphasize our ability to determine that the inter-layer coupling in the SnS-TaS2 is

negligibly small, constrained to be below 10 meV.

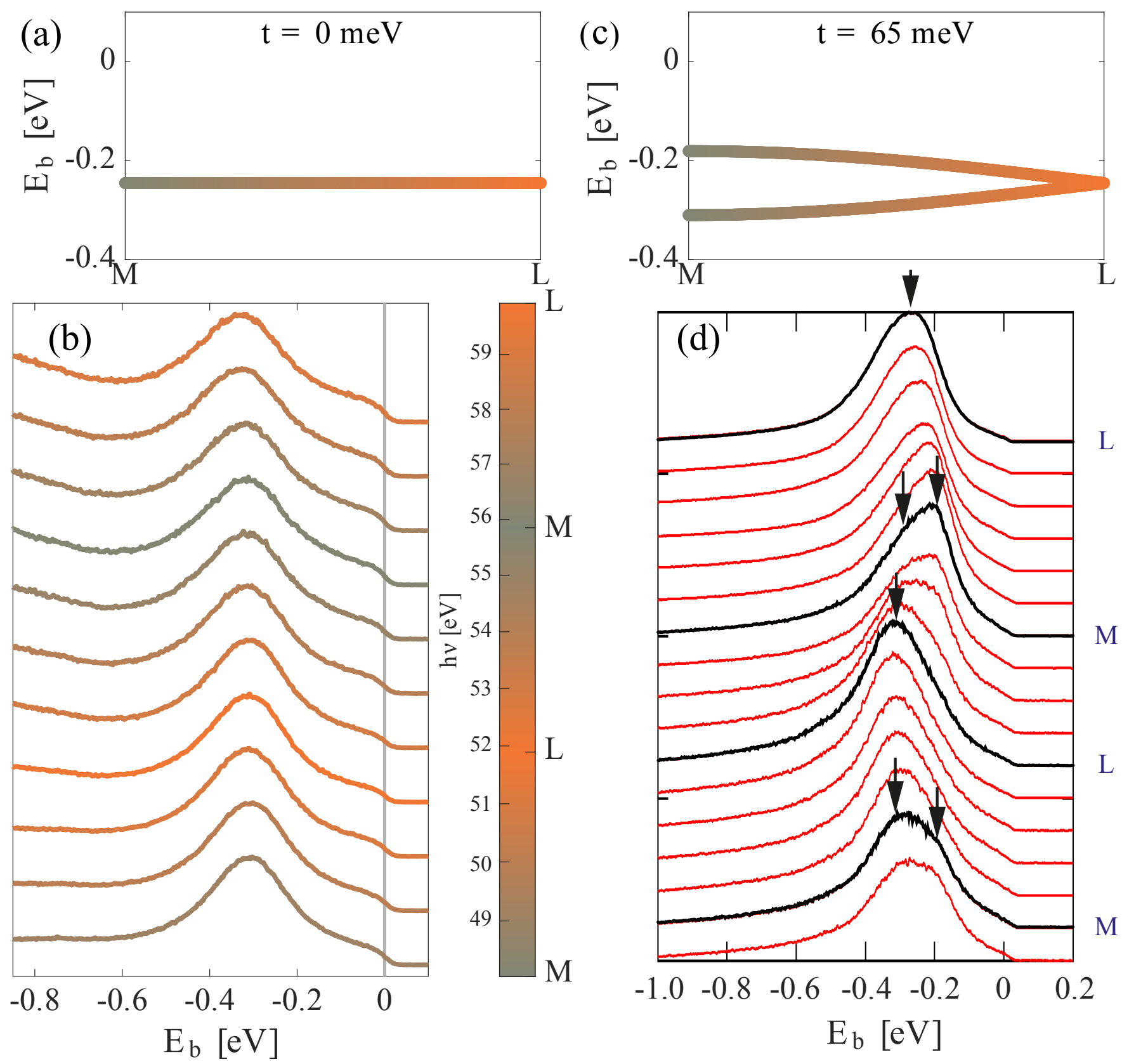

Supplementary Figure 4: Lack of k_z dependence in ARPES data. (a) Calculated band dispersion along the M-L line for an inter layer hopping of $t = 0$ meV, showing the absence of any k_z dependent gap. (b) Energy distribution curves (EDCs) taken along the M-L direction of the 1H-TaS_2 Brillouin zone. The different k_z values are accessed by varying the photon energy from 49 to 59 eV, covering approximately one and a half Brillouin zones along k_z. The gray line marks the Fermi level. (c) Same calculation as in a for $t = 65$ mev, showing a k_z dependent bonding antibonding splitting. The comparison demonstrates that the experimental data are consistent with a vanishing effective inter layer coupling between two consecutive 1H layers in the misfit compound. (d) EDCs taken along the M-L direction of the 2H-TaS_2 Brillouin zone.

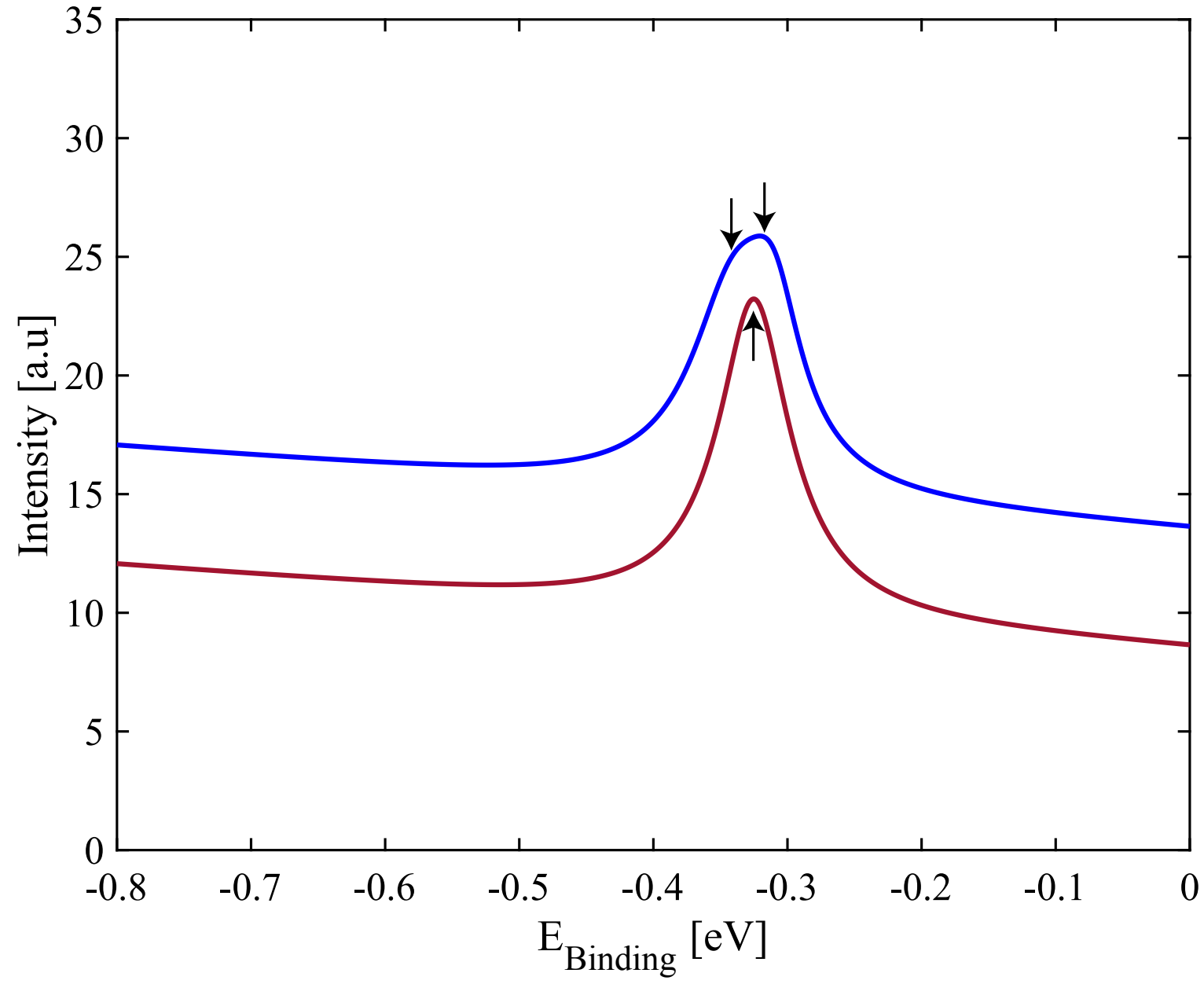

Supplementary Figure 5: Simulation of the expected EDC with small inter-layer coupling at the high symmetry points along k$_z$. Simulation of the expected EDCs at the M point (blue) and L point (red) of the Brillouin zone. The calculation is performed for t=15 meV, showing a k_Z dependent bonding-antibonding splitting at the M point. The simulation incorporates realistic parameters extracted from angle-resolved photoemission spectroscopy experiment, based on previous measurements and fitting results [2]. A clear dispersion is expected and observed upon varying the photon energy to span more than a full Brillouin zone. This pronounced dispersion demonstrates our sensitivity to inter-layer coupling above 10 meV, thereby supporting our estimate that the inter-layer coupling is less than 10 meV.

V. SUPERCONDUCTING GAP

A. Gap Fitting

We tried two simple models to fit the superconducting gap, the first was a single isotropic gap that yields poor results, Supplementary Figure 6. For the fit with a single-gap we use the function[7]:

$$\frac{dI}{dV}(V) = \int_{-\infty}^{\infty} dE \rho_0 N_0 \left| \Re\left[\frac{E}{\sqrt{E^2-\Delta^2}}\right]\right| \left(\frac{df(E+V,T_{eff})}{dV}\right) \tag{5}$$

where f is the Fermi-Dirac distribution, V is the bias voltage, and Δ is the superconducting gap.

The two component model which was used is given by:

$$\frac{dI}{dV}(V) = \frac{1}{2\pi}\int_0^{2\pi} d\theta \int_{-\infty}^{\infty} dE \rho_0 \left(N_0 \left| \Re\left[\frac{E}{\sqrt{E^2-(\Delta_0+\Delta_1 cos(3\theta))^2}}\right]\right|\right) \left(\frac{df(E+V,T_{eff})}{dV}\right) \tag{6}$$

where f is the Fermi-Dirac distribution, V is the bias voltage, and $\Delta_{1,2}$ are the different components. It should be noted that the fit has excellent agreement and we used only the effective temperature (510 mK) and the energy of the two components as fitting parameters. This asserts to the robustness of our model.

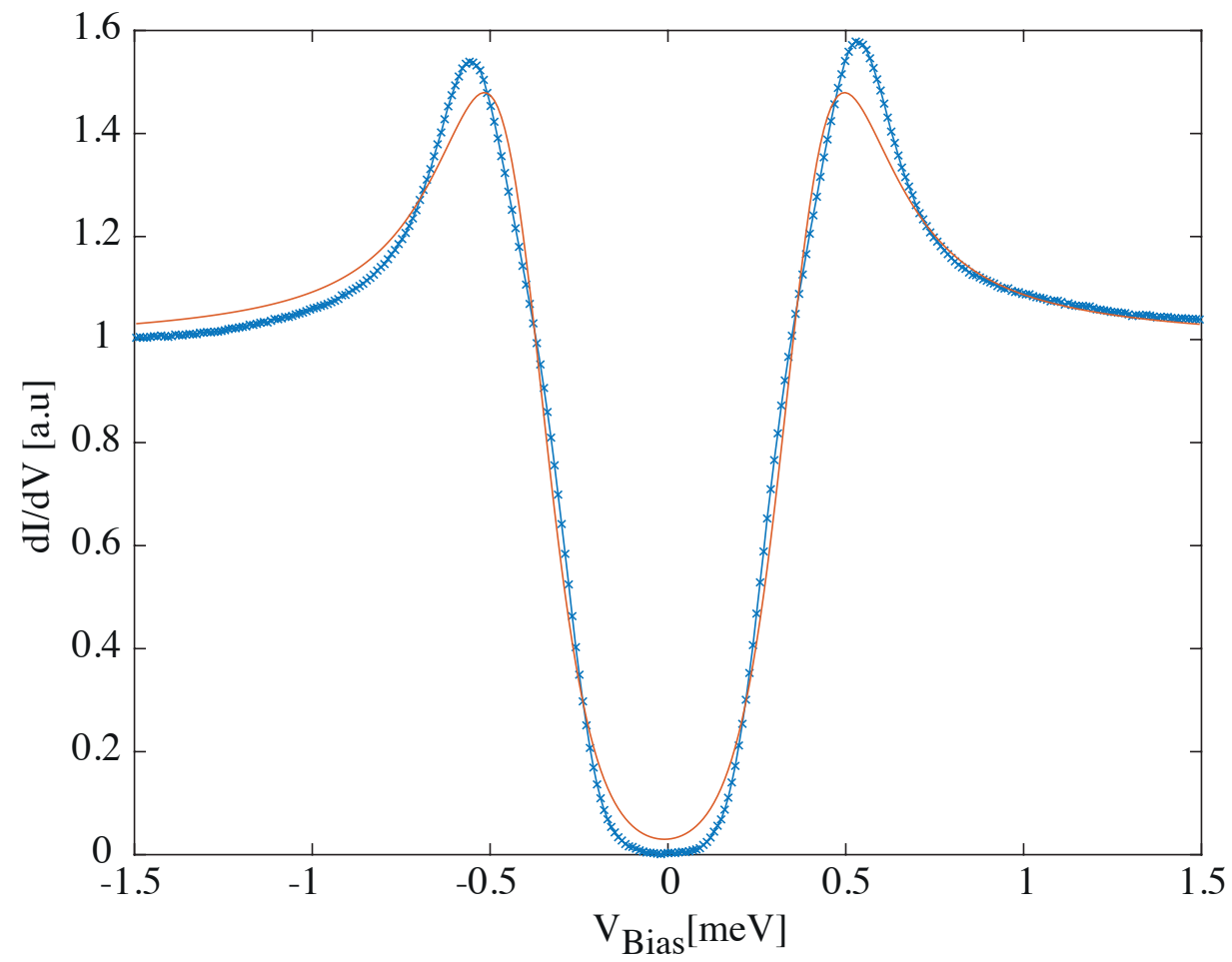

Supplementary Figure 6: **Single gap fitting of the STM spectra in the superconducting state**. Fitting the SC gap to a single BCS gap. Besides the residual denity of states arising from the considerably high effective temperature (900 mK) given by the fit, the slope of the DOS curve does not match the measured one, emphasizing the two-gap behavior measured on the 1H layer.

B. Gap Uniformity

The superconducting gap in the sample is extremely uniform, indicating a high quality clean sample. Moreover, it indicates that the adatoms coming after the cleave process do not alter the electronic properties, and do not interact with the superconducting phase.

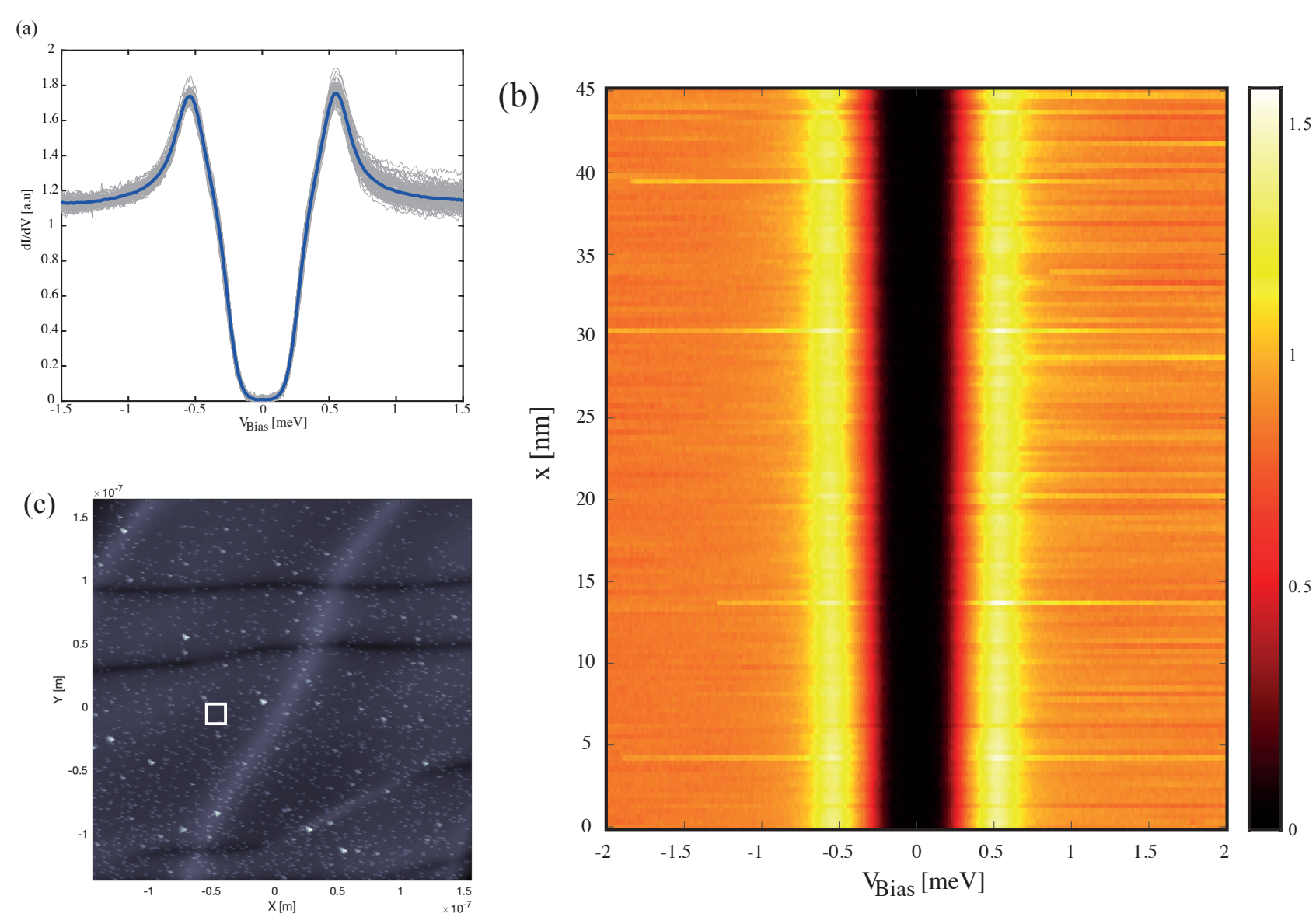

Supplementary Figure 7: **Uniformity of the gap along a linecut taken away from the large scale lines.** (a) The superconducting gap in the 1H layer taken as an average of 150 different points. The blue curve is the average, and the light gray curves are the different spectra at different locations along a 45 nm line. I_{set} = 50 pA and V_{Bias} = -2meV, T=0.3K (b) False color plot showing the uniformity of the gap. The data is the same as in (a). (c) Large scale topography showing the location of the line cut with respect to large scale topography lines.

VI. VORTEX IN THE TIN-SULFUR LAYER

we show the dI/dV map at E=0 in the SnS layer, at B=0.1T. The distance between the bright spots matches that expected from a B=0.1 T magnetic field (150 nm). The dI/dV spectra of the line cut as a function of position are seen in Supplementary Figure 8b. A fit to the filling of the DOS in the gap has produced $\xi = 45 \pm 10$nm, a coherence length that matches that extracted from H_{c2} measurements. The dI/dV map at the Fermi level and other line cuts, not shown here, give an isotropic vortex shape. This evidence shows that the gap in the SnS layer is of superconducting origin and is intrinsic to the layer as opposed to a proximity gap due to the 1H layer.

A clear filling of the gap is seen in the vortex core, with an increase in the density of states at its center. This extra dI/dV signal at the vortex core is probably due to Caroli-de Gennes-Matricon states, which is expected to exist in the core of every superconducting vortex.

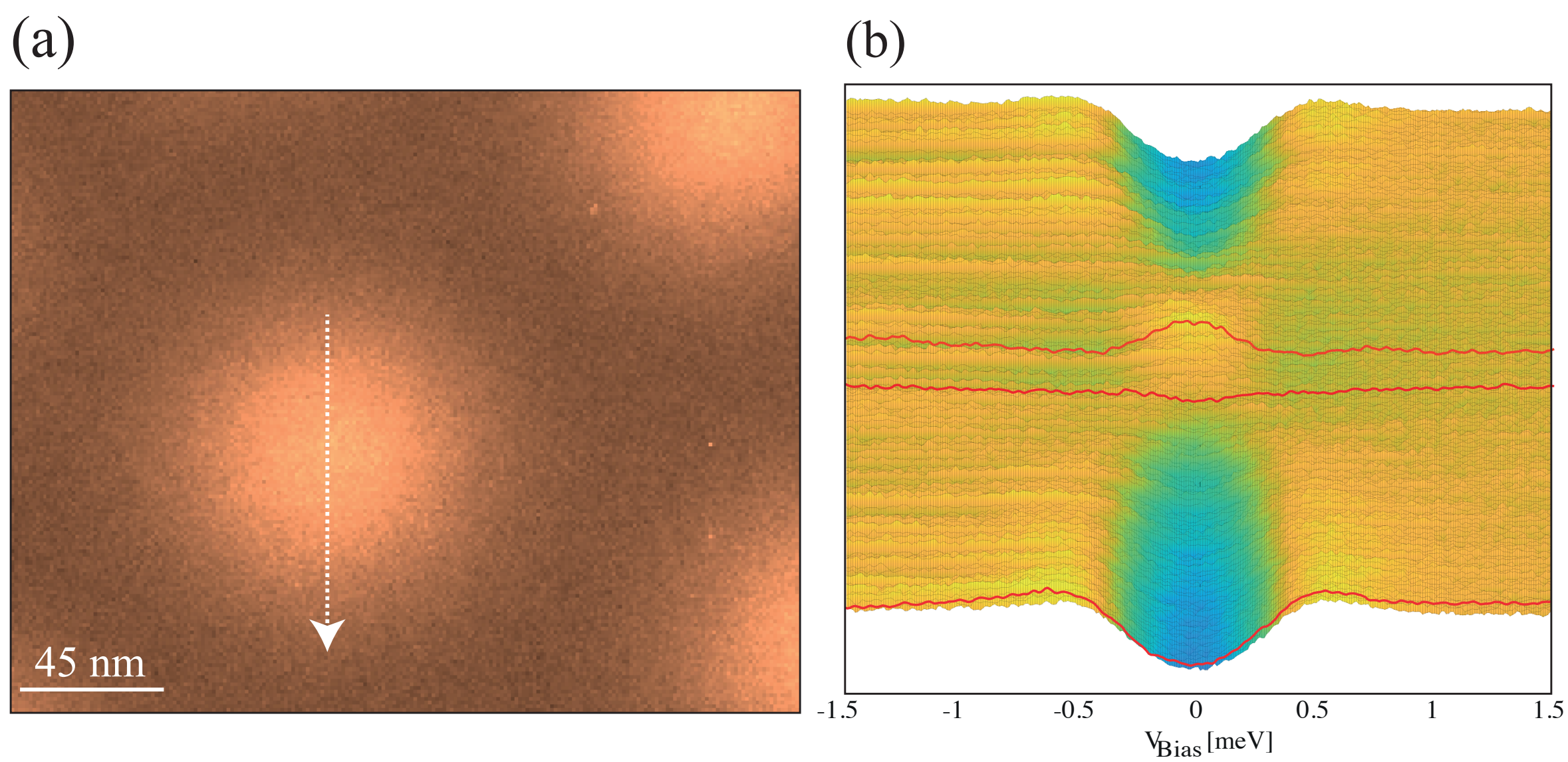

Supplementary Figure 8: **Imaging a vortex in the SnS layer**. (a) dI/dV map at E=0 acquired on a SnS layer at T=0.3K and B=0.1T (I_{set}=35pA, V_{Bias} = -5mV). The vortex map shows that the gap in the SnS layer is of superconducting origin. Arrow mark the line cuts taken on the vortex at high resolution. (b) dI/dV spectra taken along line cut, and shows the enhanced density of states at the vortex core.

A. Magnetic field dependence of the gap

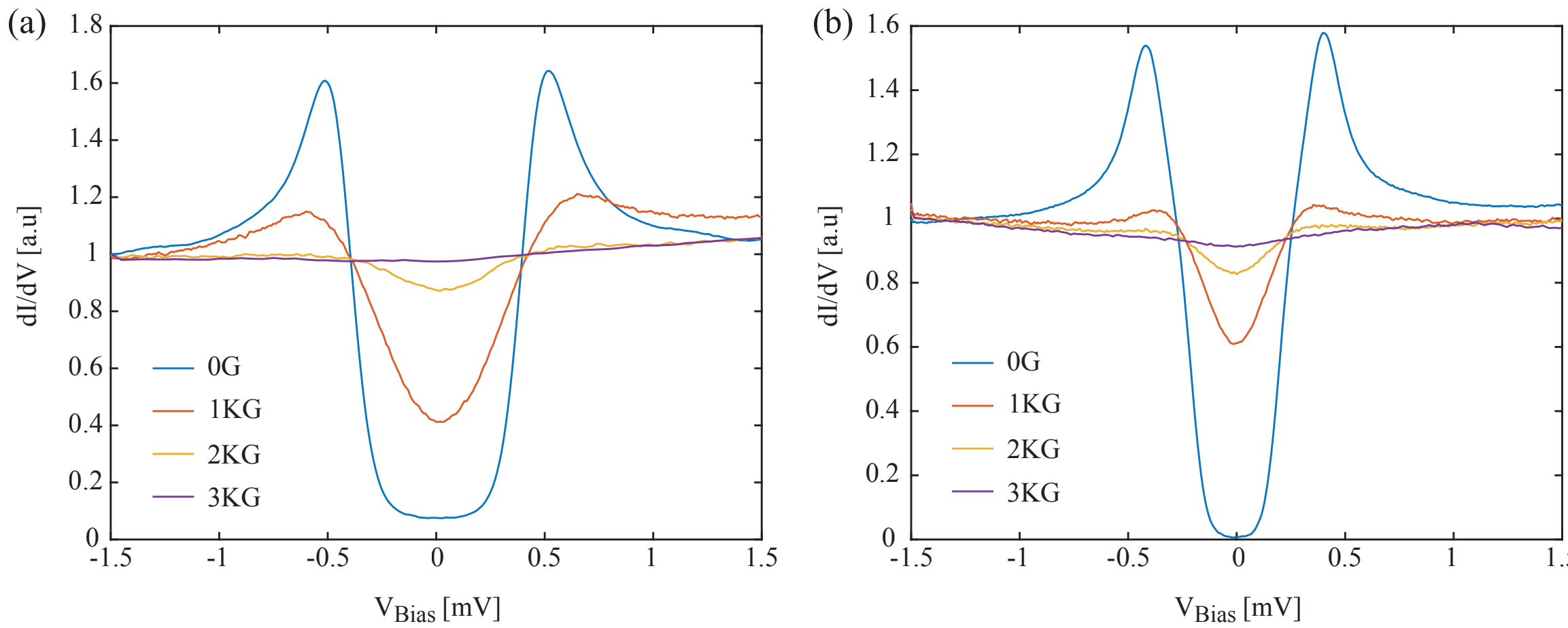

Supplementary Figure 9: **Magnetic field dependence of the STM spectra on the 1H and SnS layers**. Magnetic field dependence of the superconducting gap as measured in the SnS (a) and 1H-TaS_2 (b) layers, for B $\parallel$ c. The critical field measured is 3KG for both layers, and have excellent agreement with the critical field measured using transport at this effective temperature (T$\sim$ 0.5K).

VII. LACK OF EDGE-EFFECT ON THE GAP

As described in the main text, an edge is a strong scatterer for surface electrons. Therefore, for any mechanism which includes scattering from different bands in the Brillouin zone we would expect a strong signal of that mechanism. This includes the McMillan process in which the unique spectroscopic signature arise from scattering of quasi-particles between bands which possess superconductivity gaps. Thus, the spectroscopic signal from such a mechanism is expected to be stronger near the edge, yet no change is seen in the acquired spectroscopy on and off the edge. This experimental observation indicates that a mechanism based on scattering is unlikely to explain our superconducting spectra.

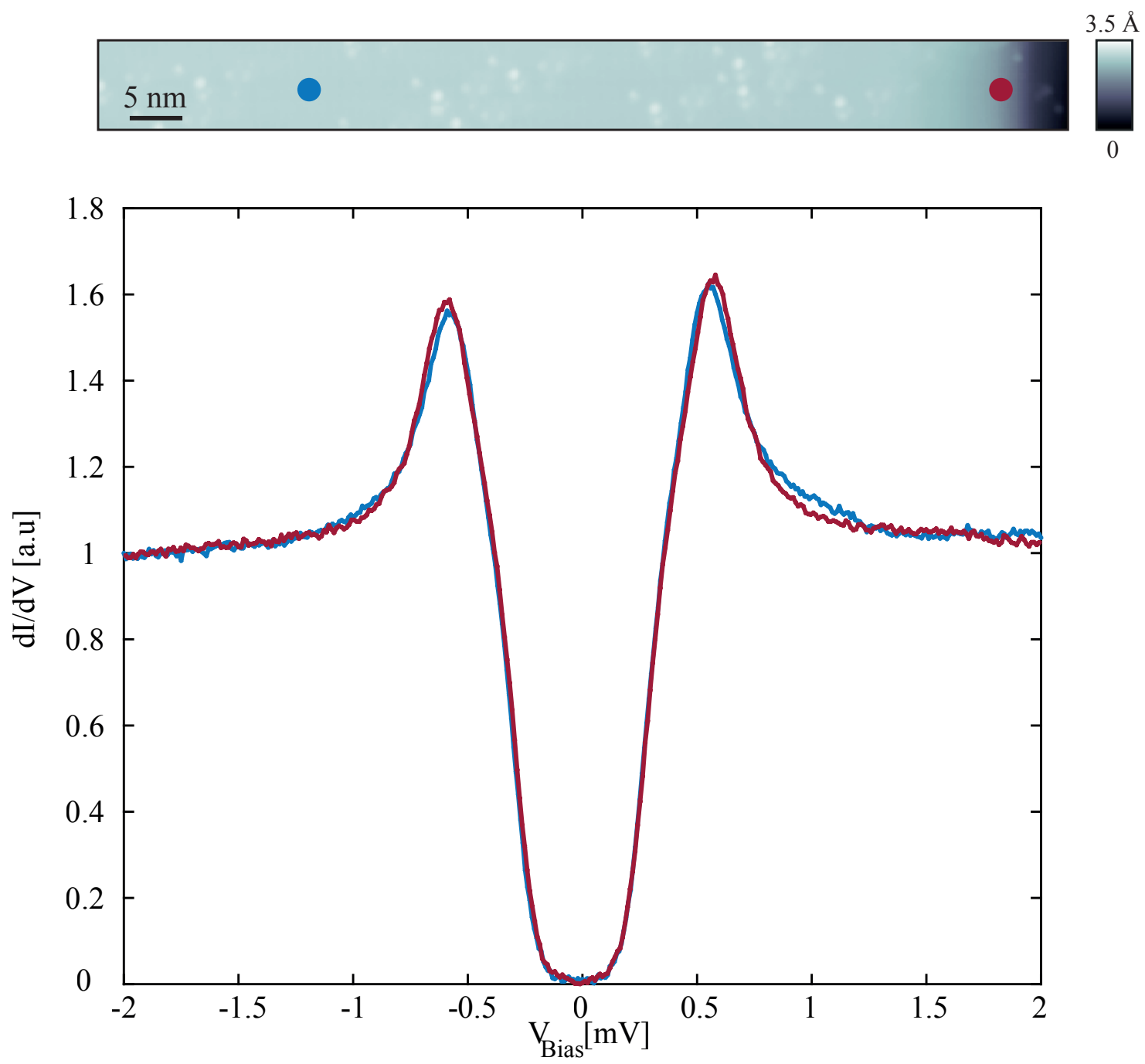

Supplementary Figure 10: **Additional STM spectra takn on and off a 1H layer edge.** Topography of a step edge from a 1H-TaS_2 layer to a SnS layer. The blue and red dots represents the locations where the respective dI/dV spectra was acquired. The dI/dV spectra on and off the edge show no change with no filling of the gap or a change in the slope of the spectra, pointing on lack of influence of edge. Such lack of influence point on the fact that the spectra shape is not influenced by electron scattering such as in the McMillan model. $I_{\rm set} = 100$ pA, $V_{\rm Set} = -2$ mV and $V_{\rm mod} = 20$ μV, $f = 870$ Hz.

VIII. THEORETICAL MODEL

In this supplement we analyze the effect of interlayer coupling on the triplet-singlet mixing in TMD superconductors of the H type (2H-TaS_2, 2H-$NbSe_2$, 2H-$TaSe_2$, etc.). We will consider a single bilayer with finite-ranged attractive interactions and tune the strength of the interlayer hopping. We then investigate how the "mutli-gap" features, e.g. in the tunneling density of states, depend on the coupling.

A. Model

For simplicity we consider a toy model that contains much of the physics of a single bilayer of 2H-TaS_2 with a finite range intralayer attractive interaction

$$H = \sum_{\boldsymbol{k}} c^\dagger_{\boldsymbol{k}} \left[t\epsilon_{\boldsymbol{k}} - \lambda\, o_{\boldsymbol{k}} \sigma^z \tau^z - t_\perp \tau_x \right] c_{\boldsymbol{k}} - \frac{1}{2} \sum_{\boldsymbol{kp}} \sum_{\sigma\sigma'} \sum_{\alpha} V_{\boldsymbol{kp}} c^\dagger_{\boldsymbol{k}\sigma\alpha} c^\dagger_{-\boldsymbol{k}\sigma'\alpha} c_{-\boldsymbol{p}\sigma'\alpha} c_{\boldsymbol{p}\sigma\alpha} \tag{7}$$

where $\alpha = 1, 2$ denotes the layer, $\sigma = \uparrow\downarrow$, denotes spin and the Pauli matrices $\tau^{x,y,z}$ and $\sigma^{x,y,z}$ are in the respective basis. The dispersion functions $\epsilon_{\boldsymbol{k}}$ and $o_{\boldsymbol{k}}$ are given by

$$\epsilon_{\boldsymbol{k}} = \cos(\boldsymbol{T}_1 - \boldsymbol{T}_2) \cdot \boldsymbol{k} + \cos(\boldsymbol{T}_3 - \boldsymbol{T}_2) \cdot \boldsymbol{k} + \cos(\boldsymbol{T}_3 - \boldsymbol{T}_1) \cdot \boldsymbol{k}$$

$$o_{\boldsymbol{k}} = \sin \boldsymbol{T}_1 \cdot \boldsymbol{k} + \sin \boldsymbol{T}_2 \cdot \boldsymbol{k} + \sin \boldsymbol{T}_3 \cdot \boldsymbol{k}$$

where $\boldsymbol{T}_1 = -a\hat{\boldsymbol{y}}$, $\boldsymbol{T}_2 = a(-\sqrt{3}\hat{\boldsymbol{x}} + \hat{\boldsymbol{y}})/2$ and $\boldsymbol{T}_3 = a(\sqrt{3}\hat{\boldsymbol{x}} + \hat{\boldsymbol{y}})/2$ are the vectors to the three nearest neighbors. Therefore, t is an energy scale representing the hopping, λ is the strength of Ising spin-orbit coupling and $t_\perp$ is the interlayer hopping strength. The dispersion part of the Hamiltonian Eq. (7) is tailored to have a band structure very similar to the conduction band of TMD metals (see Supplementary Figures 6,7,8).

The interaction term is assumed to be

$$V_{\boldsymbol{kp}} = V_0 + V_1 e_{\boldsymbol{k}-\boldsymbol{p}} + V_2 \epsilon_{\boldsymbol{k}-\boldsymbol{p}} \tag{8}$$

where

$$e_{\boldsymbol{k}} = \cos \boldsymbol{T}_1 \cdot \boldsymbol{k} + \cos \boldsymbol{T}_2 \cdot \boldsymbol{k} + \cos \boldsymbol{T}_3 \cdot \boldsymbol{k}\,.$$

Thus, the term V_0 represents the on-site interaction, V_1 the nearest neighbor interaction and V_2 the next-to-nearest neighbor interaction. In what follows, we will see that a finite range interaction is an essential ingredient to get multi-gap features.

B. BCS theory

In the spirit of BCS theory we first transform the Hamiltonian to the band basis. This is done using the transformation

$$c_{\boldsymbol{k}\sigma\alpha} = \sum_j U_{\alpha j}(\sigma \boldsymbol{k}) \psi_{\boldsymbol{k}\sigma j}$$

where $\sigma = +1$ for $\uparrow$ and -1 for $\downarrow$, $j = \pm$ denotes the bands and

$$\hat{U}(\boldsymbol{k}) = \begin{pmatrix} \cos\frac{\theta_{\boldsymbol{k}}}{2} & \sin\frac{\theta_{\boldsymbol{k}}}{2} \\ \sin\frac{\theta_{\boldsymbol{k}}}{2} & -\cos\frac{\theta_{\boldsymbol{k}}}{2} \end{pmatrix}$$

Here

$$\cos\theta_{\boldsymbol{k}} = -\frac{t_\perp}{A_{\boldsymbol{k}}} \;\; ; \;\; \sin\theta_{\boldsymbol{k}} = -\frac{\lambda o_{\boldsymbol{k}}}{A_{\boldsymbol{k}}} \;\; ; \;\; A_{\boldsymbol{k}} = \sqrt{\lambda^2 o^2_{\boldsymbol{k}} + t^2_\perp}$$

Using this transformation we find that the Hamiltonian Eq. (7) assumes the form

$$H = \sum_{\boldsymbol{k}\sigma j} \zeta_{\boldsymbol{k}j}\, \psi^\dagger_{\boldsymbol{k}\sigma j}\psi_{\boldsymbol{k}\sigma j} - \sum_{\boldsymbol{kp}}\sum_{\sigma\sigma'}\sum_{j_1j_2j_3j_3} \tilde{V}^{j_1j_2j_3j_4}_{\boldsymbol{kp};\sigma\sigma'}\psi^\dagger_{\boldsymbol{k}\sigma j_1}\psi^\dagger_{-\boldsymbol{k}\sigma' j_2}\psi_{-\boldsymbol{p}\sigma' j_3}\psi_{\boldsymbol{p}\sigma j_4} \tag{9}$$

where $\zeta_{\boldsymbol{k}\pm} = t\epsilon_{\boldsymbol{k}} \pm A_{\boldsymbol{k}}$ and

$$\tilde{V}^{j_1j_2j_3j_4}_{\boldsymbol{kp};\sigma\sigma'} = \frac{1}{2}\sum_\alpha U^*_{\alpha j_1}(\sigma\boldsymbol{k})U^*_{\alpha j_2}(-\sigma'\boldsymbol{k})U_{\alpha j_3}(-\sigma'\boldsymbol{p})U_{\alpha j_4}(\sigma\boldsymbol{p})V_{\boldsymbol{kp}}$$

Here we will limit ourselves to *intraband pairing only*. Furthermore, due to the Ising spin-orbit coupling we consider only pairing states between spin up at one momentum and spin down in the opposite momentum (spin along z remains a good quantum number). Consequently, the following product

$$U_{\alpha j}(-\sigma'\boldsymbol{k})U_{\alpha\ell}(\sigma\boldsymbol{k}) = U_{\alpha j}(\boldsymbol{k})U_{\alpha\ell}(\boldsymbol{k})$$

becomes spin-independent and so does the interaction. We then decompose the interaction into symmetric and anti-symmetric parts

$$\tilde{V}^{(s)}_{\boldsymbol{kp};j\ell} = \frac{1}{4}\left[\tilde{V}^{jj\ell\ell}_{\boldsymbol{kp}} + \tilde{V}^{jj\ell\ell}_{-\boldsymbol{kp}} + \tilde{V}^{jj\ell\ell}_{\boldsymbol{k},-\boldsymbol{p}} + \tilde{V}^{jj\ell\ell}_{-\boldsymbol{k},-\boldsymbol{p}}\right]$$

$$\tilde{V}^{(a)}_{\boldsymbol{kp};j\ell} = \frac{1}{4}\left[\tilde{V}^{jj\ell\ell}_{\boldsymbol{kp}} - \tilde{V}^{jj\ell\ell}_{-\boldsymbol{kp}} - \tilde{V}^{jj\ell\ell}_{\boldsymbol{k},-\boldsymbol{p}} + \tilde{V}^{jj\ell\ell}_{-\boldsymbol{k},-\boldsymbol{p}}\right]$$

and use the following BCS mean-field decoupling to the interaction term,

$$\begin{aligned}\Delta^{(s)}_j(\boldsymbol{k}) &= \sum_{\boldsymbol{p}\ell} V^{(s)}_{\boldsymbol{kp};j\ell}\left[\langle\psi_{-\boldsymbol{p}\downarrow\ell}\,\psi_{\boldsymbol{p}\uparrow\ell}\rangle - \langle\psi_{-\boldsymbol{p}\uparrow\ell}\,\psi_{\boldsymbol{p}\downarrow\ell}\rangle\right]\\ \Delta^{(a)}_j(\boldsymbol{k}) &= \sum_{\boldsymbol{p}\ell} V^{(s)}_{\boldsymbol{kp};j\ell}\left[\langle\psi_{-\boldsymbol{p}\downarrow\ell}\,\psi_{\boldsymbol{p}\uparrow\ell}\rangle + \langle\psi_{-\boldsymbol{p}\uparrow\ell}\,\psi_{\boldsymbol{p}\downarrow\ell}\rangle\right]\,.\end{aligned} \tag{10}$$

The above decoupling leads to the following BdG Hamiltonian

$$H^j_{\mathrm{BdG}} = \begin{pmatrix} \zeta_{\boldsymbol{k}j} & -\Delta^{(s)}_j(\boldsymbol{k}) - \Delta^{(a)}_j(\boldsymbol{k}) & & \\ -\Delta^{(s)}_j(\boldsymbol{k}) - \Delta^{(a)}_j(\boldsymbol{k}) & -\zeta_{-\boldsymbol{k}j} & & \\ & & \zeta_{\boldsymbol{k}j} & -\Delta^{(s)}_j(\boldsymbol{k}) + \Delta^{(a)}_j(\boldsymbol{k}) \\ & & -\Delta^{(s)}_j(\boldsymbol{k}) + \Delta^{(a)}_j(\boldsymbol{k}) & -\zeta_{-\boldsymbol{k}j}\end{pmatrix} \tag{11}$$

which is written in the Nambu basis $\Psi_{\boldsymbol{k}} = [\psi_{\boldsymbol{k}\uparrow j}, \psi^\dagger_{-\boldsymbol{k}\downarrow j}, \psi_{\boldsymbol{k}\downarrow j}, -\psi^\dagger_{-\boldsymbol{k}\uparrow j}]^T$. Using the above Hamiltonian we can compute the expectation values for the self-consistency equations

$$\langle\psi_{-\boldsymbol{p}\downarrow j}\,\psi_{\boldsymbol{p}\uparrow j}\rangle = -\frac{\Delta^{(s)}_j(\boldsymbol{k}) + \Delta^{(a)}_j(\boldsymbol{k})}{\sqrt{\zeta^2_{\boldsymbol{k}j} + \left[\Delta^{(s)}_j(\boldsymbol{k}) + \Delta^{(a)}_j(\boldsymbol{k})\right]^2}} \tag{12}$$

$$\langle\psi_{-\boldsymbol{p}\uparrow j}\,\psi_{\boldsymbol{p}\downarrow j}\rangle = -\frac{\Delta^{(s)}_j(\boldsymbol{k}) - \Delta^{(a)}_j(\boldsymbol{k})}{\sqrt{\zeta^2_{\boldsymbol{k}j} + \left[\Delta^{(s)}_j(\boldsymbol{k}) - \Delta^{(a)}_j(\boldsymbol{k})\right]^2}} \tag{13}$$

C. Results

In what follows we describe the results of our simulation. We first review the properties of the normal state. We then turn to the gap structure and finally use it to compute the tunneling DOS.

We show that under the right circumstances the isolated monolayer ($t_\perp = 0$) can develop huge anisotropy in momentum space, resulting from singlet-triplet mixing, which in turn, leads to strong "multi-gap" features in the

tunneling density of states. The anisotropy and singlet-triplet mixing diminish upon increasing the interlayer coupling $t_\perp$, which is consistent with the body of experimental work.

Two ingredients are essential for this behavior to appear: (i) The attractive interaction needs to be of finite range. In particular, the on-site attraction needs to be comparable, or smaller than, the nearest neighbor and next-to-nearest neighbor interaction. This may occur due to repulsive Coulomb interaction, which tends to be shorter ranged than the phonon attraction. (ii) Breaking of inversion symmetry, which in this case is manifested by the Ising spin-orbit coupling. Without, the breaking of inversion the singlet-triplet mixing is prohibited and the effect disappears.

1. Dispersion

In Supplementary Figures 11, 12,13 we plot the dispersion functions of the model Hamiltonian Eq. (9), $\zeta_\pm$ as a function of momentum for the case of $t = 0.25$ eV, $\lambda = 0.3$ eV and $t_\perp = 0$, 0.25λ & 0.75λ on the left. The corresponding Fermi surfaces in the 1$^{\rm st}$BZ in the middle panels and the single particle density of states as a function of energy in the right panels. As can be seen, the model qualitatively mimcs the features of the band structure in H structured TMD metals.

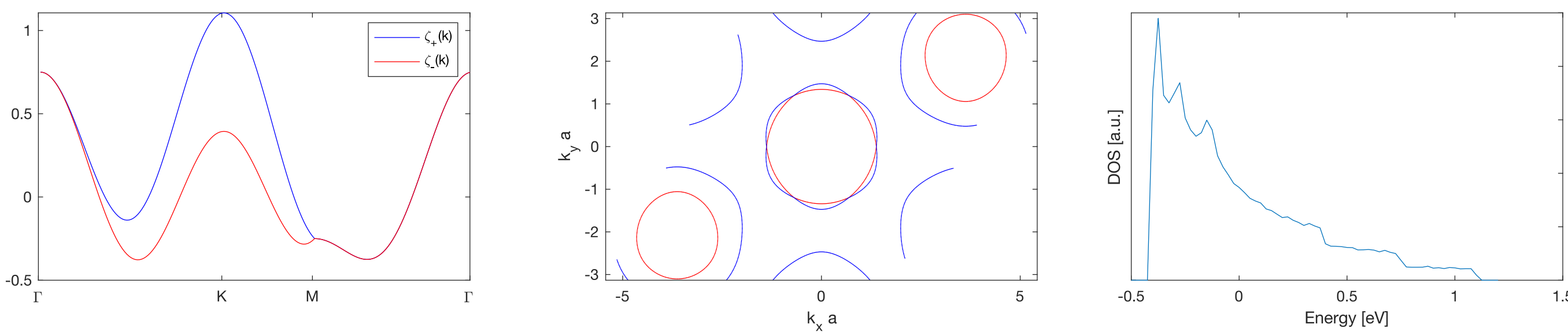

Supplementary Figure 11: **Dispersion, Fermi surface and DOS of t=0**. Left: The dispersion functions of the model Hamiltonian Eq. (9), $\zeta_\pm$ as a function of momentum for the case of $t = 0.25$ eV, $\lambda = 0.3$ eV and $t_\perp = 0$. Middle: The corresponding Fermi surfaces in the 1$^{\rm st}$BZ. Right: The single particle density of states as a function of energy.

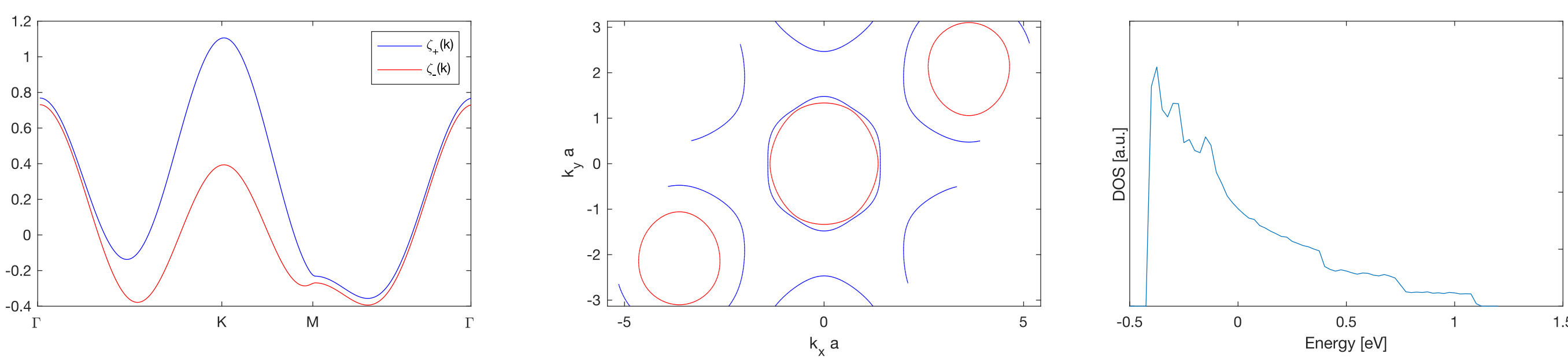

Supplementary Figure 12: **Dispersion, Fermi surface and DOS of t=0.25**. Left: The dispersion functions of the model Hamiltonian Eq. (9), $\zeta_\pm$ as a function of momentum for the case of $t = 0.25$ eV, $\lambda = 0.3$ eV and $t_\perp = 0.25\lambda$. Middle: The corresponding Fermi surfaces in the 1$^{\rm st}$BZ. Right: The single particle density of states as a function of energy.

2. Gap structure and Tunneling density of states

In Supplementary Figures 14, 15 and 16 we plot the results of a self-consistent calculation of Eq. (10) over the entire Brillouin zone. All figures contain the four gap functions in the Hamiltonian Eq. (11). Then the last two panels are the tunneling density of states and the corresponding band structure.

For all three cases we use a finite range interaction $V_0 = 0.25t$ and $V_1 = V_2 = 0.3t$, where we assume the short range attraction is diminished due to Coulomb repulsion. For the momentum space integrals we use a Monkhorst-Pack Grid

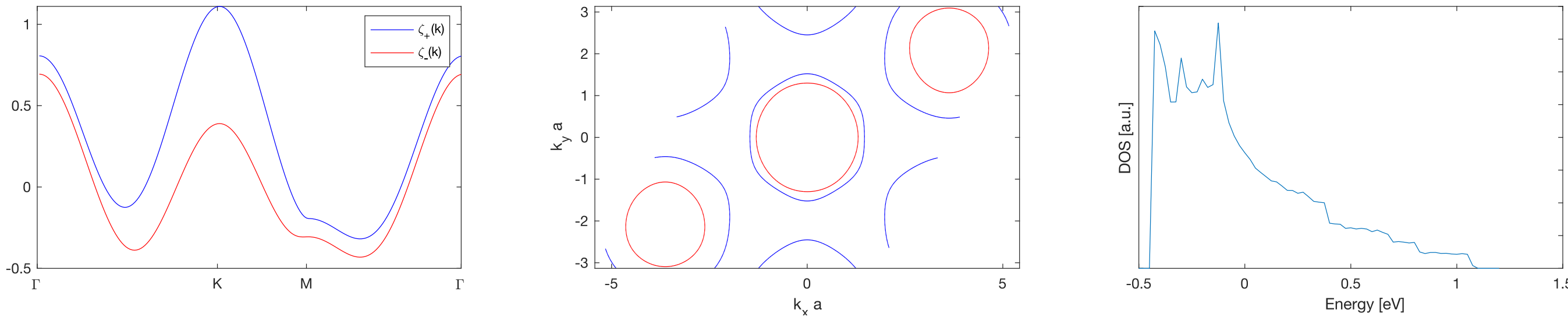

Supplementary Figure 13: Dispersion, Fermi surface and DOS of t=0.75. Left: The dispersion functions of the model Hamiltonian Eq. (9), $\zeta_{\pm}$ as a function of momentum for the case of $t = 0.25$ eV, $\lambda = 0.3$ eV and $t_{\perp} = 0.75\lambda$. Middle: The corresponding Fermi surfaces in the 1^{st}BZ. Right: The single particle density of states as a function of energy.

with 40 points in each direction. For the tunneling density of states calculation we use an Allen-Dynes broadening parameter $\Gamma = 1$ meV.

Supplementary Figure 14 shows the case of two isolated H layers $t_{\perp} = 0$. Here we find a large singlet-triplet mixing. The ratio between the small and big gap on the small K and K' pockets is of the order 0.1. Consequently, we clearly see two gaps in the tunneling density of states.

Supplementary Figure 15 shows the case of two isolated H layers $t_{\perp} = 0.1$ eV. We observe that the increase of the singlet-triplet mixing has diminished due to the increasing $t_{\perp}$, as reflected by the by the weaker anisotropy in the sizes of the gaps across the BZ.

Finally, in Supplementary Figure 16 we show the case of two isolated H layers $t_{\perp} = 0.2$ eV. We observe that the second gap feature in the tunneling density of states has disappeared due to the decreasing singlet-triplet mixing.

3. Conclusions

We have demonstrated how a multi-gap feature can appear in the Bogoliubov spectrum due to singlet-triplet mixing in a single layer of 2H-TaS_2. Furthermore, we have shown how the interlayer coupling between the two constituate layers suppresses the mixing and by that the multi-gap spectral signiture.

Here it is important to make a comment. First, in principle, a superposition of the gap functions 1 and $e(\boldsymbol{k})$ can also lead to a variation of the gap (between the K/K' pockets and the Γ pocket). However, unlike in the singlet-triplet mixing scenario, this type of mixing is not expected to depend strongly on interlayer coupling.

---

* These authors contributed equally

[1] Gunnarsson, O., Calandra, M. & Han, J. Colloquium: Saturation of electrical resistivity. *Reviews of Modern Physics* **75**, 1085 (2003).
[2] Almoalem, A. *et al.* Charge transfer and spin-valley locking in 4hb-tas2. *npj Quantum Materials* **9** (2024). URL https://www.nature.com/articles/s41535-024-00646-2.
[3] Almoalem, A. *et al.* Link between superconductivity and a lifshitz transition in intercalated bi 2 se 3. *Physical Review B* **103**, 174518 (2021).
[4] Bawden, L. *et al.* Spin-valley locking in the normal state of a transition-metal dichalcogenide superconductor. *Nature Communications* **7**, 11711 (2016). URL https://www.nature.com/articles/ncomms11711.
[5] Fang, S. *et al.* Ab initio tight-binding hamiltonian for transition metal dichalcogenides. *Physical Review B* **92**, 205108 (2015).
[6] Sergio C. de, l. B. *et al.* Tuning ising superconductivity with layer and spin-orbit coupling in two-dimensional transition-metal dichalcogenides. *Nature Communications* **9** (2018). URL https://www.nature.com/articles/s41467-018-03888-4.
[7] Vano, V. *et al.* Evidence of nodal superconductivity in monolayer 1h-tas2 with hidden order fluctuations. *Advanced Materials* **35**, 2305409 (2023).

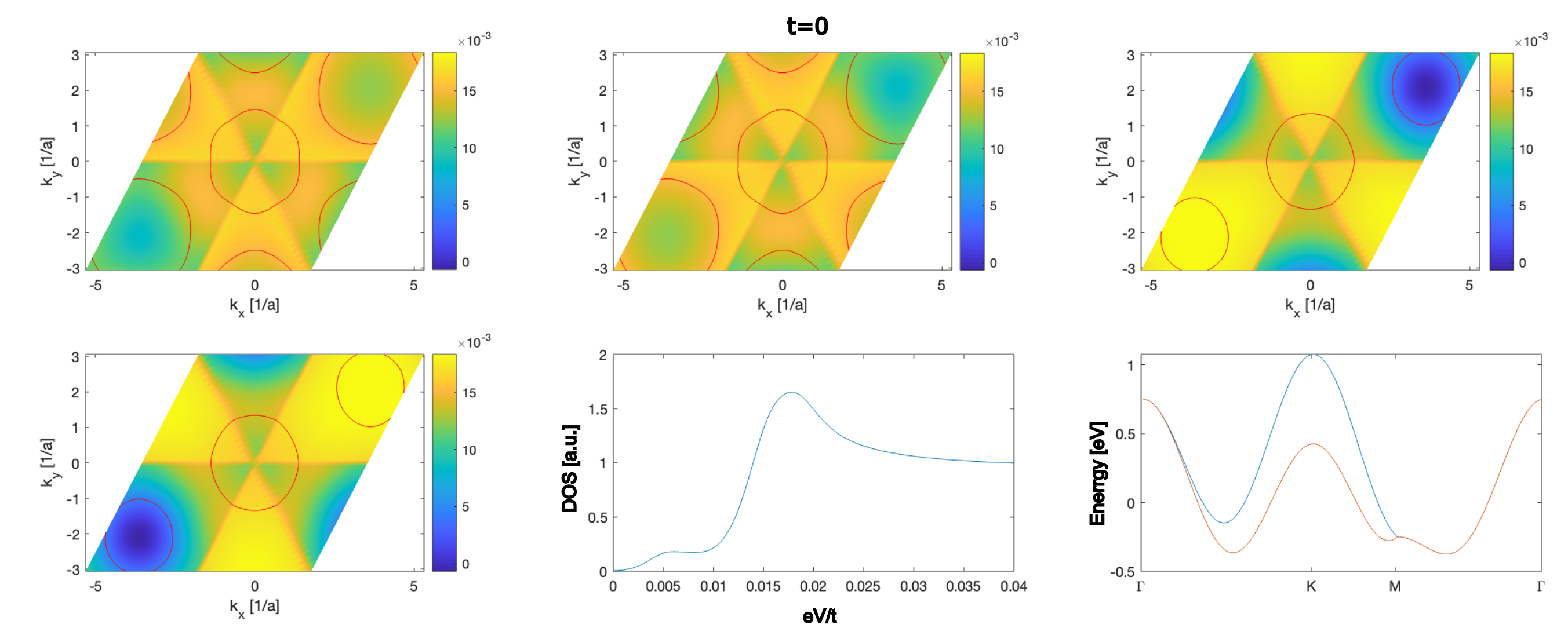

Supplementary Figure 14: **Superconducting gap structure in the Fermi surface for t=0**. The three top panels and the lower left panel are a plot of the gap function in the first Brillouin zone for $t_\perp = 0$, $\lambda = 0.3$ eV, $t = 0.25$ eV, $V_0 = 0.25t$, $V_1 = V_2 = 0.3t$. From top left, $\Delta_+^{(s)} + \Delta_+^{(a)}$, $\Delta_+^{(s)} - \Delta_+^{(a)}$, $\Delta_-^{(s)} + \Delta_-^{(a)}$ and $\Delta_-^{(s)} - \Delta_-^{(a)}$, respectively. The remaining two plots are the corresponding tunneling density of states and band-structure.

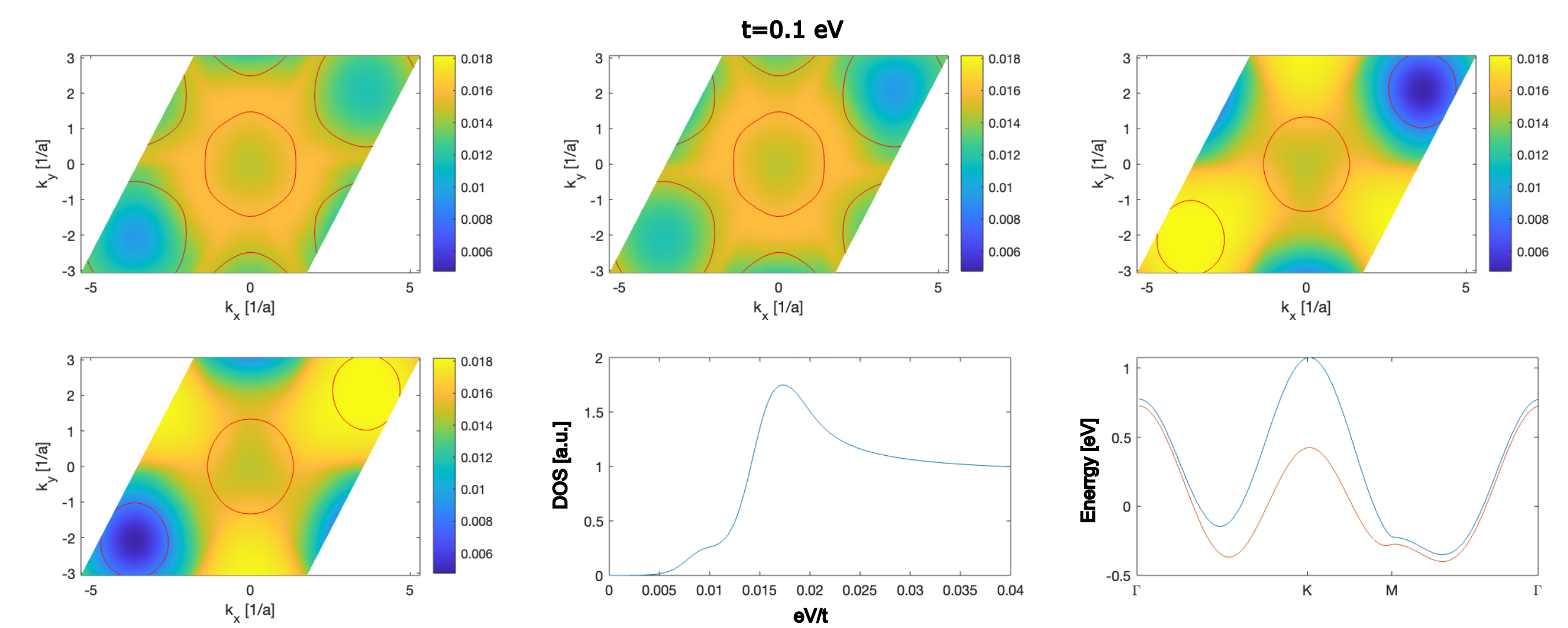

Supplementary Figure 15: **Superconducting gap structure in the Fermi surface for t=0.1**. The three top panels and the lower left panel are a plot of the gap function in the first Brillouin zone for $t_\perp = 0.1$ eV, $\lambda = 0.3$ eV, $t = 0.25$ eV, $V_0 = 0.25t$, $V_1 = V_2 = 0.3t$. From top left, $\Delta_+^{(s)} + \Delta_+^{(a)}$, $\Delta_+^{(s)} - \Delta_+^{(a)}$, $\Delta_-^{(s)} + \Delta_-^{(a)}$ and $\Delta_-^{(s)} - \Delta_-^{(a)}$, respectively. The remaining two plots are the corresponding tunneling density of states and band-structure.

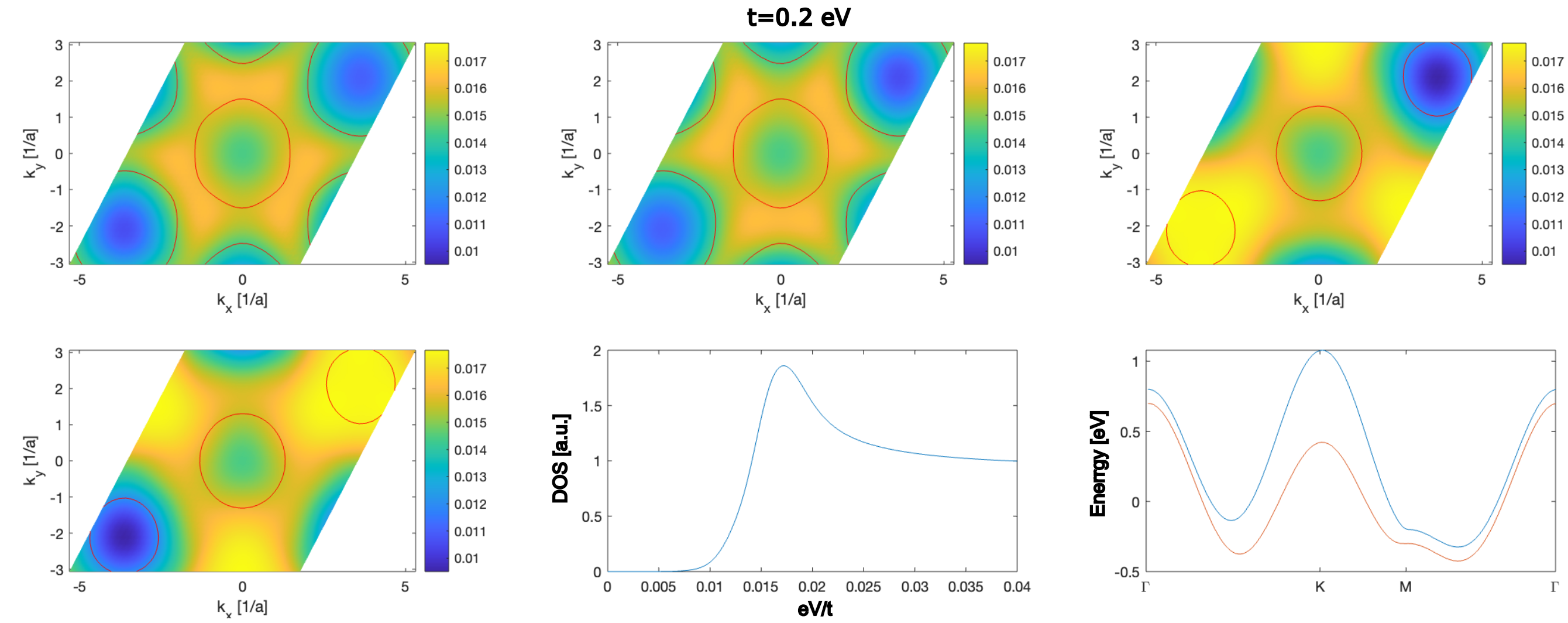

Supplementary Figure 16: **Superconducting gap structure in the Fermi surface for t=0.2**. The three top panels and the lower left panel are a plot of the gap function in the first Brillouin zone for $t_\perp = 0.2$ eV, $\lambda = 0.3$ eV, $t = 0.25$ eV, $V_0 = 0.25t$, $V_1 = V_2 = 0.3t$. From top left, $\Delta_+^{(s)} + \Delta_+^{(a)}$, $\Delta_+^{(s)} - \Delta_+^{(a)}$, $\Delta_-^{(s)} + \Delta_-^{(a)}$ and $\Delta_-^{(s)} - \Delta_-^{(a)}$, respectively. The remaining two plots are the corresponding tunneling density of states and band-structure.
