# Supplementary material for: Mixed triplet-singlet order parameter in decoupled superconducting 1H monolayers of transition-metal dichalcogenides
Source: Nat Commun. 2026 Jul 16;17:8718. doi: 10.1038/s41467-026-75677-3 (PMC13490457; doi:10.1038/s41467-026-75677-3)
Supplement: Supplementary file 2 [file 41467_2026_75677_MOESM2_ESM.pdf]

nature portfolio

Peer Review File

Mixed Triplet-Singlet Order Parameter in Decoupled Superconducting 1H Monolayers of Transition-Metal Dichalcogenides

Corresponding Author: Dr Avior Almoalem

This file contains all reviewer reports in order by version, followed by all author rebuttals in order by version.

Attachments originally included by the reviewers as part of their assessment can be found at the end of this file.

Version 0:

Reviewer comments:

Reviewer #1

(Remarks to the Author)
In the manuscript by Almoalem and colleagues, the authors studied the misfit compound (SnS)1.15(TaS2) utilizing transport, photoemission, scanning tunneling microscopy with theoretical modeling. Their transport results reveal the emergence of superconductivity around 3.1 K. Spin-resolved angle-resolved photoemission spectroscopy (ARPES) identifies spin-momentum locking, which could provide additional evidence for Ising superconductivity. Low-temperature scanning tunneling microscopy results show a two-gap superconducting behavior in the TaS2 layer with an unexpected single superconducting gap in the SnS layer. Theoretical analysis based on a minimal tight-binding model with inter-layer coupling, Ising spin-orbit coupling, and pairing interaction reproduces two-gap superconductivity when the inter-layer coupling is reduced as observed in experiments.
While the authors' clear presentation of superconducting gap is impressive, the key findings of this manuscript seem incremental. Similar and more obvious experimental consequences from inter-layer decoupling have been already reported elsewhere (for example, 4Hb-TaS2 in Ref. 53 or monolayer 1H-TaS2 in Ref. 48). It would be more reasonable using those systems as a platform to suppress inter-layer interaction in TaS2 rather than utilizing SnS layer. Unless the authors can provide and explain experimental discovery that distinguishes (SnS)1.15(TaS2) from other polymorphs of TaS2, I cannot recommend this paper for publication in Nature Communications.

Additional comments:
1. Although the authors propose that internal strain applied to the SnS layer could introduce superconductivity, it remains unclear how a simple structural arrangement can result in such a large pressure (~ 40 GPa). This is important as the authors rule out superconducting proximity effect from potential origins.
2. Typo in Page 15 line 274: "strongly coupled case" might be corrected with "decoupled case".
3. Experimental details of the ARPES experiments should be clearly described in the manuscript. In particular, the experimental geometry is a key factor for analyzing spin-resolved ARPES results meanwhile such information is missed in the manuscript. Lastly, the quality of presented spectra is noticeably lower than that of other recent studies.

Reviewer #2

(Remarks to the Author)
In this work, the authors study the misfit compound SnS-TaS2, made of quasi-isolated layers of insulator SnS and superconductor TaS2. The authors provide a range of experiments including transport, spin resolved ARPES and STM. The novel contribution is the observation of a two gap structure in STM, which the authors explain in terms of singlet-triplet mixing, which provides different gaps for the spin split pockets at the K point.

I think the approach of using misfits to study 2D SC is very interesting, and the two-gap structure in STM is new for such monolayers (not so much the rest of experiments). I also think it is interesting to perform these measurements to compare with MBE grown monolayers which have shown unexpected behavior. However, I also think the provided theoretical explanation is not the only possible one, and the paper has other drawbacks, so I am not very convinced that the current

version belongs to Nat. Comm.

- First, a main drawback of the current manuscript is that it does not provide a reasonable account of previous research in the misfit compounds. It is not really a novel finding of this manuscript that the spacer layers decouple the H layers and make 2D superconductivity (while the text states “our results… suggest that the SnS layers act as a buffer layer…”). Specifically for SnS-TaS2 misfit, Sankar et al (Chem. Mater. 2018, 30, 4, 1373–1378) reported 2D SC with Tc=3 K. Bai et al (2018 J. Phys.: Condens. Matter 30 355701) reported multiband superconductivity from transport in the SnSe-NbSe2 misfit. A very detailed study on this compound which includes transport, STM and ARPES is only cited in passing as a “high-temperature study” (for STM), but it did observe SC at 3 K too. And finally, a paper with authors in common just published in 2025 (PRB 111, 054509 (2025)) observed many similar features in PbSe-TaS2 except the tunneling experiments. The authors should discuss the previous literature and emphasize the novelty of their results compared to these.

- I appreciate the authors’ effort in comparing with the multigap state observed in Ref. 35, which remains a puzzling and important result in the field. However, the authors should state that the coherence peaks of the smaller gap in that work appear around 180 ueV (by eye, the authors may have the data) which is taken at 25 mK. That paper also states the smaller gap disappears at 400 mK, even though Tc is 3K. Since the STM data is taken at 300 mK, why is the gap in STM (300 ueV) larger at larger temperature? If STM is done at say 500 mK is the gap still detectable? I am not sure the gap in Ref. 35 is the same feature as the one observed here.

A main difference between these two experiments is that planar junctions enhance the visibility of small momentum states compared to point junctions (see Berthod, PRB 84, 155414 (2011)). Since monolayer TaS2 has a d-derived Gamma pocket in addition to those at K/K’, it is possible that the planar junction is observing the SC gap of this band.

- Regarding the STM double gap, it is also known in these compounds that due to the momentum dependence of electron-phonon coupling, the gap at K is strongly anisotropic (see for example ARPES in PRB 85, 224532 (2012) and PRL 119, 087004 (2017) for a DFT calculation). This could be a natural explanation for the apparent two-gap structure. Can the authors provide evidence on the contrary?

- I am also very confused by the theoretical model. Fig 1b and the text that describes the structure (“here the 1H layers maintain the same orientation, making the crystal lattice lack overall inversion symmetry”) clearly show that the stacking of the H layers breaks inversion (unlike the 2H structure or the H layers in the 4Hb structure, which alternate in orientation and have an inversion center between the layers). But the theory is built for a globally symmetric system, so it applies to 4Hb and 2H, but not to the misfit. The labeling of irreps is also confusing. The authors state that the point group is D3, which is not correct. The mentioned irreps are either those of D6h (like in 2H or 4Hb) or D3d. But the group of a monolayer is D3h, again with different irreps.

- Assuming that the right model is the one for a monolayer, there are DFT calculations of the splitting of the gap at K/K’ (see npj Comp. Mat. 9, 66 (2023)) due to Ising SC. The inner/outer gap difference is small both with phonon and spin fluctuation pairing (which might be loosely considered as originating from Coulomb interaction). The splitting obtained with the model in this work is quite large compared to the DFT calculations (which are of course not really comparable), but this raises the question of whether the model is really reasonable. Again, rather than singlet-triplet mixing, I think gap anisotropy might be a more likely explanation.

Other minor comments:

- The authors state in the introduction that SnS decouples the 1H layers and that this conclusion is evident from the sample Tc 3.1 K. However, at this point they have not mentioned the fact that Tc goes from 3 K in the monolayer to 0.7 K in bulk, so why is this “evident” it is unclear for a non-expert reader. Since the authors then say this explicitly further down in the text any way, I suggest moving that sentence upwards so that the evidence is easily understood (ideally when they cite all previous works that have already seen this).

- I am surprised by Refs 36 (about moire TMDs) and 37 which is about insulating WSe2. The sentence is about phonon softening in metallic 2H TMDs which have a CDW transition (in the bulk, all do except NbS2). The authors should provide supporting references.

- Regarding unconventional pairing in NbSe2 I believe Cho et al, PRL 129, 087002 (2022) is as relevant as Ref 31, the authors may consider citing it.

- The title of Ref. 53 made the claim of an f-wave OP, but I encourage the authors to critically evaluate if the reported evidence poses any constraints on the symmetry of the order parameter. I think it is best to refer to this as a V shape gap.

Reviewer #3

(Remarks to the Author)
The manuscript investigates unconventional superconductivity in the misfit compound (SnS)1.15(TaS2), composed of alternating SnS and 1H-TaS2 layers. By combining transport, ARPES, and STM spectroscopy, the authors suggest that the SnS layers electronically decouple the TaS2 sheets, leading to quasi-two-dimensional superconductivity with a clear two-gap feature. A theoretical model incorporating broken inversion symmetry and finite-range pairing interactions reproduces

the observed mixed singlet-triplet character. Overall, the work provides new insights into how unconventional pairing emerges in isolated 1H-TaS_2 layers and highlights misfit compounds as a promising platform for exploring multi-gap and parity-mixed superconductivity. However, several aspects of the study remain insufficiently addressed, which currently limits the clarity and rigor of the manuscript. I am unable to recommend the manuscript for publication in Nature Communications at this stage. However, it may become suitable once the authors have carefully addressed the points detailed below.

(1)Figures 2 and 3 present the transport and ARPES measurements. However, since these are non-local techniques and the SnS layer is also reported to be superconducting, it is crucial to clarify the role of the SnS layer. The discussion of this aspect is currently insufficient and somewhat unclear. For instance, the authors state in the main text (lines 159-163) that the coherence length extracted from the vortex agrees with that estimated from the upper critical field. Yet, in Supplementary Material V, the vortex is shown in the SnS layer, and no data are provided for the vortex in the 1H-TaS_2 layer. This inconsistency is confusing and undermines the logical coherence of the related discussion. Considering that a two-gap model was used in Fig. 2b to fit the evolution of the upper critical field, it appears that the authors attribute the transport and ARPES results solely to the TaS_2 layer, while the SnS layer is only included later in the self-consistent analysis (e.g., the discussion of the coherence length). I believe the authors need to provide clarification on these issues.
(2)The main text (lines 120-121) refers to Fig. 2c, but such a panel is not included in the currently presented Figure 2.
(3)In a previous work (Adv. Mater. 2023, 35, 2305409), nodal Superconductivity has been reported in the pristine monolayer 1H-TaS_2. I believe the authors should discuss the origin of this discrepancy about the gap features in the manuscript.
(4)As suggested by the theoretical model presented in the manuscript, the superconducting state without interlayer coupling appears weaker than that with interlayer coupling, which contrasts with the experimental findings. It would be helpful if the authors could further discuss this discrepancy.
(5)Given that multiple mechanisms can give rise to two-gap superconductivity, the authors have not provided sufficient justification to exclude alternatives other than the mixed singlet-triplet pairing scenario. In Supplementary Material VI, ARPES data are presented to demonstrate a vanishing interlayer coupling between the 1H-TaS_2 layers; however, this evidence appears insufficient. Although the manuscript states that the superconductivity in SnS does not originate from the proximity effect of TaS_2, it remains important to further clarify whether any coupling exists between the superconducting states of the two layers, and how such coupling, if present, might influence the main conclusions.

Reviewer #4

(Remarks to the Author)
I co-reviewed this manuscript with one of the reviewers who provided the listed reports. This is part of the Nature Communications initiative to facilitate training in peer review and to provide appropriate recognition for Early Career Researchers who co-review manuscripts.

Version 1:

Reviewer comments:

Reviewer #1

(Remarks to the Author)
I appreciate the authors' effort in revising the manuscript, supplementary material, and discussions therein. Now the manuscript is improved, especially in the ARPES method and in the broader literature discussion. Following the authors' argument, the key finding of this work is the mixed order parameter due to the absence of inter-layer coupling. They also argued that the proximity effect from SnS layers must be irrelevant to their observation of two-gap superconductivity. However, I still cannot recommend the work for publication in Nature Communications since my main concerns raised in the previous review—that the key findings seem incremental, and the support is insufficient—have not been resolved.
First, this work is not the first one reporting the vanishing of interlayer interaction in a misfit compound. Some of the authors published a paper on evidence of vanishing of interlayer interaction in $(PbS)_{1.13}(TaS_2)$ [Ref. 60 in the main text] and suggested potential two-gap superconductivity from critical field measurements. In addition, there are also concerns on the claim itself. In the Supplementary Material, the authors put together additional ARPES data on examination of kz-dispersion along the M-L high symmetry points. Though the energy feature at -0.325 eV (the description in line 167 saying -0.325 meV seems like a typo) is dispersionless along the kz axis at zone boundary, it does not explicitly confirm that only the charge transfer between the SnS and TaS_2 layers is relevant near EF. Such a characteristic would require further verification from termination dependence of ARPES spectra as well as core level examinations.
Secondly, 4Hb-TaS_2 presents a benchmark for the authors' theoretical framework, meanwhile they make contradictory statements regarding the two-gap behavior in it. According to the authors' response to Reviewer #2, 4Hb-TaS_2 must match well with the theoretical model in the main text as it is inversion-symmetric with suppressed interlayer interaction. If interlayer decoupling alone without inversion symmetry breaking were sufficient to produce two-gap features, one would expect 4Hb-TaS_2 to exhibit similar spectroscopic signatures to $(SnS)_{1.15}TaS_2$.—yet no STM study on 4Hb-TaS_2 has reported two distinct coherence peak energies as claimed in the present work. In addition, authors' explanation with impurities remains speculative.
Lastly (and on minor notes), the manuscript still needs improvement in writing as it still has technical errors in several places.
In line 61, "mono-chalgoen" must be "mono-chalcogen".
In line 148, Fig. 2c does not exist while the sentence refers to it. (Reviewer #3 already pointed this out in their previous comment.)
In line 191 and 192, "wether" must be "whether".

In line 301, “the where inversion” may be “where the inversion”.
In line 360, “1d” may be “as shown in Fig. 1d”.

Reviewer #2

(Remarks to the Author)
I thank the authors for engaging in constructive discussion and for their detailed answers to all of my points.

I am satisfied with the description of previous literature, and with the explanation about the comparison with Ref. 35, where now I see it is plausible that the experiments are compatible. I think it would be useful to write explicitly in the text that describes Fig 4 that Delta_1/2 = Delta_s \pm \Delta_f and the values (250 and 530) for the two coherence peaks.

Regarding alternative explanations of the gap splitting, it is also true that if a momentum dependent gap were the explanation, then some signature of this would presumably be seen in the bulk too, although the dependence of gap anisotropy in number of layers has never really been calculated. Nevertheless, the authors have explained their point of view clearly and compared with alternative explanations so I think this is sufficient.

I also understand better the logic of the theoretical model. The authors are not trying to make a model for the misfit compound, but rather to show the generic point about how interlayer coupling washes out the singlet-triplet splitting in centrosymmetric structures. I also see now the symmetry discussion was inspired by Ref. 78 which incorrectly assigned D3d symmetry to SrPtAs, an error that appears to have propagated through the literature but was later fixed in Fischer, JPSJ 84, 054705 (2015). The space group of SrPtAs is 194 (D6h) and the analysis is therefore the same as in TMDs. Incidentally, the f-wave functon(with opposite sign in K and K’, i.e. odd under the mirror sigma_v, but even under the mirror sigma_h) is not A2u but B2u in D6h. The text claims the function with A2u symmetry is even under the mirror line Gamma-M, but the function drawn on the lower part of Fig. 5a is odd (as it should to have opposite sign on K and K’).

To be more precise, the pairing function in Ref. 78 has three pieces: the momentum form factor, the layer matrix, and the spin factor. For the centrosymmetric f-wave triplet, the momentum form factor is B2u, the z-spin triplet is A2g, and the pairing is opposite in the two layers (a B1u function). The product of these three gives A1g and is therefore allowed to mix in with the standard s-wave. In simple terms, the f-wave triplet pairing is allowed in centrosymmetric bilayers if it takes opposite signs in the two layers. This triplet piece is disfavored by interlayer coupling, which is the point of the calculation.

In summary, I only think the symmetry analysis needs a more careful look but I otherwise I am happy to recommend publication in NC.

Reviewer #3

(Remarks to the Author)

Reviewer #4

(Remarks to the Author)
I co-reviewed this manuscript with one of the reviewers who provided the listed reports. This is part of the Nature Communications initiative to facilitate training in peer review and to provide appropriate recognition for Early Career Researchers who co-review manuscripts.

Version 2:

Reviewer comments:

Reviewer #1

(Remarks to the Author)
I thank the authors for their detailed point-by-point response and for the further revisions to the manuscript and Supplementary material. Several of my concerns have been addressed. I would like to ask the authors to consider bringing the justification for using the misfit layer for their study in a more explicit way. Although the advantages of using the misfit layer could be found in a couple of sentences in the main text, it would be better to bring this point to the front as it is one of the key ideas of the manuscript, basically putting the scattered arguments into one or two places in the introduction part.
Minor notes: In the caption for Fig. 3, “Fig. 1c.” and “1e” may be “1d” and “1f”, respectively. In addition, Refs. [77] and [84] are the same paper.

Reviewer #2

(Remarks to the Author)
I thank again the authors for engaging in the discussion, and with the latest changes I support publication in NC.

Reviewer #3

(Remarks to the Author)
I co-reviewed this manuscript with one of the reviewers who provided the listed reports. This is part of the Nature Communications initiative to facilitate training in peer review and to provide appropriate recognition for Early Career Researchers who co-review manuscripts.

Reviewer #4

(Remarks to the Author)
I co-reviewed this manuscript with one of the reviewers who provided the listed reports. This is part of the Nature Communications initiative to facilitate training in peer review and to provide appropriate recognition for Early Career Researchers who co-review manuscripts.

Open Access This Peer Review File is licensed under a Creative Commons Attribution 4.0 International License, which permits use, sharing, adaptation, distribution and reproduction in any medium or format, as long as you give appropriate credit to the original author(s) and the source, provide a link to the Creative Commons license, and indicate if changes were made.
In cases where reviewers are anonymous, credit should be given to 'Anonymous Referee' and the source.
The images or other third party material in this Peer Review File are included in the article's Creative Commons license, unless indicated otherwise in a credit line to the material. If material is not included in the article's Creative Commons license and your intended use is not permitted by statutory regulation or exceeds the permitted use, you will need to obtain permission directly from the copyright holder.
To view a copy of this license, visit https://creativecommons.org/licenses/by/4.0/

REVIEWER COMMENTS

We thank the referees for their careful review of our work. We appreciate their assessment, particularly their recognition of the interest and high quality of our work. At the same time, the referees have raised questions that they would like addressed before recommending publication in *Nature Communications,* mainly concerning our theoretical model. In response, we have revised the Theoretical and Discussion sections to improve the clarity of the paper in accordance with their comments. We also added more supplementary figures, and higher quality ARPES data. Below, we provide a point-by-point reply to all referee remarks.

We mark changes in the manuscript and supplementary data in red color.

Reviewer #1 (Remarks to the Author):

the key findings of this manuscript seem incremental. Similar and more obvious experimental consequences from inter-layer decoupling have been already reported elsewhere (for example, 4Hb-TaS2 in Ref. 53 or monolayer 1H-TaS2 in Ref. 48). It would be more reasonable using those systems as a platform to suppress inter-layer interaction in TaS2 rather than utilizing SnS layer. Unless the authors can provide and explain experimental discovery that distinguishes (SnS)1.15(TaS2) from other polymorphs of TaS2, I cannot recommend this paper for publication in Nature Communications.

We thank the referee for their comment. Below we highlight the differences between our misfit SnS-TaS_2 system and the work in references 53 and 48.

4Hb-TaS2 in Ref. 53 and 49

First, we note that the material properties of **4Hb-TaS2** are quite different from the misfits. **4Hb-TaS2 has a** depleted Mott layer in proximity with the 1H layer which the misfits don't have. **ARPES** data of SnS-TaS2 near the Fermi level show a typical Fermi surface of the 1H compounds with no additional Fermi pockets, as seen here in our reply. Contrary to that, a modified Fermi surface exists in the 4Hb-TaS2, where additional pockets were seen [Science advances 6, eaax9480 (2020), npj Quantum Materials 9 (2024)]. **STM** spectra acquired on the SnS layer show no significant modification from the expected semiconducting spectra, as opposed to the spectra acquired on the 1T layer of 4Hb-TaS2, in which renormalization of the band structure was measured using STM [Proceedings of the National Academy of Sciences 120, e2304274120 (2023)]. Equally important is the fact that **4Hb-TaS2 preserve global inversion symmetry** with a centrosymmetric unit cell, although each 1H layer is non-centrosymmetric by itself. Contrary to that, **SnS-TaS2 lack inversion symmetry**, with no inversion center for the overall unit cell and the 1H layers.

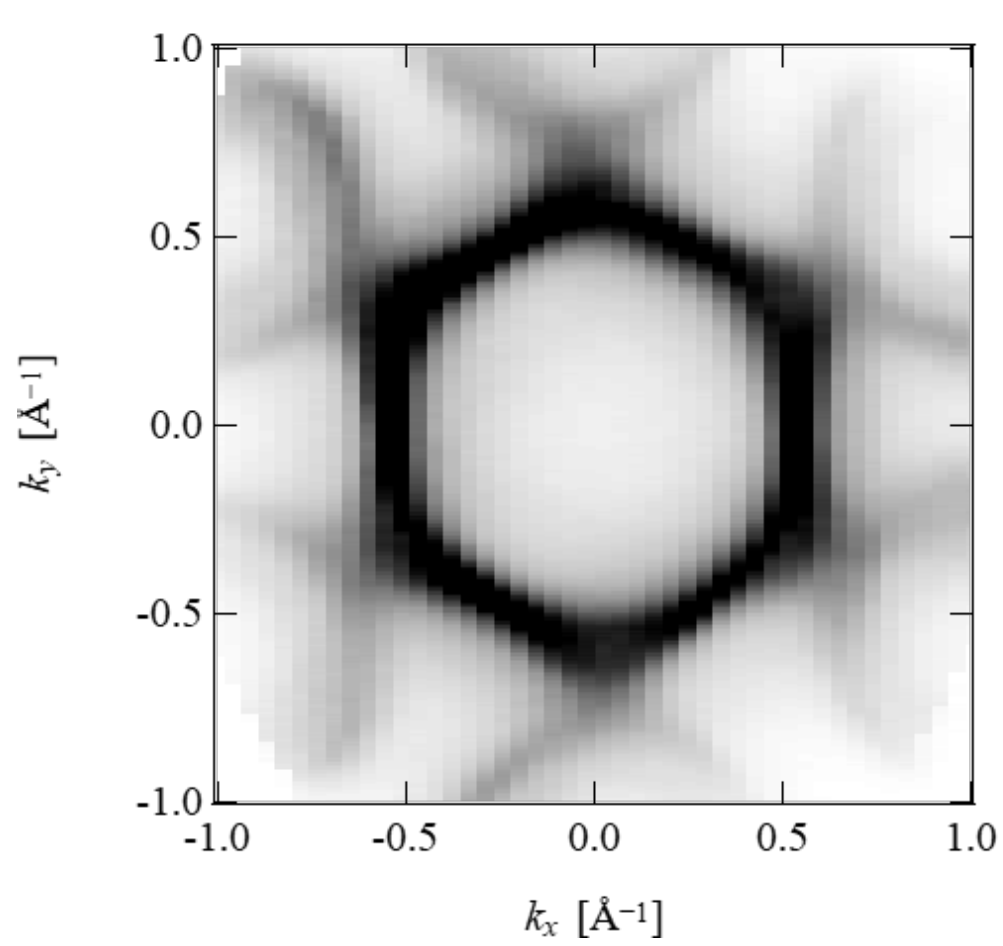

It is therefore not surprising that superconductivity in **4Hb-TaS2** is also different compared to what is observed in this study**.** While **4Hb-TaS2** was shown to have a two-component order parameter

(reference 53 for example) STM spectroscopy shows only one gap. Other studies of 4Hb-TaS_2 have reported characteristics such as edge states, as in reference 49 which suggest that it might be a topological superconductor; broken time reversal symmetry in the superconducting phase; the existence of a gap-less superconducting phase; and a spontaneous memory effect in the superconducting state. In contrast we find no edge states in edges of the 1H layers in the superconducting phase of SnS-TaS2 (see Supplementary Figure S13 in the revised manuscript), and no sign of broken time reversal symmetry in SnS-TaS_2.

From a spectroscopic point of view, the two systems also exhibit significant differences both in the gap structure and the Fermi-level DOS. As mentioned before, there is no sign of a second gap or gap-anisotropy in **4Hb-TaS2. Also,** while the 1H layer in the 4Hb has a residual density of states, the 1H layer in SnS-TaS2 shows a complete gap. Finally, in the SnS layer we find a robust s-wave gap, contrary to the 1T layer in 4Hb-TaS2 where such a gap is absent.

Finally, we note that all studies done on 4Hb-TaS2 do not attribute any of the findings to the elimination of interlayer coupling. Instead, in reference 49 the theoretical model claims that the properties measured are due to charge transfer between the layers.

1H-TaS2 in Ref. 48:

The monolayer 1H TaS_2 samples **Ref. 48** show a large gap in the dirty areas and a smaller nodal gap in ultraclean areas. Since the s-wave and nodal gaps exist in different areas with different disorder, it was suggested that disorder quenches the f-wave channel (nodal gap), thus giving rise to the s-wave gap. This scenario is completely different from our findings in which both s and f-wave coexist simultaneously.

Moreover, the material itself is most likely very different. Past studies have shown that the environment of the 1H layer strongly influences the nature of superconductivity. Considering the specific case of the monolayer 1H MBE grown sample, [Advanced Materials 35, 2305409 (2023)] the film was grown on HOPG with a lattice parameter around 2.4 Å, while 1H-TaS2 has a lattice spacing of 3.3 Å. This likely creates strain on monolayer 1H-TaS2. Additionally, the description of the samples as having a “low defect density” suggests that residual defects are still present, potentially affecting the fragile superconducting behavior of the 1H-TaS_2 layer. This is also evident by the very low Tc of their sample (Tc~1K) opposed to the known Tc of monolayer TaS_2 of ~3K, which we might add is similar to Tc of our sample.

In summary, in our study we present a mixed order parameter of both s and f wave. Our calculations indicate that this mixed state arises from the vanishing interlayer coupling. In contrary, in the previous study (ref. 48) the superconducting gap evolves from nodal to s-wave, depending on the disorder level. As per their paper, this has nothing to do with interlayer coupling and instead is due to disorder. Thus, our experimental findings as well as our theory are completely different from this paper*.* In fact, our study presents the SnS-TaS2 as the materialization of a system with a non-s-wave order parameter.

Additional comments:

1. Although the authors propose that internal strain applied to the SnS layer could introduce superconductivity, it remains unclear how a simple structural arrangement can

result in such a large pressure (~ 40 GPa). This is important as the authors rule out superconducting proximity effect from potential origins. ->

We thank the referee for this question. The referee is absolutely correct that we cannot have strain equivalent to 40Gpa.

Indeed, the most important parameter that determines whether superconductivity emerges is finite density of states at the Fermi energy. This was exemplified in the pressure paper (references 93 and 94) which demonstrate that the SnS can become superconducting as soon as it acquires some DOS in the Fermi level. As written in that paper: "...By increasing the DOS near the Fermi level in SnS using band-structure engineering techniques such as chemical substitution, defect control and electric field-effect, enhancement of Tc can be expected as future work."

In our case, the existence of finite DOS at the Fermi level is directly confirmed by our STM spectra. Its origin is most likely due to charge transfer mechanism as evident from our ARPES data and disorder in the SnS layer as seen in our topography in Figure 1. It is certainly reasonable that the charge transfer alone could be sufficient to generate a finite DOS in the Fermi level of the SnS layer. To clarify this point we added clarifications in line 161 regarding the ARPES data and charge transfer mechanism, and line 375 of the discussion.

In addition, we do have strain in the sample as seen in large-scale topographies which reveal strain in the form of extended lines appearing on the surface of the 1H layer. This strain may also add to the excess density of states. Therefore, whether the emergence of superconductivity is due to interlayer pressure, structural deformation or electric field effect, is difficult to pin down but once a finite DOS emerges in the SnS layer, the material becomes superconducting as predicted in previous theoretical and experimental studies.

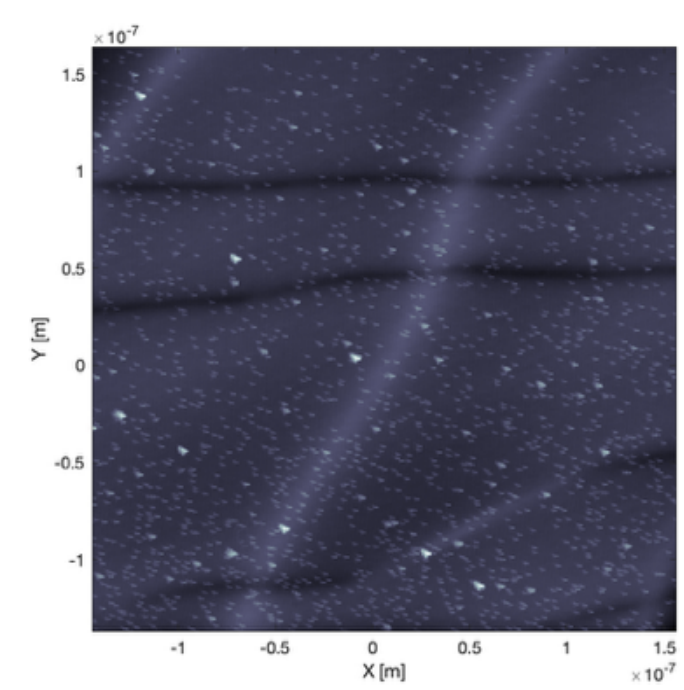

We have now changed the discussion to reflect these nuances.

Regarding the possibility that the superconducting gap in the SnS layer arises from a proximity effect, we rule out such a scenario using Hc measurements as shown in Supp. Figure 4. There we show the filling of the gap is much more pronounced in the 1H layer, while a deeper gap remains clearly visible in the SnS layer. This indicates that the gap is independent of the 1H layer, and cannot be of proximity origin, since a proximity-induced gap would be expected to be smaller and more fragile than the gap of the parent superconductor.

Finally, from the fitting analysis, the gaps in the 1H layer have magnitudes of 390 and 140 μeV, whereas the SnS layer exhibits a single gap of 440 μeV. The SnS layer is thus clearly characterized by a fully gapped s-wave gap that is larger than those in the 1H layer, further ruling out a proximity-induced origin.

This discussion has also been added to the manuscript.

2. Typo in Page 15 line 274: "strongly coupled case" might be corrected with "decoupled case".

We thank the referee for noticing it. We fixed the typo as pointed out.

3. Experimental details of the ARPES experiments should be clearly described in the manuscript. In particular, the experimental geometry is a key factor for analyzing spin-resolved ARPES results; meanwhile such information is missed in the manuscript. Lastly, the quality of presented spectra is noticeably lower than that of other recent studies.

We thank the referee for raising these important points. We have substantially expanded the Methods section to clearly describe the ARPES experimental geometry, alignment procedure, and acquisition parameters used in the spin-resolved measurements. Specifically, we now detail the initial normal-emission calibration, the subsequent tilt used to align the K-M-K′ direction with the analyzer slit, and the refinement of the sample orientation via deflector-mode Fermi-surface maps. We also explicitly report the angular and energy scan ranges, step sizes, pass energy, slit settings, and integration times used for both the momentum-distribution (MDC) and energy-distribution (EDC) spin-resolved measurements.

Regarding the data quality, we emphasize that the original ARPES panel in Fig. 2(a) was acquired as a quick orientation scan, since the in-plane band structure of the TaS_2 layer in (SnS)1.15(TaS2) at this photon energy is effectively the same as that of 1H-TaS_2. The purpose of this measurement was therefore solely to identify the relevant momentum cuts for the spin-resolved experiment, rather than to extract detailed spectral information. We note that this is insufficient quality of Nature Communications, and therefore we have replaced this orientation scan with a higher-quality ARPES dataset measured at 56 eV, corresponding to the M point to improve the clarity of our presentation. This updated panel now provides a clearer and more representative reference for the spin-resolved cuts shown in Figs. 2(b–g).

Reviewer #2 (Remarks to the Author):

In this work, the authors study the misfit compound SnS-TaS2, made of quasi-isolated layers of insulator SnS and superconductor TaS2. The authors provide a range of experiments including transport, spin resolved ARPES and STM. The novel contribution is the observation of a two gap structure in STM, which the authors explain in terms of singlet-triplet mixing, which provides different gaps for the spin split pockets at the K point.

I think the approach of using misfits to study 2D SC is very interesting, and the two-gap structure in STM is new for such monolayers (not so much the rest of experiments). I also think it is interesting to perform these measurements to compare with MBE grown monolayers which have shown unexpected behavior. However, I also think the provided theoretical explanation is not the only possible one, and the paper has other drawbacks, so I am not very convinced that the current version belongs to Nat. Comm.

We thank the referee for their positive comments on the experimental aspects of our work and for bringing up other important points based on which we have modified our manuscript as described below.

- First, a main drawback of the current manuscript is that it does not provide a reasonable account of previous research in the misfit compounds. It is not really a novel finding of this manuscript that the spacer layers decouple the H layers and make 2D superconductivity (while the text states "our results… suggest that the SnS layers act as a buffer layer…"). Specifically for SnS-TaS2 misfit, Sankar et al (Chem. Mater. 2018, 30, 4, 1373–1378) reported 2D SC with Tc=3 K. Bai et al (2018 J. Phys.: Condens. Matter 30 355701) reported multiband superconductivity from transport in the SnSe-NbSe2 misfit. A very detailed study on this compound which includes transport, STM and ARPES is only cited in passing as a "high-temperature study" (for STM), but it did observe SC at 3 K too. And finally, a paper with authors in common just published in 2025 (PRB 111, 054509 (2025)) observed many similar features in PbSe-TaS2 except the tunneling experiments. The authors should discuss the previous literature and emphasize the novelty of their results compared to these.

We agree with the referee that the previous version did not sufficiently reflect recent developments in the field of misfit studies.

We have now incorporated additional references, a description of previous work and a description of the new insights provided by our work into the introduction to provide clearer context and better highlight the relevance of the present work.
As the referee notes, the sentence that 'our results… suggest that the SnS layers act as a buffer layer…' is not intended to claim that this is the first study demonstrating the role of monochalcogen layers as buffer layers. Rather, our work provides additional confirmation of this important characteristic of misfit materials. In particular, our local-probe measurements in the superconducting phase offer direct evidence supporting this behavior. We have added this to the paper.

Regarding the specific literature on SnS–TaS_2, we appreciate the referee's observations. The work by Sankar et al. (Chem. Mater. 2018, 30, 1373–1378) focuses primarily on bulk superconducting properties such as the critical field and heat capacity. However, that study does not explore the nature of superconductivity or the characteristics of the superconducting gap.

The paper by Zejun Li et al. (Adv. Mater. 2024, 36, 2312341), as correctly noted by the referee, reports signatures of two-dimensional superconductivity. Their main conclusions rely on transport measurements, including evidence for a BKT transition and critical-field anisotropy, supported by Fermi surface splitting. Their key contribution lies in interpreting the CDW features observed in STM, which did not appear in transport, by combining STM, ARPES, and theoretical calculations. Nevertheless, the superconducting gap structure itself was not investigated in their work.

Our present manuscript directly addresses this missing aspect. Using low-temperature STM, we resolve the superconducting gap structure and reveal clear multicomponent gap behavior in the 1H layer itself, supported by our theoretical modeling. Additionally, our spin-polarized ARPES measurements, performed for the first time on this system demonstrate pronounced spin-split bands along with true 2D electronic nature revealed by our photon energy dependent ARPES data (Supplementary Fig. 2). Our low-temperature STM measurements further reveal superconductivity in the SnS layer, an aspect not previously reported. We also note that our study shows a more complicated structure of the superconducting gap, than simple two s-wave gaps as can be deduced from transport.

Regarding the paper by authors on PbS-TaS2 system (PRB 111, 054509 (2025)), tunneling experiments are critical as a local probe to study the gap structure on each layer, and also to show the existence of a gap in the semiconducting layer. Such experiments provide much more important and interesting information on the gap structure and behavior and not only the number of existence gaps. Note that tunneling was not possible on the previous sample. We tried cleaving and the sample tended to break into pieces. So, in fact the current experiment on the sister sample also shed light on the data on PbS-TaS2.

Thus, our work fills an important gap in the existing literature by providing a comprehensive characterization of the superconducting gap in the misfit SnS-TaS_2 system, and by contextualizing the observed multigap features in relation to those reported earlier in monolayer TaS_2 (Ref. 35). Therefore, we have revised the introduction to have a clearer representation of previous work and address the additional findings done in the current study.

- I appreciate the authors' effort in comparing with the multigap state observed in Ref. 35, which remains a puzzling and important result in the field. However, the authors should state that the coherence peaks of the smaller gap in that work appear around 180 ueV (by eye, the authors may have the data) which is taken at 25 mK. That paper also states the smaller gap disappears at 400 mK, even though Tc is 3K. Since the STM data is taken at 300 mK, why is the gap in STM (300 ueV) larger at larger temperature? If STM is done at say 500 mK is the gap still detectable? I am not sure the gap in Ref. 35 is the same feature as the one observed here.

We thank the referee for this important point.

To start with, we have reanalyzed our data and fitted the spectra using the gap structure suggested by our s+f wave model (see Supplementary Data for further details and Fig. 4 for the fits), which yields a small f-wave gap magnitude of 140 μeV. We note that we have seen gaps of

similar magnitudes at our effective temperature of ~400mK in many other systems so it is not surprising that we observe this gap in our STM data.

Let us now address the correspondence between what we see and what was measured in ref. 35.

Shown in the figure on the right is a simulation of what the spectrum seen in Ref. 35 should look like at 500mK. While thermal broadening clearly suppresses the coherence peaks, clear kinks (shown by arrows) are visible at the energy of the second gap. Note that thermal broadening also increases the size of the measured gap energy scale which always happens for superconducting gaps measured at non-zero temperatures. Moreover, the smaller energy scale in our spectra, given by the fit results (s-f = 250 μeV), is similar to the in-gape coherence peaks energies (230 μeV) in ref. 35, according to the published data.

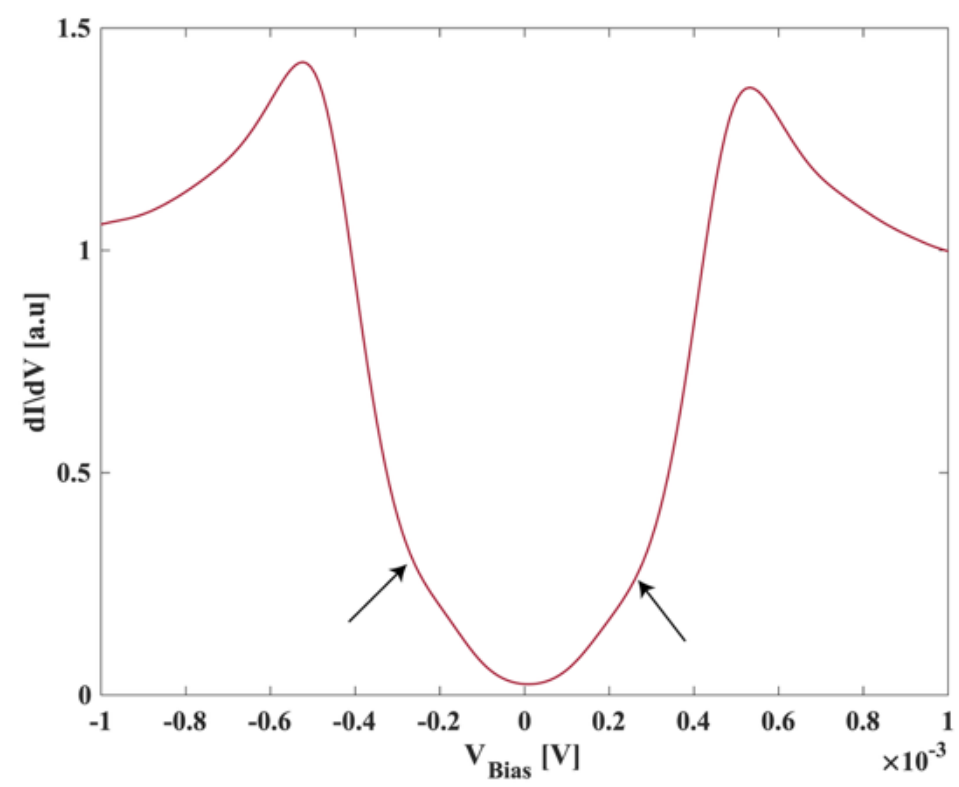

The disappearance of the smaller gap at 400mK in ref. 35 is therefore not intrinsic to the system but might be result of noise.

Thus, the analogy between the two studies is reasonable. The small difference in gap sizes between the two studies could be a result of small differences between the environment of the 1H layer in the two studies.

A main difference between these two experiments is that planar junctions enhance the visibility of small momentum states compared to point junctions (see Berthod, PRB 84, 155414 (2011)). Since monolayer TaS2 has a d-derived Gamma pocket in addition to those at K/K', it is possible that the planar junction is observing the SC gap of this band.

We thank the referee for this point, and we want to note that although momentum is not preserved in the STM tunneling experiment, the loss of density of states in the superconducting phase transition will result in its visible effect in the spectra.

We also wish to emphasize that the Γ-pocket in 1H-TaS2 is formed by an extension of the same band that surrounds the k points, therefore the probability to tunnel into states with momentum around the edge of the Brillouin zone should be similar to the probability to tunnel into states which emerge from the same band and occupy momenta closer to the Γ point.

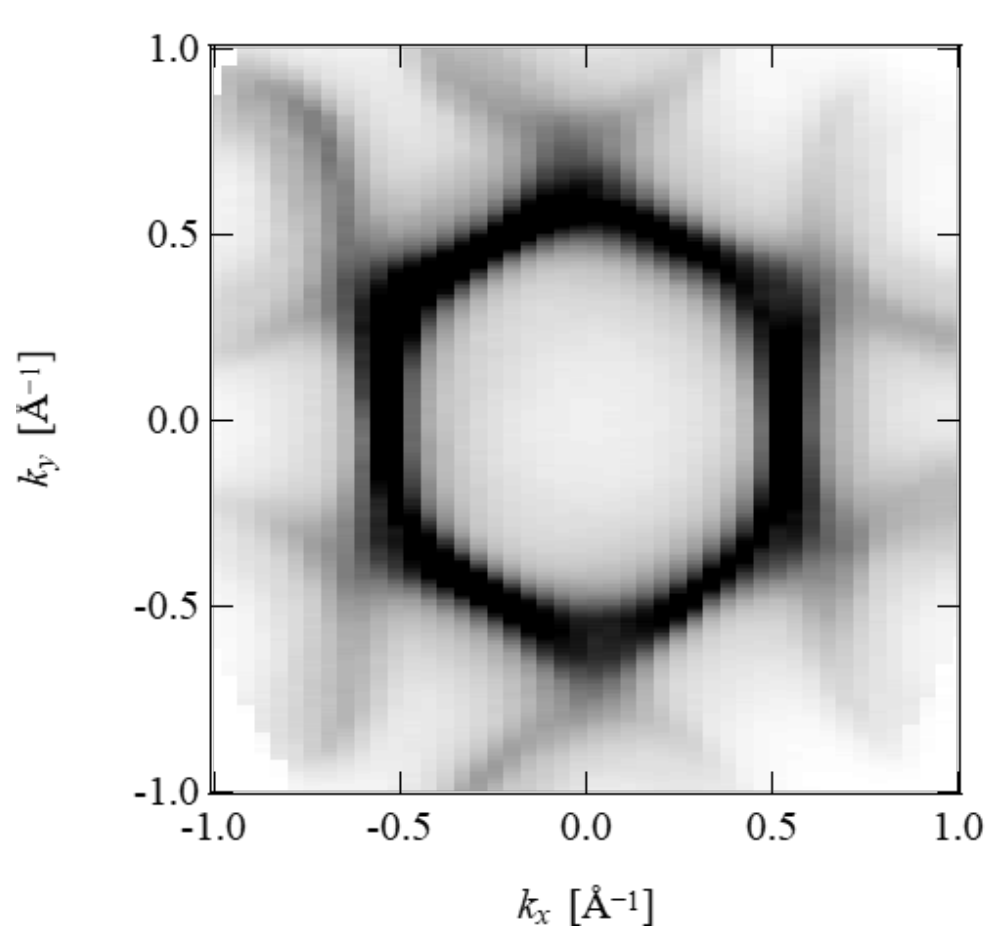

Furthermore, tunneling into states around the Γ point is seen in various superconducting measurements such as in $FeSe_{0.5}Te_{0.5}$ [Chen, G. *et al.* "*Evidence for a robust sign-changing s-wave order parameter in monolayer films of superconducting Fe (Se, Te)/Bi2Te3*". *npj Quantum Materials 7*, 1 (2022)] and $NbSe_2$ [Noat, Y. *et al.* "*Quasiparticle spectra of 2 H-*

NbSe 2: Two-band superconductivity and the role of tunneling selectivity." Phys. Rev. B 92, (13)]. In both cases the superconducting gap which resides on the Fermi pocket around the Γ point is visible in STM experiments, even though the Fermi pocket around the Γ point is smaller compared to that in 1H-TaS2, seen in the attached Fermi surface spectra acquired in our ARPES experiment.

Moreover, as pointed out and emphasized in the revised paper, we believe the origin for both of those effects, in our study and in ref. 35, is due to two component order-parameter on the same band. This conclusion is based upon the known gap structure of bulk 2H-TaS2 where a single s-wave gap exists, as was measured in ref. 35 and ref 82. When the monolayer limit is reached, the band structure remains the same up to the inter-layer coupling effects as seen by our ARPES data, yet a substantial change in the gap structure is seen.

- Regarding the STM double gap, it is also known in these compounds that due to the momentum dependence of electron-phonon coupling, the gap at K is strongly anisotropic (see for example ARPES in PRB 85, 224532 (2012) and PRL 119, 087004 (2017) for a DFT calculation). This could be a natural explanation for the apparent two-gap structure. Can the authors provide evidence on the contrary?

We thank the referee for drawing our attention to this point.

First, we note that the anisotropic electron–phonon coupling exists in bulk 2H-TaS2 [Wijayaratne et al., Journal of Materials Chemistry C 5, 43 (2017)], but the gap is isotropic s-wave. So anisotropic electron–phonon coupling alone cannot explain our data.

The referee is correct in pointing out that the APRES study in Phys. Rev. B 85, 224532 (2012) suggests that the anisotropic e-ph coupling may be connected to the anisotropy of the superconducting gap. But even they don't claim a causal relationship. For example, in their conclusions they say: ` However, while the anisotropy of the SC gap is partly reflected in the measured **k** dependence of the electron-phonon coupling parameter, there is no clear connection between the CDW gap and the nesting properties of the Fermi surface.', implying that there is at best a small correlation between the existence of the two.

Moreover, we would like to draw attention to some aspects the ARPES data. First, the authors show an energy resolution of 4 meV at best, while trying to measure a gap of 1.5 $\pm$0.5 meV. Second, according to their results the superconducting gap maximum is given as a fit result of 2.5 meV, which is about double the size of a superconducting gap measured by tunneling experiments (taken by the coherence peaks locations). In their experiment the CDW and SC gaps are indistinguishable and are seen constructively as a reduction in spectral weight. This can cause an anisotropic CDW gap to influence the signature of the SC gap. Such drawbacks in ARPES studies, even in higher Tc superconductors, show the need for high resolution and direct measurements of the superconducting gap and its structure on TMDC's as in STM.

Overlooking these issues, a robust two-gap structure has been firmly established [Noat et al., Phys. Rev. B 92, 134510 (2015)] in NbSe2 but the multigap spectrum in NbSe2 is understood to originate from band-selective superconductivity associated with the Se-derived band centered at the Gamma point, rather than from gap anisotropy at the K point [Nature Communications, volume 9, Article number 598 (2018)].

This qualitative difference between NbSe2 and TaS2, highlights the distinct role played by dimensionality and inter-layer coupling in these two materials. The anisotropic electron-phonon coupling cannot be responsible for creating the multi gap feature in the spectroscopic data presented in our study. For $NbSe_2$ the proposed mechanism of anisotropic e-p coupling or two bands at the Fermi level, both of which persist down to the monolayer limit, also show such spectra in bulk form. By contrast, in the case of 1H TaS2, the unique spectroscopic signature emerges only in the monolayer limit, despite the presence of anisotropic electron–phonon coupling already in bulk 2H TaS2 [Wijayaratne et al., Journal of Materials Chemistry C 5, 43 (2017)]. Nevertheless, we add this to our discussion for further elaboration.

- I am also very confused by the theoretical model. Fig 1b and the text that describes the structure ("here the 1H layers maintain the same orientation, making the crystal lattice lack overall inversion symmetry") clearly show that the stacking of the H layers breaks inversion (unlike the 2H structure or the H layers in the 4Hb structure, which alternate in orientation and have an inversion center between the layers). But the theory is built for a globally symmetric system, so it applies to 4Hb and 2H, but not to the misfit. The labeling of irreps is also confusing. The authors state that the point group is D3, which is not correct. The mentioned irreps are either those of D6h (like in 2H or 4Hb) or D3d. But the group of a monolayer is D3h, again with different irreps.

We thank the referee for raising this important point and agree that the theoretical framework was presented in a confusing manner in the original manuscript.

Regarding the point-group symmetry, the referee is correct: the appropriate point group for the centrosymmetric reference structures is D6h rather than D3. In our analysis, we restrict ourselves to the one-dimensional irreducible representations A1g and A2u of D6h, which serve as convenient labels in the centrosymmetric limit. The corresponding monolayer symmetry is D3h, which differs from D6h by both the absence of inversion symmetry and a reduced rotational symmetry. In this noncentrosymmetric setting, pairing channels that originate from opposite-parity representations in the centrosymmetric limit are no longer symmetry-protected and can therefore mix.

We also acknowledge the referee's correct observation that the experimental misfit compound lacks a global inversion center, whereas the theoretical model we employ is formulated for a centrosymmetric reference system, such as 2H–TaS2 or 4Hb–TaS2. Our model is not intended as a microscopic description of the misfit structure itself. Rather, it is a minimal framework designed to isolate the role of interlayer coupling and inversion symmetry in shaping the superconducting density of states.

The central puzzle motivating this approach is the following: why is a pronounced multigap feature observed in monolayer 1H–TaS2 and in the misfit compound, but absent in bulk 2H–TaS2 and 4Hb–TaS2? Within our model, this behavior is traced to the effect of interlayer coupling on pairing states that arise in an inversion-broken monolayer. When two layers with opposite orientation (H and H′) are coupled, inversion symmetry is effectively restored and the local symmetry breaking on each layer becomes less prominent as the coupling increases, leading to the suppression of the multigap feature. By contrast, when layers of the same orientation (H–H) are coupled, inversion symmetry remains broken and the corresponding mixing effects persist. This distinction captures, at a qualitative level, the experimentally observed difference between centrosymmetric and noncentrosymmetric structures over a range of material realizations.

- Assuming that the right model is the one for a monolayer, there are DFT calculations of the splitting of the gap at K/K' (see npj Comp. Mat. 9, 66 (2023)) due to Ising SC. The inner/outer gap difference is small both with phonon and spin fluctuation pairing (which might be loosely considered as originating from Coulomb interaction). The splitting obtained with the model in this work is quite large compared to the DFT calculations (which are of course not really comparable), but this raises the question of whether the model is really reasonable. Again, rather than singlet-triplet mixing, I think gap anisotropy might be a more likely explanation.

We thank the referee for this informative comment and for pointing out the recent DFT results on Ising superconductivity at the K and K′ points.

We agree that both scenarios, an anisotropic gap and the singlet-triplet mixing, lead to similar spectroscopic signatures in tunneling experiments and therefore cannot be distinguished on the basis of the density of states alone. The motivation for considering singlet–triplet mixing in our case is not based solely on experimental tunneling spectra, but rather their systematic dependence on crystal symmetry. In particular, the disappearance of the multigap feature in inversion-symmetric structures, and its persistence in noncentrosymmetric ones, is naturally captured by our model. We do not claim quantitative agreement with existing DFT calculations; instead, our goal is to provide a qualitative mechanism that links the observed phenomenology to the presence or absence of inversion symmetry and to the strength of interlayer coupling.

We also want to point out that even in the cited paper (which again only address NbSe2 where such anisotropy is also suggested to appear in the bulk) the calculations agree that "... is that unmitigated EPC is way too strong to be consistent with the experiment…" and that "...This essentially excludes the possibility of a predominantly triplet state, albeit generates a small (≈ 10%) triplet admixture." thus suggesting that the calculated EPC might be stronger than realistic values and that the actual triplet admixture can be higher. Furthermore, a cited paper in that work [Wan, W. et al. "Observation of Superconducting Collective Modes from Competing Pairing Instabilities in Single-Layer NbSe2." *Advanced Materials* 34, 41 (2022)] (ref. 89 in our revised manuscript) suggests the existence of such admixture in NbSe2 monolayer, thus reinforcing our picture of additional pairing mechanisms in the monolayer regime, where interlayer coupling vanishes.
As we noted in the previous comment, indeed the anisotropic electron-phonon coupling may influence the spectroscopic data, by creating an anisotropic gap, but in that case, we would expect such an effect to also occur in bulk 2H-TaS2.

Other minor comments:

- The authors state in the introduction that SnS decouples the 1H layers and that this conclusion is evident from the sample Tc 3.1 K. However, at this point they have not mentioned the fact that Tc goes from 3 K in the monolayer to 0.7 K in bulk, so why is this "evident" it is unclear for a non-expert reader. Since the authors then say this explicitly further down in the text any way, I suggest moving that sentence upwards so that the evidence is easily understood (ideally when they cite all previous works that have already seen this).

We thank the referee for this comment. We fixed the relevant sentence in the introduction.

- I am surprised by Refs 36 (about moire TMDs) and 37 which is about insulating WSe2. The sentence is about phonon softening in metallic 2H TMDs which have a CDW transition (in the bulk, all do except NbS2). The authors should provide supporting references.

We thank the referee for pointing this out. We have changed the reference to more relevant citations. We add the next citations [Lin, D. *et al. "Patterns and driving forces of dimensionality-dependent charge density waves in 2H-type transition metal dichalcogenides." Nat. Commun.* 11, 2406 (2020); Ugeda, M. *et al.* "*Characterization of collective ground states in single-layer NbSe2"*. *Nat. Phys.* 12, 92–97 (2016); Yang, Y. *et al* "*Enhanced superconductivity upon weakening of charge density wave transport in 2H-TaS2 in the two-dimensional limit." Phys. Rev. B*, *98*, 3 (2018).]

- Regarding unconventional pairing in NbSe2 I believe Cho et al, PRL 129, 087002 (2022) is as relevant as Ref 31, the authors may consider citing it.

We thank the referee for the suggestion and added PRL 129, 087002 (2022) to our reference list (ref 88 in the revised manuscript).

- The title of Ref. 53 made the claim of an f-wave OP, but I encourage the authors to critically evaluate if the reported evidence poses any constraints on the symmetry of the order parameter. I think it is best to refer to this as a V shape gap.

We thank the referee for this comment. Indeed, the study we cited (ref 54 in the revised manuscript) proposes that the V shapes spectra originate from an f-wave order parameter. It is known that a V shaped spectra can only be acquired on materials with nodal gaps, given very low effective temperature. Considering the 6-fold symmetry of the 2H structure of the 1H layer, it is natural to assume that the f-wave gap is the most plausible scenario. Yet, other order parameters such a nematic two component order parameters can also result in similar V-shaped spectra. We therefore make use of the referee's suggestion and refer to it as a V-shaped nodal gap in our paper.

Reviewer #3 (Remarks to the Author):

The manuscript investigates unconventional superconductivity in the misfit compound (SnS)1.15(TaS2), composed of alternating SnS and 1H-TaS2 layers. By combining transport, ARPES, and STM spectroscopy, the authors suggest that the SnS layers electronically decouple the TaS2 sheets, leading to quasi-two-dimensional superconductivity with a clear two-gap feature. A theoretical model incorporating broken inversion symmetry and finite-range pairing interactions reproduces the observed mixed singlet-triplet character. Overall, the work provides new insights into how unconventional pairing emerges in isolated 1H-TaS2 layers and highlights misfit compounds as a promising platform for exploring multi-gap and parity-mixed superconductivity. However, several aspects of the study remain insufficiently addressed, which currently limits the clarity and rigor of the manuscript. I am unable to recommend the manuscript for publication in Nature Communications at this stage. However, it may become suitable once the authors have carefully addressed the points detailed below.

We thank the referee for their positive assessment of both the theoretical framework and the experimental results presented in our work. We also appreciate the additional insightful comments, which have helped us further improve the clarity and rigor of the manuscript. In response, we have revised the paper accordingly, as detailed below.

(1) Figures 2 and 3 present the transport and ARPES measurements. However, since these are non-local techniques and the SnS layer is also reported to be superconducting, it is crucial to clarify the role of the SnS layer. The discussion of this aspect is currently insufficient and somewhat unclear. For instance, the authors state in the main text (lines 159-163) that the coherence length extracted from the vortex agrees with that estimated from the upper critical field. Yet, in Supplementary Material V, the vortex is shown in the SnS layer, and no data is provided for the vortex in the 1H-TaS2 layer. This inconsistency is confusing and undermines the logical coherence of the related discussion. Considering that a two-gap model was used in Fig. 2b to fit the evolution of the upper critical field, it appears that the authors attribute the transport and ARPES results solely to the TaS2 layer, while the SnS layer is only included later in the self-consistent analysis (e.g., the discussion of the coherence length). I believe the authors need to provide clarification on these issues.

We thank the referee for raising this important point, which provided us with a valuable opportunity to further clarify the manuscript.

We would like to emphasize that bulk transport measurements, as correctly noted by the referee, probe the response averaged over the entire sample. Consequently, such measurements do not allow us to disentangle the individual contributions of the two layers or to determine which gap is more pronounced.

However, the pronounced upward curvature of $H_{c2}(T)$ provides strong evidence that both the SnS and the 1H-layer gaps contribute to the transport response. Such behavior is characteristic of multi-gap superconductivity, indicating that the superconducting properties of both layers influence the measured critical field.

Given this unexpected result, we employed vortex imaging in the SnS layer to directly demonstrate the presence of a superconducting gap in that layer. In clear contrast, in 4Hb-TaS2 no vortices were observed in the 1T layer, underscoring the distinct role of the SnS layer in the present system.

Moreover, the vortex imaging measurements and the coherence length extracted from them are quantitatively consistent with the values inferred from transport data. This agreement provides independent validation that the SnS layer is intrinsically superconducting and actively contributes to the bulk response.

Taken together, these findings highlight the crucial role of the SnS layer in interpreting transport and bulk-averaged measurements in misfit compounds, as discussed in detail in the Results section.

Furthermore, the CDW order observed in the STM topography of the 1H layer is clearly absent in the transport data. This contrasts with clean TaS2 samples, where the CDW transition is readily detected in bulk transport measurements. The absence of a discernible CDW signature in transport in the present system further indicates that the SnS layer significantly influences sample-averaged measurements and may obscure the CDW contribution of the 1H layer.

Taken together, these independent observations consistently point to the important role of the SnS layer. We now emphasize this more clearly both the Results and Discussion sections regarding transport and STM results of CDW and SC states. We explicitly state that the superconducting gap in the SnS layer is intrinsic and independent, while also clarifying its apparent impact on bulk-sensitive measurements such as transport.

(2) The main text (lines 120-121) refers to Fig. 2c, but such a panel is not included in the currently presented Figure 2.

We thank the referee for noticing it and raising this point. We fix this typo.

(3) In a previous work (Adv. Mater. 2023, 35, 2305409), nodal superconductivity has been reported in the pristine monolayer 1H-TaS2. I believe the authors should discuss the origin of this discrepancy about the gap features in the manuscript.

We thank the referee for this insightful comment. In response, we have substantially elaborated on this point and expanded the Discussion section considering our findings.

The revised text now provides a detailed comparison between our experimental results, such as the measured T_c in both samples. Furthermore, we address the possible role of disorder, as suggested by the authors of that study. We also discuss the potential influence of strain effects induced by the HOPG substrate, which may modify the superconducting properties and contribute to the observed behavior.

This expanded discussion clarifies the distinctions between the two systems and places our results in a broader physical context.

(4) As suggested by the theoretical model presented in the manuscript, the superconducting state without interlayer coupling appears weaker than that with interlayer coupling, which contrasts with the experimental findings. It would be helpful if the authors could further discuss this discrepancy.

We thank the referee for raising this important question. The enhancement of the superconducting transition temperature upon reducing the thickness in 2H–TaS2 is anomalous among transition-metal dichalcogenides, and its microscopic origin remains an open problem. In particular, this behavior is not captured by simple weak-coupling BCS-type models and is likely influenced by additional effects beyond the scope of our theory, such as the competition between superconductivity and charge-density-wave order, whose strength is known to depend on dimensionality.

Our theoretical model is therefore not intended to reproduce the thickness dependence of Tc observed experimentally. Instead, it is designed to address the evolution of the superconducting gap structure as a function of interlayer coupling, assuming fixed pairing interactions. Within this framework, the enhancement of Tc with increasing interlayer coupling arises trivially from the increase in the electronic density of states at the Fermi level and does not reflect the full microscopic physics governing Tc in the real material. We emphasize that this limitation does not affect the main conclusions of our work, which concern the presence or absence of multigap features and their relation to inversion symmetry and interlayer coupling

(5) Given that multiple mechanisms can give rise to two-gap superconductivity, the authors have not provided sufficient justification to exclude alternatives other than the mixed singlet-triplet pairing scenario. In Supplementary Material VI, ARPES data are presented to demonstrate a vanishing interlayer coupling between the 1H-TaS2 layers; however, this evidence appears insufficient. Although the manuscript states that the superconductivity in SnS does not originate from the proximity effect of TaS2, it remains important to further clarify whether any coupling exists between the superconducting states of the two layers, and how such coupling, if present, might influence the main conclusions.

We thank the referee for this important comment.

To begin with, we note that the revised manuscript now contains a substantially expanded Discussion section, in which we examine additional possible mechanisms in greater detail.

Specifically, we consider whether our results could be explained within the McMillan framework for tunneling into a two-band superconductor. To address this possibility qualitatively, we have added Supplementary Fig. 13, which demonstrates that such a scenario cannot reproduce the key features of our data and is therefore unlikely to account for the observed two-gap structure.

We also discuss the potential role of anisotropic electron–phonon coupling. In this context, we compare our findings with bulk 2H-TaS2, where a similar anisotropic electron–phonon interaction is known to exist, yet no two-gap structure is observed. This comparison further supports our conclusion that anisotropic electron–phonon coupling alone cannot explain the emergence of the two-gap behavior in our system.

Regarding the possibility that the superconducting gap observed in the SnS layer originates from proximity coupling, we present the magnetic-field dependence of the gaps in both layers (Supplementary Fig. 6). We find that the field evolution differs markedly: at $B = 0.1$ T, the gap in the 1H layer exhibits substantially stronger spectral filling than the gap in the SnS layer. Such asymmetric behavior is not expected if the SnS gap were merely proximity-induced from the 1H layer.

In addition, we performed a more realistic fit within our theoretical framework. A model incorporating an admixture of s-wave and f-wave components (see Supplementary Data) yields a smaller s-wave gap in the 1H layer than the gap extracted for the SnS layer. This hierarchy of gap magnitudes is incompatible with a simple proximity scenario and further supports the conclusion that the SnS gap is intrinsic rather than proximity-induced. The new fit and a clearer discussion of the magnetic field dependence is now added to the revised manuscript.

To further substantiate the claim of decoupled layers, we reanalyzed our ARPES data and expanded the Supplementary Material (including the revised Supplementary Fig. 2) to present a quantitative tight-binding calculation of the expected k_z-dependent dispersion for both finite and vanishing interlayer coupling between the two 1H layers of TaS2. We now explicitly describe the model, show the calculated dispersions, and directly compare them with the photon-energy-dependent ARPES measurements. The analysis demonstrates that only the $t = 0$case reproduces the experimentally observed absence of k_z-dependent band splitting, thereby providing strong evidence for negligible interlayer coupling.

Taken together, our layer-resolved gap spectroscopy, the magnetic-field evolution, the refined theoretical modeling, and the ARPES analysis consistently indicate that the SnS and TaS_2 layers are electronically decoupled. This decoupling provides a solid foundation for our theoretical framework, in which the two-component gap structure emerges intrinsically within monolayer 1H-TaS_2, rather than as a consequence of interlayer proximity effects.

REVIEWER COMMENTS

We thank the referees for finding the revised manuscript clearer and more insightful. We thank referee #2 for recommending our manuscript for publication. Referees #1 and #3 have raised additional questions before recommending publication in *Nature Communications*. In response, we have revised the appropriate parts and answered the concerns in a point-by-point manner.

We mark changes in the manuscript and supplementary data in red color.

Reviewer #1 (Remarks to the Author):

I appreciate the authors' effort in revising the manuscript, supplementary material, and discussions therein. Now the manuscript is improved, especially in the ARPES method and in the broader literature discussion. Following the authors' argument, the key finding of this work is the mixed order parameter due to the absence of inter-layer coupling. They also argued that the proximity effect from SnS layers must be irrelevant to their observation of two-gap superconductivity. However, I still cannot recommend the work for publication in Nature Communications since my main concerns raised in the previous review—that the key findings seem incremental, and the support is insufficient—have not been resolved.

We thank the referee for finding the revised manuscript improved in terms of Data and theoretical model presentation and the discussion level. We want to emphasize that our data is **new** in terms of **tunneling spectroscopy of the superconducting state** and the use of **inherent broken inversion symmetric** material. As for the concern of incremental findings, as we describe below, our paper presents new data in the field of TMDC's and misfit layers compounds.

(i) 4Hb-TaS_2 is a material with an overall inversion symmetric unit cell, while in our system the whole unit cell, as well as the individual constituent layers, ***lack inversion symmetry.***

(ii) From the Fermi surface point of view, the band structure of the material is different. In 4Hb-TaS_2 additional Fermi pockets arise from the spacer 1T layer, while our sample, as shown in the previous rebuttal letter, ***lacks the additional Fermi pockets thus giving an example of a clean 1H-TaS_2 layer embedded in the single crystal.***

(iii) While previous transport studies on related misfit materials have suggested the presence of two-gap superconductivity, such evidence remains indirect. In the present

work, we systematically investigate the superconducting gap structure in a layer-resolved manner and complement it with a generalized theoretical framework that accounts for the observed behavior in the limit of vanishing interlayer coupling.

First, this work is not the first one reporting the vanishing of interlayer interaction in a misfit compound. Some of the authors published a paper on evidence of vanishing of interlayer interaction in (PbS)1.13(TaS2) [Ref. 60 in the main text] and suggested potential two-gap superconductivity from critical field measurements.

We thank the referee for raising this point, giving us the opportunity to emphasize this distinction. In this work we obtain **local tunneling spectra** which is the **most direct way of probing the superconducting gap in a layer dependent way. We do not claim to be first to show vanishing inter-layer coupling, rather its implications on the superconducting phase.**

While the cited work does show the signatures of a two-gap superconductor, in this study we explicitly show important **layer-resolved data of the superconducting gap**. We show the structure of the gap in each layer ***(most notable in the SnS layer which was assumed semiconducting until now)*** **and the importance of both gaps in the combined behavior of the material in transport data.** This represents a qualitatively stronger and independent verification of multigap superconductivity in this class of misfit materials.

Most importantly, the previous study does not show the existence of a multi-component gap structure in the 1H layer, rather a two-gap behavior in the sample as a whole. In our case we show that the misfit layer compounds give the ability to study unconventional superconducting-state arising from the combination of broken inversion symmetry and vanishing inter-layer coupling. It is important to **distinguish between the two different superconducting layers, the SnS and the 1H,** as we present here, and the **two-component order parameter measured in the isolated 1H-layer** by itself which is **a completely new result.**

In addition, there are also concerns on the claim itself. In the Supplementary Material, the authors put together additional ARPES data on examination of kz-dispersion along the M-L high symmetry points. Though the energy feature at -0.325 eV (the description in line 167 saying -0.325 meV seems like a typo) is dispersionless along the kz axis at zone boundary, it does not explicitly confirm that only the charge transfer between the SnS and TaS2 layers is relevant near EF. Such a characteristic would require further verification from termination dependence of ARPES spectra as well as core level examinations.

We thank the referee for noticing the typo, which is now fixed. Regarding the claim of the band structure not affected by the spacer layer, we want to clarify that our ARPES data show both a **dispersionless band along k_z and a charge transfer mechanism**. The former observation is due to having the inter-layer coupling smaller than our ARPES resolution. We start by noting that we revised Supplementary Fig. S4 and added a new Supplementary Fig. S5 of a simulation of the expected energy distribution curves at the M and L point of the Brillouin zone in the case of an inter layer coupling of t=15 meV. The simulation shows a clear difference between the EDCs, thus reinforcing our notion that the inter-layer coupling is negligibly small and constrained to be below 10 meV.

Here we present the revised Supplementary Fig. S4 (Fig. 1 of this rebuttal letter) where the direct comparison shows the difference between a band structure with dispersion along Kz and the lack of it, which justify the notion of vanishing inter-layer coupling. By varying the photon energy and comparing the results with 2H-TaS_2 data (ref. 42 and the revised Supplementary Fig. S4) and band-structure calculations, we verify the inter-layer coupling between the layers is smaller than 10meV.

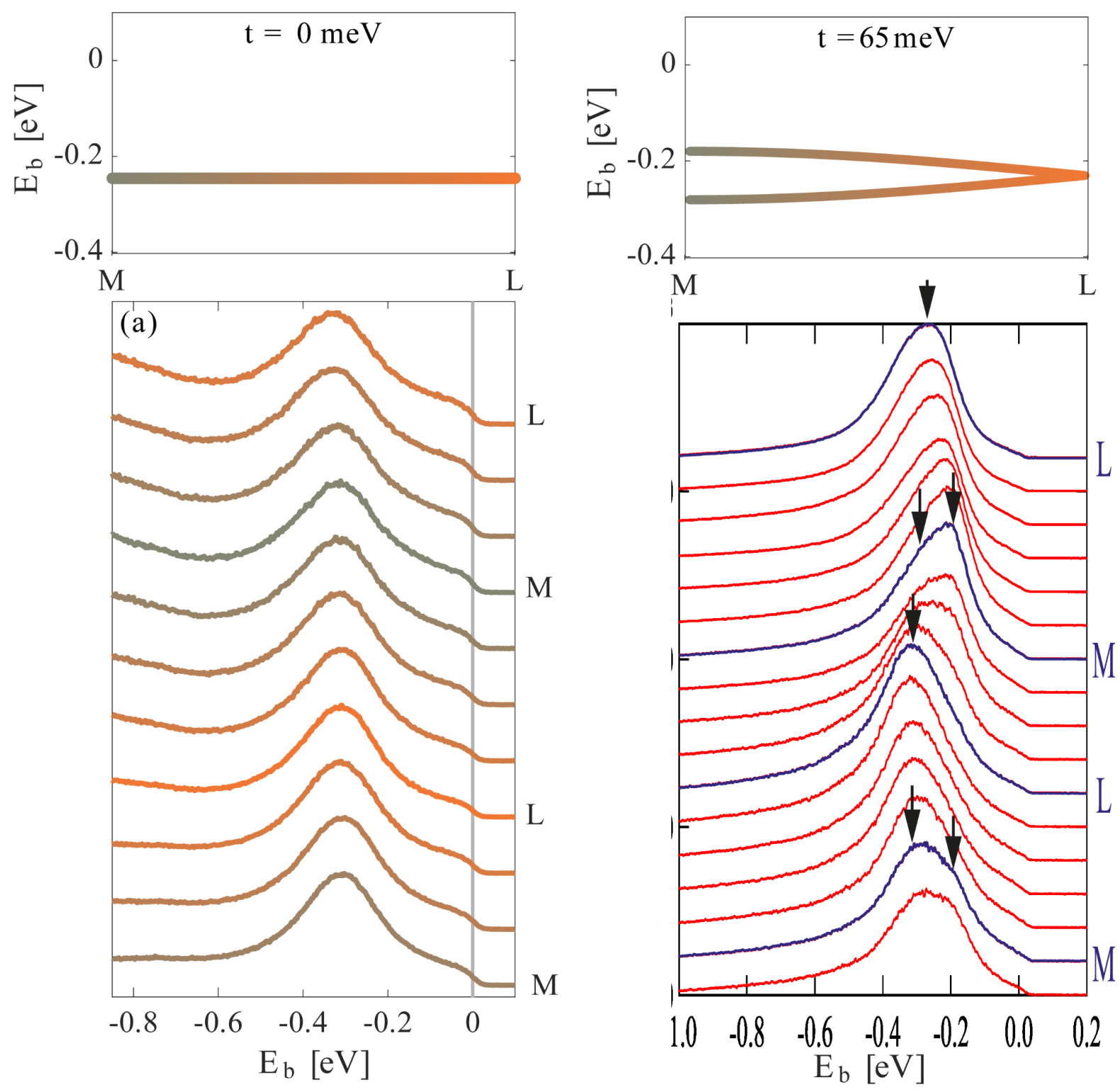

Figure 1 – **Top left**: Calculated band dispersion along the M-L line for an inter-layer hopping of t=0~meV, showing the absence of any kz dependent gap. **Bottom left**: Energy distribution curves (EDCs) taken along the M-L direction of the 1H-TaS2 Brillouin zone. The different kz values are accessed by varying the photon energy from 49 to 59eV, covering approximately one and a half Brillouin zones along kz. The gray line marks the Fermi level. **Top right:** Same calculation as in a for t=65mev, showing a kz dependent bonding antibonding splitting. The comparison demonstrates that the experimental data are consistent with a vanishing effective inter layer coupling between two consecutive 1H layers in the misfit compound. **Bottom right:** EDCs taken along the M-L direction of the 2H-TaS2 Brillouin zone. Adopted from ref 42.

We show in Supplementary Fig. S4 and S5 that for finite interlayer coupling the M point and the L point spectra are substantially different, as the L point lacks a gap from symmetry considerations. In the M point the splitting of the peak at -0.325 eV is the largest and a dispersion will exist as was measured before in ref 42, for bulk 2H-TaS_2.

The vanishing inter-layer coupling and the latter statement regarding the charge transfer are both visible also by directly comparing the ARPES data along the K-M-K line taken on the pristine polymorph 2H-TaS_2, the heterostructure 4Hb-TaS_2 and the misfit compound SnS-TaS_2, Fig. 2 of this rebuttal letter. Published data adopted from ref. 42 is used to directly show the vanishing inter-layer coupling similar to the 4Hb case and opposite to the 2H case, and equally important is the larger bandwidth which points to a significant charge transfer mechanism, Fig. 2 of this rebuttal letter. Moreover, **the sharpness of the bands in ARPES data is another indication of the k_z dispersionless bands** [Almoalem et al. "Charge transfer and spin-valley locking in 4Hb-TaS2." *npj Quantum Materials* 9.1 (2024)] *npj Quantum Materials* 9.1 (2024)]. The lack of different peaks in a single spectrum, the dispersionless EDC's, the spin polarized bands (Figure 2) and the sharpness of the bands all point towards a vanishing inter-layer coupling.

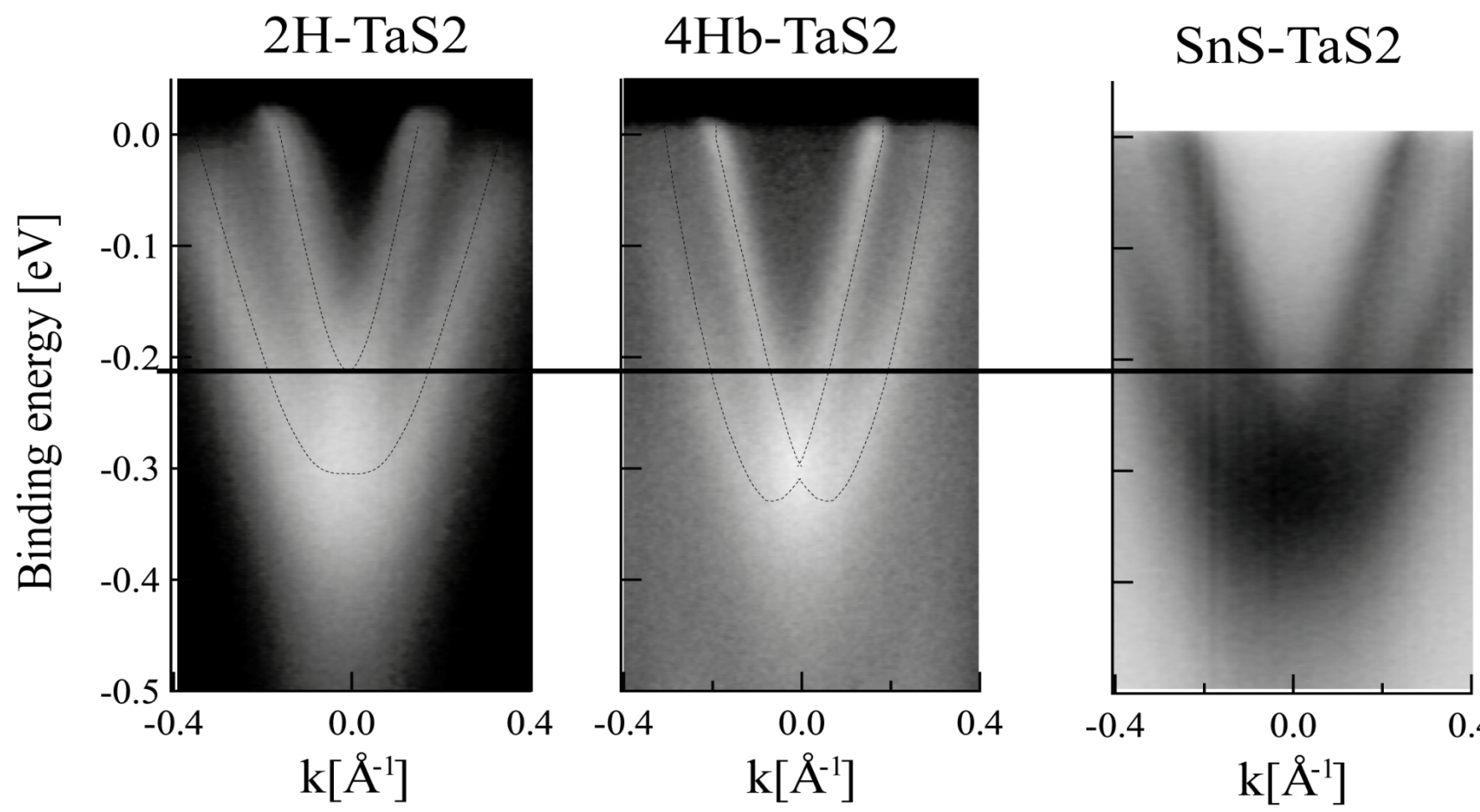

Figure 2 – **Left to right**: ARPES spectra taken along the K-M-K' line in 2H-TaS2, 4Hb-TaS2 and SnS-TaS2 misfit compounds respectively. Horizontal line marks the bottom of the upper band in 2H-TaS2. The bottoms of the band in 4Hb-TaS2 and SnS-TaS2 is vividly seen to be in much deeper than in 2H-TaS2, and the spectra in 4Hb-TaS2 and SnS-TaS2 are similar pointing on similar band structure in both polymorphs.

Furthermore, the Fermi surface contour, as was shown in the previous rebuttal letter, shows that no additional pockets, or renormalization of the bands occur at the Fermi level, compared to pristine 2H-TaS_2. Hence, it is fair to assume charge transfer as the only relevant phenomenon within the ARPES resolution.

Termination-resolved ARPES could indeed provide additional insight; however, it lies beyond the scope of the present work. Importantly, such measurements would not alter the main conclusions drawn from our ARPES data: namely, the negligible interlayer coupling and the absence of any additional Fermi pockets.

Nevertheless, we also add the next comment to the results section to further clarify the vanishing of the inter-layer coupling:

"The vanishing inter-layer coupling is further shown by comparing the EDC at the L point, where the gap is closed from symmetry considerations [41,42], and the M point where the gap is the largest for a given coupling. The two EDC are identical, as opposed to pristine 2H-TaS2, thus reinforcing the conclusion that the inter-layer coupling is smaller than our ARPES resolution of 10 meV, Supplementary Fig. S4 and S5."

Secondly, 4Hb-TaS2 presents a benchmark for the authors' theoretical framework, meanwhile they make contradictory statements regarding the two-gap behavior in it. According to the authors' response to Reviewer #2, 4Hb-TaS2 must match well with the theoretical model in the main text as it is inversion-symmetric with suppressed interlayer interaction. If interlayer decoupling alone without inversion symmetry breaking were sufficient to produce two-gap features, one would expect 4Hb-TaS_2 to exhibit similar spectroscopic signatures to (SnS)1.15TaS2. yet no STM study on 4Hb-TaS_2 has reported two distinct coherence peak energies as claimed in the present work. In addition, authors' explanation with impurities remains speculative.

We thank the referee for raising this important point and for prompting us to clarify the scope and limitations of our comparison.

We would like to emphasize that, within our theoretical framework, the most direct and physically meaningful comparison is between 1H and 2H structures. These two systems differ simultaneously in inversion symmetry and in the strength of interlayer coupling and therefore represent the two limiting cases of the model presented in the manuscript. The evolution between these limits is precisely what our framework is designed to capture.

In this context, 4Hb-TaS_2 is a significantly more complex system, and the nature of its superconducting state remains under active debate. Therefore, a direct comparison within the simplified framework of our model is not expected.

Importantly, multiple experiments point toward nematic and chiral superconductivity in 4Hb-TaS_2, consistent with a two-component order parameter belonging to a two-dimensional irreducible representation. This is qualitatively distinct from the situation considered here, where the two gaps arise from order parameters belonging to one-dimensional irreducible representations that can mix due to symmetry breaking.

At the same time, the weak interlayer coupling in 4Hb-TaS_2 suggests that singlet–triplet mixing may occur, similar to the mechanism we propose for $(SnS)_{1.15}TaS_2$. While STM studies on 4Hb-TaS_2 have not reported clearly resolved double coherence peaks, they do reveal a finite residual density of states in the superconducting phase, which has been

interpreted as evidence for an additional superconducting component that becomes effectively gapless due to disorder. Furthermore, recent thermal transport measurements are consistent with a multi-gap scenario.

Taken together, these considerations indicate that the absence of clearly resolved two-gap features in STM does not constitute a contradiction to our framework but rather reflects the greater complexity of 4Hb-TaS_2 and the distinct nature of its superconducting state.

Lastly (and on minor notes), the manuscript still needs improvement in writing as it still has technical errors in several places.

In line 61, “mono-chalgoen” must be “mono-chalcogen”.

In line 148, Fig. 2c does not exist while the sentence refers to it. (Reviewer #3 already pointed this out in their previous comment.)

In line 191 and 192, “wether” must be “whether”.

In line 301, “the where inversion” may be “where the inversion”.

In line 360, “1d” may be “as shown in Fig. 1d”.

We thank the referee for these important corrections. Indeed, such typos were present and are now fixed.

Reviewer #2 (Remarks to the Author):

I thank the authors for engaging in constructive discussion and for their detailed answers to all of my points.

I am satisfied with the description of previous literature, and with the explanation about the comparison with Ref. 35, where now I see it is plausible that the experiments are compatible. I think it would be useful to write explicitly in the text that describes Fig 4 that Delta_1/2 = Delta_s \pm \Delta_f and the values (250 and 530) for the two coherence peaks.

Regarding alternative explanations of the gap splitting, it is also true that if a momentum dependent gap were the explanation, then some signature of this would presumably be seen in the bulk too, although the dependence of gap anisotropy in number of layers has never really been calculated. Nevertheless, the authors have explained their point of view clearly and compared with alternative explanations so I think this is sufficient.

I also understand better the logic of the theoretical model. The authors are not trying to make a model for the misfit compound, but rather to show the generic point about how interlayer coupling washes out the singlet-triplet splitting in centrosymmetric structures. I also see now the symmetry discussion was inspired by Ref. 78 which incorrectly assigned D3d symmetry to SrPtAs, an error that appears to have propagated through the literature but was later fixed in Fischer, JPSJ 84, 054705 (2015). The space group of SrPtAs is 194 (D6h) and the analysis is therefore the same as in TMDs. Incidentally, the f-wave function (with opposite sign in K and K', i.e. odd under the mirror sigma_v, but even under the mirror sigma_h) is not A2u but B2u in D6h. The text claims the function with A2u symmetry is even under the mirror line Gamma-M, but the function drawn on the lower part of Fig. 5a is odd (as it should to have opposite sign on K and K').

To be more precise, the pairing function in Ref. 78 has three pieces: the momentum form factor, the layer matrix, and the spin factor. For the centrosymmetric f-wave triplet, the momentum form factor is B2u, the z-spin triplet is A2g, and the pairing is opposite in the two layers (a B1u function). The product of these three gives A1g and is therefore allowed to mix in with the standard s-wave. In simple terms, the f-wave triplet pairing is allowed in centrosymmetric bilayers if it takes opposite signs in the two layers. This triplet piece is disfavored by interlayer coupling, which is the point of the calculation.

In summary, I only think the symmetry analysis needs a more careful look but I otherwise I am happy to recommend publication in NC.

We are grateful to the referee for their positive and encouraging assessment, and for considering our manuscript suitable for publication in *Nature Communications*. Following the referee's suggestion we added the next sentence in the end of the fourth paragraph of the discussion to enhance the clarity of our fit results "This scenario agrees well with values obtained from the fit, yielding $\Delta_{1,2}$= *s* ± *f*, Δ_1=530 μeV and Δ_2=250 μeV corresponding to the coherence peaks and slope change energies."

Regarding the comment about the theoretical model and symmetry, we greatly thank the referee for pointing out another mistake in the symmetry labeling and in the text (the ΓM reflection comment). We use the convention where it is the B_{1u} that reduces together with A_{1g} to become A' in D_{3h} upon breaking inversion and reducing the rotational symmetry from 6-fold to 3-fold (rather than B_{2u}). This is because B_{1u} is the irrep that is even under $3\sigma_v$, which we identify with the ΓK mirrors, while odd under $3\sigma_d$, which are the three ΓM mirrors. We have now corrected this in the text and figures and hope this is agreeable.

Reviewer #3 (Remarks to the Author):

The authors have made great efforts to revise the manuscript and address the comments raised in the previous round of review. However, after carefully considering both the revised manuscript and the authors' responses to the comments, several issues still remain insufficiently addressed. These points should be further clarified before the manuscript can be considered for publication in Nature Communications.

1. The authors clarified that the double-gap superconducting features observed in the transport measurements arise from superconductivity in the SnS and TaS2 layers. Under this interpretation, the results shown in Fig. 2 appear to be only weakly related to the intrinsic double superconducting gaps in the TaS2 layer that the manuscript focuses on and thus do not seem to provide strong support for the main conclusions of the work.

We thank the referee for this important comment and for the opportunity to clarify this point.

We agree that the `two-gap features' in transport data do not directly originate from the multi-gap structure of the order parameter within the 1H-TaS_2 layer. Rather, the transport data reflect contributions from both the SnS and TaS2 layers. Disentangling the individual gap contributions to Hc2(T) from transport measurements is beyond the scope of the present work.

That said, we believe it is fair to state that the transport results are consistent with, and supportive of, the phenomenology revealed by the STM measurements, even though transport alone cannot unambiguously establish the microscopic origin of the two-gap behavior.

More broadly, this work presents two distinct experimental findings in misfit layer compounds: (i) the existence of superconductivity in the SnS layer, which plays a significant role in transport and is supported by Fig. 3, Supplementary Figs. S8 and S9, and (ii) the unconventional superconducting order parameter in the 1H-TaS2 layer, which is established through layer-resolved STM measurements. The central conclusion of the manuscript relies on the latter, while the transport data provide complementary information on the full heterostructure.

To avoid any possible confusion regarding the role of the transport data, we have revised the manuscript accordingly. Specifically, we have reorganized Fig. 2 by moving the

resistivity data to the Supplementary Information, while retaining panel (b) as part of a revised Fig. 1 to illustrate the two-gap behavior and monolayer stacking. In addition, two new Supplementary Figures have been included.

2. In their response, the authors comment that monolayer TaS_2 grown on HOPG may be affected by strain and defects. Similarly, there is also a lattice constant mismatch between SnS and TaS_2. As shown in Fig. 1, this mismatch leads to the formation of moiré patterns, and the large-area topography provided in the authors' response also suggests the presence of strain in the sample. In addition, as the authors noted, the SnS layer is subject to significant pressure. Under these circumstances, it would be important to clarify whether such strain and interlayer pressure could influence the superconducting state of the TaS_2 layer.

We thank the referees for raising this important issue. First, we emphasize that **our data was taken, to the best of our ability to make this statement, in strain free regions.** Topography scans show that the strain in the 1H layers appear to be relieved through the formation of the large-scale features, or wrinkles, Fig. 3 of this rebuttal letter. The data set shown in Fig. 3 of the revised manuscript was acquired at the location marked by the white square in the large-scale topography shown here. The location, where the data shown in Fig. 3 of the revised manuscript was acquired from, is away from large-scale wrinkles, and the data of Supplementary Fig. S16 was acquired next to a step edge, where the topography lacks any visible strain.

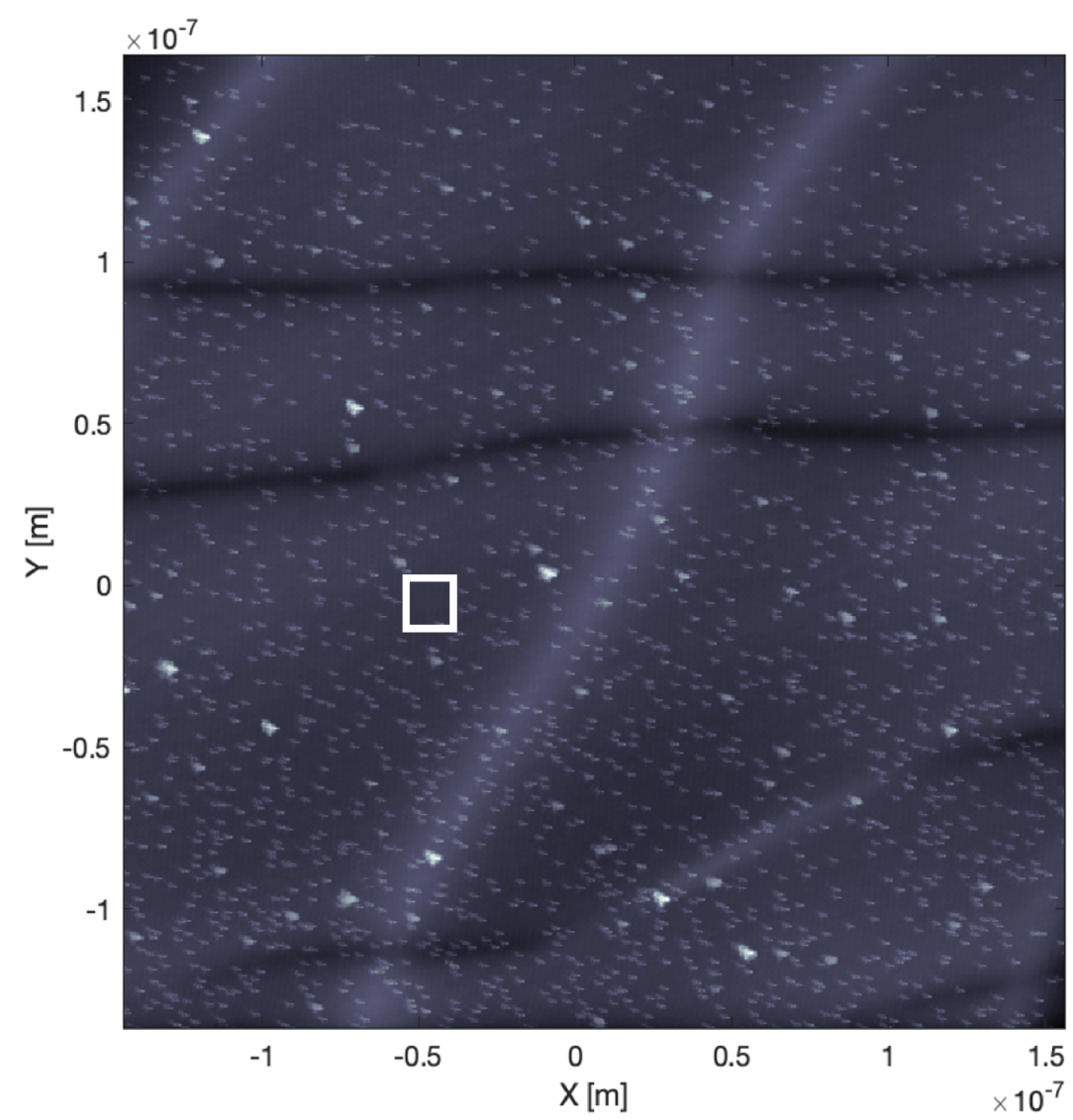

Figure 3 - Large scale topography showing the location of the line cut of Supplementary Figure 7, with respect to large scale topography lines. These lines represent locations where strain is relieved in the 1H layer.

In both data sets, Fig. 3 of the revised manuscript and Supplementary Fig. S16, the **spectra are uniform along a line cut of over 30 and 50 nm, respectively, thus supporting the idea that the spectra is inherent to the sample and not to strain,** which changes drastically with location as shown here on the right. We have added this figure as panel c to the revised Supplementary Fig. S7 showing that the data of Fig. 3 in the revised manuscript was taken away from strain. Also, we added the next clarification at the discussion:

“We emphasize that all data were acquired in strain-free regions. Any strain arising from the lattice mismatch between the SnS and 1H layers appear to be relieved through the formation of large-scale line features observed in the topography (Supplementary Fig. S7). The measurements were performed in regions away from such lines (Supplementary Fig. S7), as well as adjacent to a step edge where no visible strain is detected (Supplementary Fig. S16).
Furthermore, the data were collected along line cuts extending over 30 nm and 50 nm, such as the data sets in Fig. 3 and Supplementary Fig. S16 respectively. The high degree of spectral uniformity along these cuts supports the conclusion that the observed spectra are intrinsic to the sample and not induced by strain.”

As for monolayer 1H-TaS2 grown on HOPG, the strain created in the MBE growth process might produce substantial strain uniformly, and more profoundly due to the bulk substrate, as opposed to our crystal in which the strain seems to relieve itself in the form of large-scale lines of localized strain due to the vanishing inter layer coupling of the SnS and TaS2 layers. Furthermore, such strain can alter the mixing of the gaps, yet the mixing is a prerequisite thus its existence at very low temperatures is required $T << T_C$.

Furthermore, in the MBE grown sample the authors indicate that their spectra are different at different locations on the sample, which they attribute to disorder, **while in our case the spectra is uniform along a line of several tens of nanometers at different locations of the sample.** The effect of strain and defects in our discussion comes to explain the discrepancy seen in our 1H and the MBE grown 1H layers, where a mixing of s and f waves exists in our sample, while nodal and nodeless gaps are seen in separately at different locations in the MBE grown sample.

Also, it is true that different attributes are susceptible to strain such as T_C, gap size, normal state electronic structure etc. [Steppke et al. "Strong peak in T c of Sr2RuO4 under uniaxial pressure." *Science* 355, 6321 (2017); Zeljkovic "Strain engineering Dirac surface states in heteroepitaxial topological crystalline insulator thin films." *Nature nanotechnology* 10, 10 (2015)], shown also in the MBE grown sample where T_C is substantially suppressed, yet such scenarios are beyond the scope of the current study. Additional studies using strain will shed further light on the superconducting state shown to exist in this study.

Equally interesting, we note that s-wave order-parameter does not alter under applied strain as in the case of monolayer FeSe on SrTiO3 [Fan, Q., et al. "Plain s-wave superconductivity in single-layer FeSe on SrTiO 3 probed by scanning tunnelling microscopy." *Nature Phys* 11, 946−952 (2015)] where a single gap s-wave spectroscopy has been measured using STM although a single layer was grown with inherent strain on

bulk substrate. Thus, if the gap in monolayer 1H-TaS2 is plain s-wave, we do not expect a change in gap structure due to the strain involved.

Finally, we note that we cannot exclude the presence of a small amount of strain in the 1H layers. Using asymmetry of the Bragg-peaks locations in FFT of the topography we can determine a maximum asymmetry of ~0.5%, which is our measurement-error, therefore the conclusions drawn in this study can be attributed to strain free 1H layers, although a finite amount of strain is plausible. The actual amount of pressure is unknown to us, and the theoretical study of it is beyond the scope of this study.

3. We also note that the temperature used in the ARPES experiment is far above the superconducting transition temperature. Therefore, it remains somewhat unclear whether the SnS and TaS2 layers are still decoupled at temperatures near the superconducting transition (the authors mentioned charge transfer between the two layers in their response). In addition, in Fig. S2 the authors present calculations for two types of interlayer hopping. Could the authors clarify the physical justification for choosing the parameter t = 65 meV? It would also be helpful if the authors could provide results for a broader range of t values (e.g., 0-65 meV) to further strengthen the argument. Furthermore, the authors state that "The comparison demonstrates that the experimental data are consistent with a vanishing effective interlayer coupling between the two 1H-TaS2 layers in the misfit compound." In this case, what about the coupling between TaS2 and the adjacent SnS layer?

We thank the referees for this important comment.

ARPES temperature: The temperature used in the ARPES experiment is ~10K, and it is true that the phase transition occurs at a lower temperature of 3K, yet the energy scale associated with such a temperature difference is **much smaller than the energy scale associated with the inter-layer coupling**. A temperature difference of 7K can be translated to roughly **600 μeV**, while the energy scale of the band structure and the interlayer coupling, as measured before, using ARPES for **2H-TaS$_2$**, is **65 meV which is about 100 times bigger**. This makes it unreasonable for the two layers to be coupled from a band structure point of view as we enter the superconducting state. Moreover, **charge transfer mechanisms are due to work function mismatch** of the two constituent layers, and as such are taking place at the material synthesis temperatures. Thus, no change is expected as we cool down the sample from 10K and into the superconducting phase. Finally, the decoupling of the gaps is also evident in Supplementary Fig. S9 of the revised manuscript where the filling of the gaps due to applied magnetic field is different in each layer.

Value of t in calculation: Regarding the value used in the calculations, it is **based on previous ARPES** measurements as cited from ref. 42, where meticulous analysis of the band structure at the M point yields this value for the inter-layer coupling, and **transport and critical field measurements** given in ref. 34. As such, we use it as a **benchmark** for comparing our misfit layer to the pristine polymorph 2H-TaS_2 where the tunneling spectra provides a **single s-wave gap behavior**. We now explain better the origin of this value in the supplementary section where we added a comment about the value being given from previous ARPES and transport measurements.

Moreover, according to the known Hamiltonian of the band structure around the M point of 2H-TMDC's, **the gap at the M point is directly proportional to the interlayer coupling** [Almoalem et al. "Charge transfer and spin-valley locking in 4Hb-TaS2." *npj Quantum Materials* 9.1 (2024); Xiao et al. "Coupled spin and valley physics in monolayers of MoS 2 and other group-VI dichalcogenides." *Physical review letters* 108, 19 (2012)]. Thus, we did not calculate the M-L separation of the bands for additional values of the inter-layer coupling.

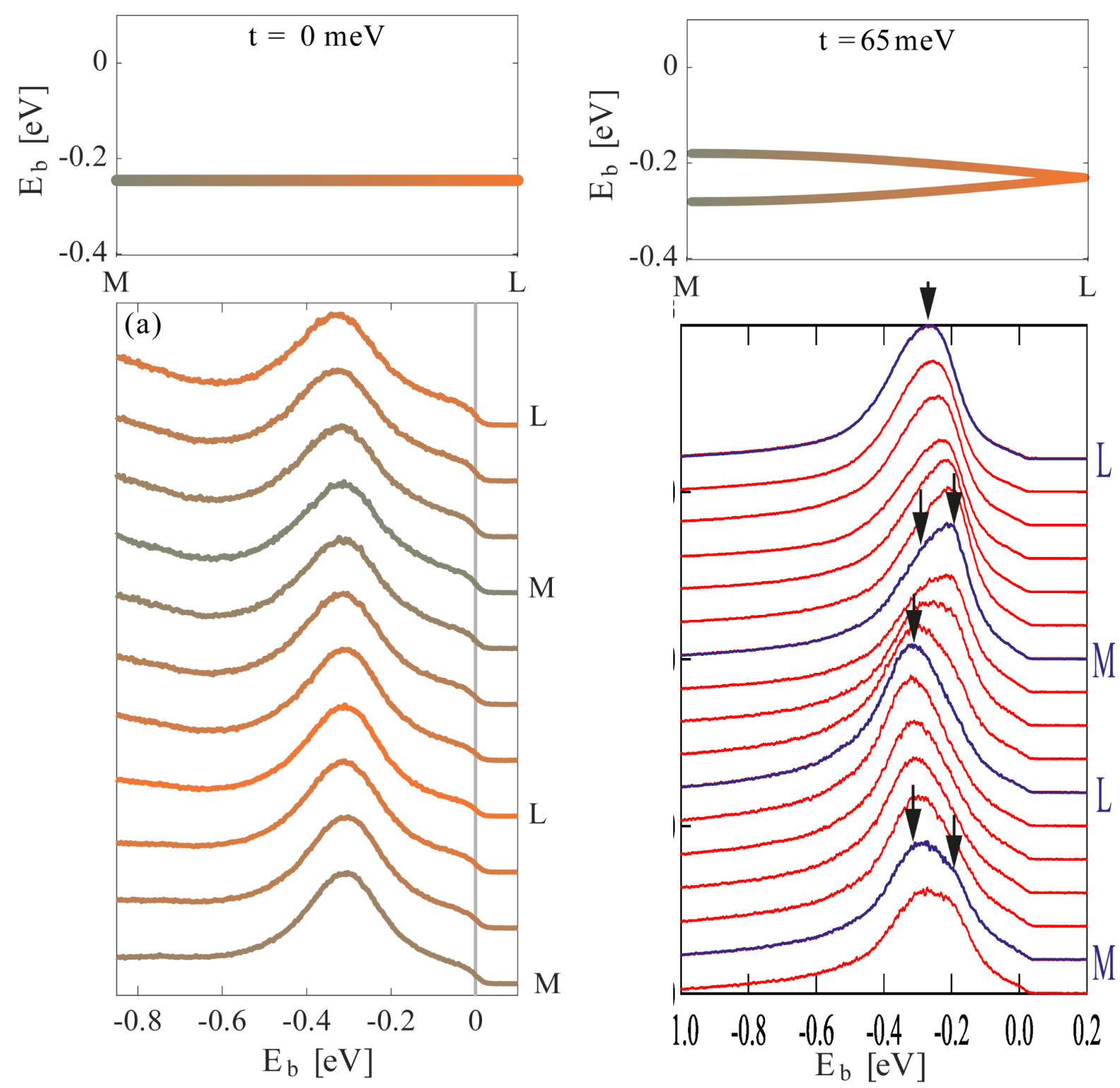

Figure 4 – **Top left**: Calculated band dispersion along the M-L line for an inter-layer hopping of t=0~meV, showing the absence of any kz dependent gap. **Bottom left**: Energy distribution curves (EDCs) taken along the M-L direction of the 1H-TaS2 Brillouin zone. The different kz values are accessed by varying the photon energy from 49 to 59eV, covering approximately one and a half Brillouin zones along kz. The gray line marks the Fermi level. **Top right:** Same calculation as in a for t=65mev, showing a kz dependent bonding antibonding splitting. The comparison demonstrates that the experimental data are consistent with a vanishing effective inter layer coupling between two consecutive 1H layers in the misfit compound. **Bottom right:** EDCs taken along the M-L direction of the 2H-TaS2 Brillouin zone. Adopted from ref 42.

Nevertheless, we show here a direct comparison between the **pristine polymorph 2H-TaS_2 (data taken from ref. 42) and our data for SnS-TaS2, Fig. 4 of this rebuttal letter**. The direct comparison shows the difference between the **dispersion along k_z** and the lack of it, which justify the notion of **vanishing inter-layer coupling**.

We have revised Supplementary Fig. S4 according to the figure shown here and added a new Supplementary Fig. S5 of a **simulation**, using real experiment parameters taken from fitting the data as in ref. 42, **of the expected energy distribution curves** at the M and L point of the Brillouin zone in the case of an **interlayer coupling of t=15 meV**. **The simulation shows a clear difference between the EDCs**, thus reinforcing our notion that the inter-layer coupling is negligibly small and constrained to be below 10 meV.

We have also revised the results section to include the next part, to further clarify the vanishing of the inter-layer coupling:

"The vanishing inter-layer coupling is further shown by comparing the EDC at the L point, where the gap is closed from symmetry considerations [41,42], and the M point where the gap is the largest for a given coupling. The two EDC are identical, as opposed to pristine 2H-TaS2, thus reinforcing the conclusion that the inter-layer coupling is smaller than our ARPES resolution of 10 meV, Supplementary Fig. S4 and S5."

Vanishing inter-layer coupling: The vanishing of the inter-layer coupling and the statement regarding the charge transfer are visible also by directly comparing the **ARPES data along the K-M-K' line** taken on the pristine polymorph **2H-TaS$_2$**, the heterostructure **4Hb-TaS$_2$** and the misfit compound **SnS-TaS$_2$**. Published data adopted from ref. 42 is used to directly show the vanishing inter-layer coupling similar to the 4Hb case and opposite to the 2H case, and equally important is the larger bandwidth which points to a significant charge transfer mechanism, Fig. 5 of this rebuttal letter.

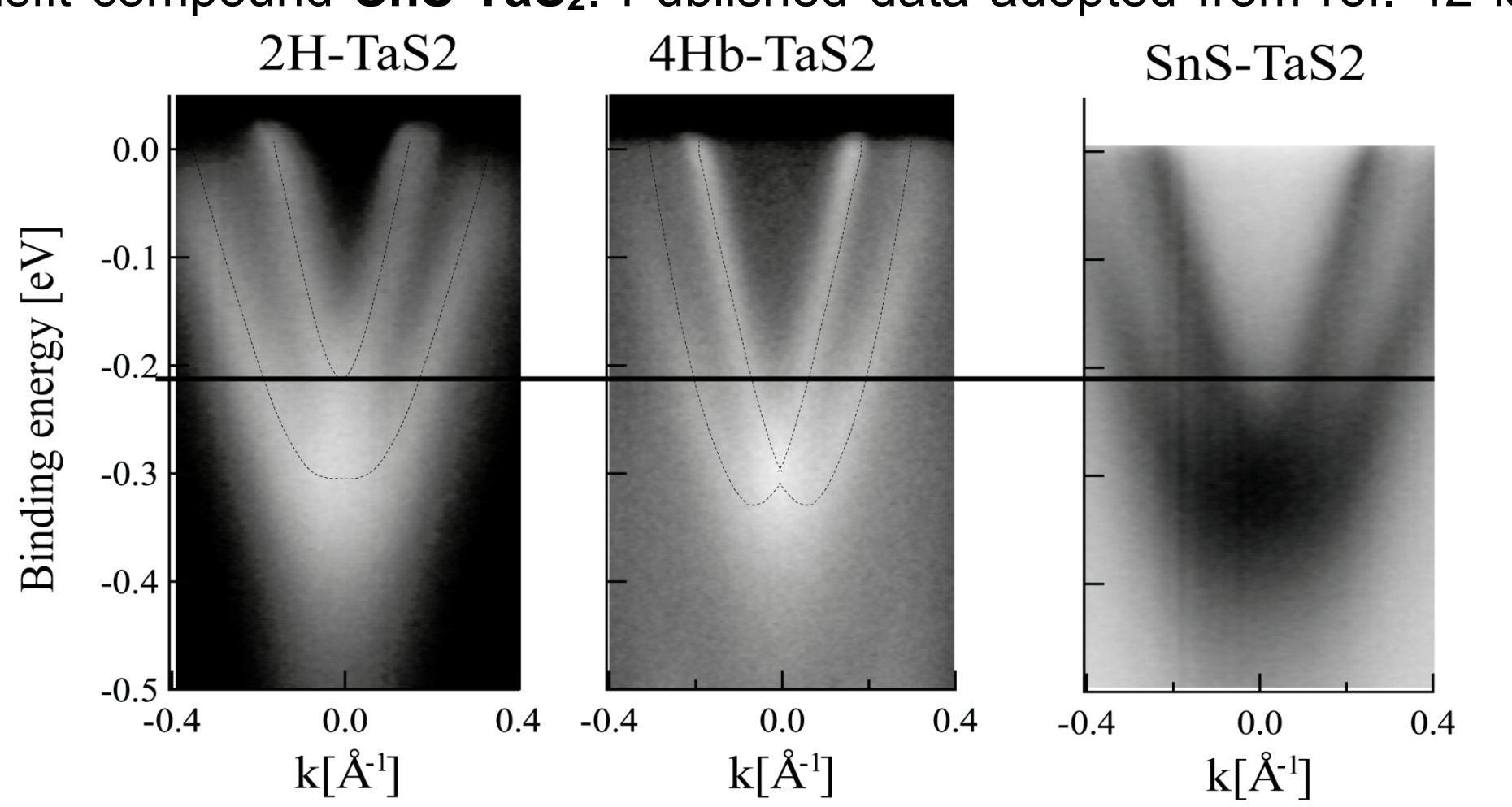

Figure 5 – **Left to right**: ARPES spectra taken along the K-M-K' line in 2H-TaS2, 4Hb-TaS2 and SnS-TaS2 misfit compounds respectively. Horizontal line marks the bottom of the upper band in 2H-TaS2. The bottoms of the band in 4Hb-TaS2 and SnS-TaS2 is vividly seen to be in much deeper than in 2H-TaS2, and the spectra in 4Hb-TaS2 and SnS-TaS2 are similar pointing on similar band structure in both polymorphs.

Moreover, **the sharpness of the bands in ARPES data is another indication of the k_z dispersionless bands** [Almoalem et al. "Charge transfer and spin-valley locking in 4Hb-TaS2." *npj Quantum Materials* 9.1 (2024)] *npj Quantum Materials* 9.1 (2024)].

We emphasize that the conclusion of vanishing interlayer coupling is due to the abundance of independent evidence such as: dispersionless spectra, spin-valley locking, lack of gap in the M point and the sharpness of the bands as opposed to the 2H polymorph.

Robustness of the s and f-wave mixing: Furthermore, as Fig. 4 of the revised manuscript shows, a small interlayer coupling on the order of 0.1*λ~12 meV (using the spin orbit coupling value obtained previously in ref. 42 and ref. 34) will result in a substantial admixture of the triplet and singlet components. Therefore, even an interlayer coupling on the scale of 10 meV results in an admixture of a singlet and a triplet order parameter, thus justifying our model. This is due to the lack of inversion symmetry of the unit cell and not only the 1H layers. The **non-centrosymmetric** structure of **SnS-TaS2** results in a more robust mixing between f- and s-wave, also in a scenario of a small finite inter-layer coupling. This enhances the visibility of the mixed gap function.

We added the next comment to the discussion to clarify this:

"We also note that the resolution of our ARPES measurements constrains the inter-layer coupling to be below 10 meV (Supplementary Figs. S4 and S5). Given a spin-orbit coupling of approximately 120 meV [34, 42], our theoretical analysis indicates that even an inter-layer coupling as large as t=10 meV leads to substantial mixing of singlet and triplet components, owing to the absence of inversion symmetry in the crystal."

1H and SnS layers coupling: We thank the referee for raising the issue of the SnS and 1H-TaS2 inter layer coupling. The interlayer coupling between the SnS and 1H-TaS2 layers, if present, will cause a modification of the band structure around the M point of the 1H-layer through a direct hybridization of the bands. Moreover, a second order hopping from the 1H to a consecutive 1H layer through the SnS layer, taking into account the symmetry of the material in c-axis, will also cause a modification of the band structure in the M point. Lack of modification of the band structure from pristine 1H structure, as seen in our data and in the Fermi-surface shown in the previous rebuttal letter, suggests that any effective inter-layer coupling, if exists, is smaller than our resolution.

4. Considering that the double superconducting gap signal in Fig. 4 appears relatively weak, how do the authors further rule out possible effects from tip states? In addition, the theoretical model presented by the authors seems rather idealized. Are there other theoretical works that support this scenario? Given that completely interlayer-decoupled TaS2 is effectively equivalent to a freestanding monolayer TaS2, are there previous theoretical studies that have reached similar conclusions?

We thank the referee for this important question.

First, while it is true that the change in the slope (kink) of the DOS is subtle, it is clearly seen in the second derivative of the current as shown in Fig.3c.

Moreover, we can rule out tip density of states effects, when we suppress the gap with a magnetic field (Supplementary Figure S9b) the kinks vanish when the **superconducting gap disappears, indicating that the features arise from superconductivity**. To emphasize this point, we show in Fig. 6 of this rebuttal letter, a second derivative (d^2I/dV^2) of the spectra taken above Hc2. The results are shown on the same scale to emphasize the lack of features. In the case of tip induced kinks, the same kinks would be expected to exist in this data as well. On a final note, the feature in the spectra shown in Fig. 3a,c of the revised manuscript is particle-hole symmetric which confirms its identification with the superconducting state.

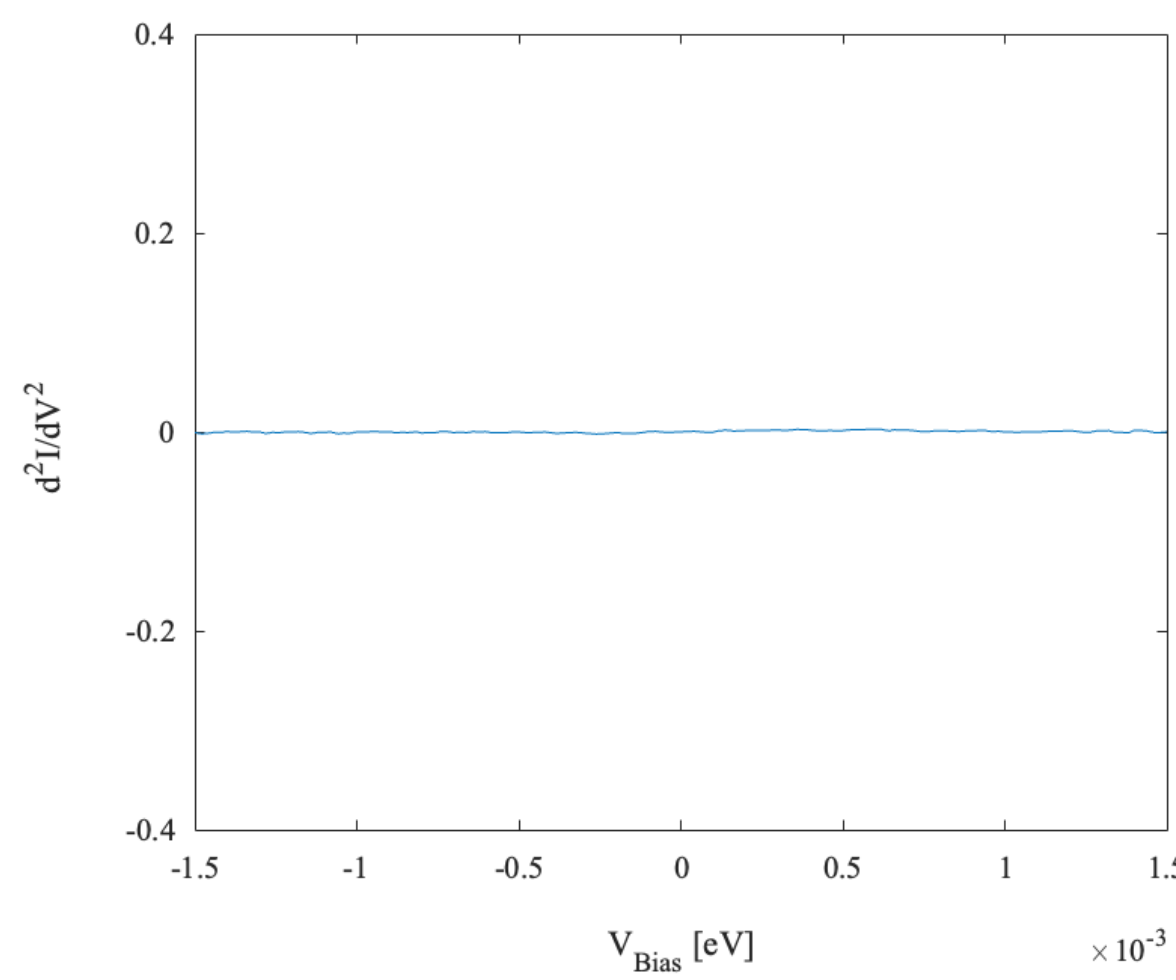

Figure 6 - $\frac{d^2I}{dV^2}$ taken from the data in Supplementary Figure S9b at 3kG. The scale is similar to the scale in Fig. 3c of the revised manuscript, and lack of any kinks in the spectrum taken above Hc2 naturally rules out a mundane explanation of tip induced features. This emphasizes the identification of the two-gap structure with the superconducting phase.

Regarding the theoretical model, the concept of singlet-triplet mixing in systems with D_{3h} symmetry (or in mono-layer H structured TMDs) is not new. For example, it was discussed explicitly in Refs. 78 and 85 of our paper. Furthermore, experimental evidence is shown in transport measurements of a few layers of NbSe2 revealing **a two-fold symmetric order-parameter where it is stated that "...attribute the behavior to the mixing between two closely competing pairing instabilities…"** (ref. 31), **collective Leggett-modes which influence the gap** in mono layer NbSe2 where it was stated that **"collective Leggett mode that represents the**

fluctuation toward a proximate *f*-wave triplet state" (ref. 89), and transport measurements in monolayer TaS2 where it was specifically stated that **"...reinforcing the conclusion that the appropriate theoretical treatment must incorporate effects beyond singlet pairing.."** (ref 34)**,** thus demonstrating that the ideal-case scenario is sufficiently robust in similar systems. Our only claim is that the strength of interlayer coupling in the 2H structure controls the degree of mixing and thus explains why it manifests in some cases and not others.

REVIEWER COMMENTS

We thank referees #1 and #2 for finding the revised manuscript suitable for publication in *Nature Communications*. We acknowledge Referee #3 concern regarding our interpretation of the differences between our system and the 4Hb-TaS_2 and the role of strain for the difference with MBE grown monolayer 1H-TaS_2. We therefore give our response for the last remarks given by the referees.

Reviewer #1 (Remarks to the Author):

I thank the authors for their detailed point-by-point response and for the further revisions to the manuscript and Supplementary material. Several of my concerns have been addressed. I would like to ask the authors to consider bringing the justification for using the misfit layer for their study in a more explicit way. Although the advantages of using the misfit layer could be found in a couple of sentences in the main text, it would be better to bring this point to the front as it is one of the key ideas of the manuscript, basically putting the scattered arguments into one or two places in the introduction part. Minor notes: In the caption for Fig. 3, "Fig. 1c." and "1e" may be "1d" and "1f", respectively. In addition, Refs. [77] and [84] are the same paper.

We are grateful to the referee for their thoughtful and positive assessment and finding our last version suitable for *Nature Communications.* Following the referee's suggestion, we added a dedicated paragraph to the introduction emphasizing the importance and added value of studying misfit-layer compounds, specifically crystals lacking inversion symmetry. The new introduction now reads:

"To answer this question and differentiate between an isolated monolayer and a system involving strongly correlated layers, we performed a low-temperature study using bulk and local probes of the superconducting phase in the misfit material $(SnS)_{1.15}(TaS_2)$. Misfit compounds, such as $(SnS)_{1.15}$ (TaS_2), $(SnSe)_{1.16}$ $(NbSe_2)$ and $(PbS)_{1.13}$ (TaS_2), are heterostructures of TMDC's and semi-conducting mono-chalcogen layers, with a miss-match between the lattice vectors of the different layers. This miss-match effectively decouples the layers into true two-dimensional systems [55].

In our specific case, the decoupling of the 1H layers not only create an electronic structure with reduced dimensionality but also provides us with a non-centrosymmetric superconductor. The lack of global inversion symmetry permits the mixing of gaps, otherwise distinct by symmetry, giving rise to a superconducting-gap more complicated than the mundane singlet s-wave, with a complex spin configuration. This new paved way of achieving

superconductivity based on pairing-channels with higher angular momentum therefore differentiates the system from other centrosymmetric TMDC's superconductors such as 4Hb-TaS_2 or $NbSe_2$. Although misfit materials have been known for a long time, recent interest in superconductivity in reduced-dimensional systems has renewed attention to these compounds, establishing them as a versatile platform for exploring two-dimensional superconductivity, where lattice incommensurability and vanishing inter-layer coupling can fundamentally reshape electronic and superconducting properties [56, 57]. In such systems, superconductivity often emerges as an intrinsically two-dimensional state with enhanced anisotropy and Ising characteristics, strongly influenced by electronic correlation effects [55, 58–60]."

We also thank the referee for pointing out references to be fixed and indeed those are now corrected in the revised manuscript.

Reviewer #2 (Remarks to the Author):

I thank again the authors for engaging in the discussion, and with the latest changes I support publication in NC.

We are grateful to the referee for their positive assessment of our manuscript. We appreciate their recognition of the significance of our findings and their consideration of the manuscript as suitable for publication in *Nature Communications*

Reviewer #3 (Remarks to the Author):

The authors have made considerable efforts to revise the manuscript and address the comments raised in the previous round of review. Nevertheless, after carefully evaluating both the revised manuscript and the corresponding responses, I remain unconvinced by several key issues and therefore do not recommend this work for publication in Nature Communications.

1) The authors' responses to Reviewer 1's comments concerning the theoretical model, 4Hb-TaS2, and impurities remain insufficiently convincing.

We acknowledge the referee concern regarding the theoretical model and the comparison to 4Hb-TaS_2. We do however disagree with the claim that our discussion comparing 4Hb-TaS_2 is insufficient, and by so suggesting the two samples should be treated is a similar manner, as the two systems exhibit **profoundly different attributes**:

1. The crystal structure of 4Hb-TaS_2 **has global inversion symmetry**. Although it was shown before that the interlayer coupling is negligible, therefore suggesting the system can be treated as isolated 1H layers, the **hybridization of the bands between the 1H and 1T layers** suggests a small residual inter-layer coupling may still exist and therefore the global inversion symmetry of the overall unit cell might still play a crucial role as seen in our theoretical model [Ribak, A., et al. "Chiral superconductivity in the alternate stacking compound 4Hb-TaS2." *Science advances* 6.13 (2020), Almoalem, Avior, et al. "Charge transfer and spin-valley locking in 4Hb-TaS2." *npj Quantum Materials* 9.1 (2024), Chen, Lu, et al. "Bulk Superconductivity driven by Disorder-Induced Delocalization in 4Hb-Ta($S_{1-x}Se_x$)$_2$." *arXiv:2606.07509* (2026)]. The overall unit cell, and not only the individual layers, of the **misfit compound in interest lack such inversion symmetry** therefore posing a material with fundamentally different physics. This is seen vividly in our theoretical model where the **lack of global inversion symmetry gives rise to a robust f-wave gap** even in the presence of small inter layer coupling, contrary to an inversion symmetric structure with small inter layer coupling which lack the f-wave gap. Such theoretical analysis was proposed before also for $NbSe_2$ as cited by us in the manuscript.

2. The 1T layer which comprise the unit cell of 4Hb-TaS_2 is a strongly correlated material, widely believe to be a Mott insulator in the pristine case and known to host a strong CDW phase. The proximity between the 1T and 1H layers, together with the known hybridization of the d orbitals between the layers, give rise to a more complicated scenario than purely reducing the inter layer coupling between the 1H layers [Kumar Nayak, Abhay, et al. "First-order quantum phase transition in the hybrid metal–Mott insulator transition metal dichalcogenide 4Hb-TaS2." *Proceedings of the National*

Academy of Sciences 120.43 (2023), Chen, Lu, et al. "Bulk Superconductivity driven by Disorder-Induced Delocalization in 4Hb-Ta($S_{1-x}Se_x$)$_2$." *arXiv:2606.07509* (2026)]. On the other hand, the lack of measurable hybridization between the layers in the misfit compound, as seen in our ARPES data, and lack of proximity between the 1H layers and strongly correlated metal, as the case of 4Hb-TaS_2 [Barantani, Francesco, et al. "Resonant chiral dressing by amplitude fluctuations in a ferroaxial electronic crystal." *Nature Physics* (2026).], preserves the basic electronic structure of the 1H-TaS_2 and related physics.

3. The lack of superconducting condensate in the 1T layers, as opposed to the SnS layer in this sample, emphasizes the different physics at low temperatures between 4Hb-TaS_2 and the misfit compound. The lack of vortex imaging and of superconducting gap [Nayak, Abhay Kumar, et al. "Evidence of topological boundary modes with topological nodal-point superconductivity." *Nature physics* 17.12 (2021)], together with the band existing right above the Fermi-level [Kumar Nayak, Abhay, et al. "First-order quantum phase transition in the hybrid metal–Mott insulator transition metal dichalcogenide 4Hb-TaS2." *Proceedings of the National Academy of Sciences* 120.43 (2023)] in the 1T layer show that the physics in the 1T layer is fundamentally different than in the SnS layers. Moreover, the electronic structure of the SnS originates from an inert and doped semiconducting materials, while the 1T electronic phenomena originates from a dope Mott insulator. Such discrepancies will affect the ground state of the material.

4. Last, experimental differences in the superconducting phase is seen in STM spectra measured in the 1H layer in both cases [Nayak, Abhay Kumar, et al. "Evidence of topological boundary modes with topological nodal-point superconductivity." *Nature physics* 17.12 (2021)]. The data pose a different scenario in both cases supporting our theoretical data. The superconducting spectra, the lack of edge states seen before, the different Fermi level band structure, the lack of CDW signature in transport which points on the different roles of the SnS and 1T layers, and the broken time reversal symmetry seen in 4Hb-TaS_2 and the lack of which in the misfit compound all point on different superconducting phase originating from different normal state physics.

The points outlined above makes the misfit compound a distinct material from 4Hb-TaS_2 and therefore should be regard as a new platform to study superconductivity and the implications of broken symmetries and reduced dimensionality, rather than be tested within the prism of 4Hb-TaS_2.

Regarding impurities, we are not familiar with claiming impurities as playing a role in 4Hb-TaS_2. We did discussed **disorder** regarding the MBE grown 1H-TaS_2, rather than 4Hb-TaS_2. It was suggested by the authors of ref. 54 that disorder was responsible to the quenching of the s-wave gap and the formation of a pure f-wave gap, therefore it plays a

major role in the formation of the superconducting condensate, and the energy of the different gaps.

The description of the MBE grown layer as having a “low defect density” suggests that residual **defects** are still present, and according to the authors explanation, potentially affecting the fragile superconducting behavior of the 1H-TaS_2 layer. This is also evident by the very low Tc of their sample (Tc~1K) opposed to the known Tc of monolayer TaS2 of ~3K, which we point out to be similar to Tc of our sample.

All this suggest that **disorder** might has a role in shaping the superconducting phase. **We emphasize that individual impurities or disorder do not play a role in our study,** as seen through the presence of impurities on the 1H layer and the homogeneity of the superconducting spectrum measured at different point and on the edge (supplementary figure S16), which is a strong scatterer.

2) The discussion concerning strain in the samples, as well as the interpretation related to monolayer 1H-TaS2 grown on HOPG, still lacks sufficient evidence and persuasiveness.

We thank the referee for giving us the opportunity to clarify this point. The discussion regarding the HOPG based sample rely on several key experimental findings:

1. **Tc:** The 1H layer grown on HOPG critical temperature is low (Tc~1K), which stand in contrast to the known Tc of monolayer TaS2 (Tc~3K). In our study, the critical temperature is the sample is shown to be as known from other studies. This suggest that the 1H layers in our study behave as expected from a free standing 1H monolayer.

2. **Homogeneity**: The 1H layer grown on HOPG was shown to have substantial differences in LDOS spectra measured at different locations. This points on another mechanism in play which is not consider such as disorder, charge transfer CDW nucleation etc. In our study the spectra were shown to be homogenous and to exist in various places, including on the edge of a 1H layer. As a strong scatterer, which mimics strong disorder, the edge should have create strong perturbation and cause a strong effect **if the origin of our spectrum was from such mechanisms (as discussed in the manuscript).**

3. **Theoretical explanation:** It was suggested by the authors of ref. 54 that disorder was responsible for quenching of the s-wave gap and the formation of a pure f-wave gap. Such an explanation stands in contradiction with our experimental data, where the formation of **pure f-wave** is absent.

As such we find our paper to important as it shows an example for a system with experimental finding that contradicts the theory presented in ref. 54. The most natural difference between the two platforms is the strain applied on the 1H layer, though this

might not be the sole cause for the discrepancy. The lattice mismatch in MBE grown sample is present everywhere and might be substantial, affecting the ion-ion separation and the formation of the CDW phase. In our study, the 1H layers might also be subjected to some amount of strain, yet the experimental data suggest this effect is negligible. This is seen in large scale topography of the 1H layers, where most of the layer is flat with what seems to be lines where strain is relieved (see Discussion of manuscript and supplementary data), and the homogeneity of the spectrum at different locations. Therefore, strain was suggested as a natural explanation for such differences, yet we point out that a systematic study of the effect of strain is beyond the scope of this study. All of this is discussed and presented in the manuscript, in a more concise manner as this paper is not a direct comparison to ref 54.

"This behavior can be placed in context with other TMDC systems. In bulk 2H-TaS_2, STM measurements report a single gap of about 0.28 meV [35, 82]. In MBE-grown monolayer 1H-TaS_2, a single V-shaped gap has been reported [54] suggesting a nodal gap structure, although with a low T_c around 1 K. Lower T_C suggests that other mechanisms might affect the observed superconducting characteristics such as disorder. Contrary to the MBE grown monolayer we do not observe the formation of a single component nodal order-parameter, exclusively in ultra-clean samples, rather a two-gap structure uniformly at all locations. This may point to the influence that the substrate has on the sample quality and the observed signatures. As an example, strain from the HOPG substrate, due to mismatch of the lattice parameters, might influence the electron-phonon coupling or the phonons themselves to create different order parameters [83]. Moreover, different interactions (on- site, nearest-neighbor etc.), due to different ion distances, can lead to different admixtures of the order parameters. In contrast, exfoliated monolayers with T_c near 3.5 K display two distinct gaps of approximately 0.25 and 0.46 meV [35], in close agreement with our findings. We emphasize that all data were acquired in strain-free regions. Any strain, arising from the lattice mismatch between the SnS and 1H layers, appears to be relieved through the formation of large-scale line features observed in the topography (Supplementary Fig. S7). The measurements were performed in regions away from such lines (Supplementary Fig. S7), as well as adjacent to a step edge where no visible strain is detected (Supplementary Fig. S16)."

The manuscript investigates unconventional superconductivity in the misfit compound $(SnS)_{1.15}(TaS_2)$, composed of alternating SnS and 1H-TaS_2 layers. By combining transport, ARPES, and STM spectroscopy, the authors suggest that the SnS layers electronically decouple the TaS_2 sheets, leading to quasi-two-dimensional superconductivity with a clear two-gap feature. A theoretical model incorporating broken inversion symmetry and finite-range pairing interactions reproduces the observed mixed singlet-triplet character. Overall, the work provides new insights into how unconventional pairing emerges in isolated 1H-TaS_2 layers and highlights misfit compounds as a promising platform for exploring multi-gap and parity-mixed superconductivity. However, several aspects of the study remain insufficiently addressed, which currently limits the clarity and rigor of the manuscript. I am unable to recommend the manuscript for publication in *Nature Communications* at this stage. However, it may become suitable once the authors have carefully addressed the points detailed below.

(1) Figures 2 and 3 present the transport and ARPES measurements. However, since these are non-local techniques and the SnS layer is also reported to be superconducting, it is crucial to clarify the role of the SnS layer. The discussion of this aspect is currently insufficient and somewhat unclear. For instance, the authors state in the main text (lines 159-163) that the coherence length extracted from the vortex agrees with that estimated from the upper critical field. Yet, in Supplementary Material V, the vortex is shown in the SnS layer, and no data are provided for the vortex in the 1H-TaS_2 layer. This inconsistency is confusing and undermines the logical coherence of the related discussion. Considering that a two-gap model was used in Fig. 2b to fit the evolution of the upper critical field, it appears that the authors attribute the transport and ARPES results solely to the TaS_2 layer, while the SnS layer is only included later in the self-consistent analysis (e.g., the discussion of the coherence length). I believe the authors need to provide clarification on these issues.

(2) The main text (lines 120-121) refers to Fig. 2c, but such a panel is not included in the currently presented Figure 2.

(3) In a previous work (Adv. Mater. 2023, 35, 2305409), nodal Superconductivity has been reported in the pristine monolayer 1H-TaS_2. I believe the authors should discuss the origin of this discrepancy about the gap features in the manuscript.

(4) As suggested by the theoretical model presented in the manuscript, the superconducting state without interlayer coupling appears weaker than that with interlayer coupling, which contrasts with the experimental findings. It would be helpful if the authors could further discuss this discrepancy.

(5) Given that multiple mechanisms can give rise to two-gap superconductivity, the

authors have not provided sufficient justification to exclude alternatives other than the mixed singlet-triplet pairing scenario. In Supplementary Material VI, ARPES data are presented to demonstrate a vanishing interlayer coupling between the 1H-TaS_2 layers; however, this evidence appears insufficient. Although the manuscript states that the superconductivity in SnS does not originate from the proximity effect of TaS_2, it remains important to further clarify whether any coupling exists between the superconducting states of the two layers, and how such coupling, if present, might influence the main conclusions.

The authors have made great efforts to revise the manuscript and address the comments raised in the previous round of review. However, after carefully considering both the revised manuscript and the authors' responses to the comments, several issues still remain insufficiently addressed. These points should be further clarified before the manuscript can be considered for publication in Nature Communications.

1. The authors clarified that the double-gap superconducting features observed in the transport measurements arise from superconductivity in the SnS and TaS_2 layers. Under this interpretation, the results shown in Fig. 2 appear to be only weakly related to the intrinsic double superconducting gaps in the TaS_2 layer that the manuscript focuses on, and thus do not seem to provide strong support for the main conclusions of the work.

2. In their response, the authors comment that monolayer TaS_2 grown on HOPG may be affected by strain and defects. Similarly, there is also a lattice constant mismatch between SnS and TaS_2. As shown in Fig. 1, this mismatch leads to the formation of moiré patterns, and the large-area topography provided in the authors' response also suggests the presence of strain in the sample. In addition, as the authors noted, the SnS layer is subject to significant pressure. Under these circumstances, it would be important to clarify whether such strain and interlayer pressure could influence the superconducting state of the TaS_2 layer.

3. We also note that the temperature used in the ARPES experiment is far above the superconducting transition temperature. Therefore, it remains somewhat unclear whether the SnS and TaS_2 layers are still decoupled at temperatures near the superconducting transition (the authors mentioned charge transfer between the two layers in their response). In addition, in Fig. S2 the authors present calculations for two types of interlayer hopping. Could the authors clarify the physical justification for choosing the parameter t = 65 meV? It would also be helpful if the authors could provide results for a broader range of t values (e.g., 0-65 meV) to further strengthen the argument. Furthermore, the authors state that "The comparison demonstrates that the experimental data are consistent with a vanishing effective interlayer coupling between the two 1H-TaS_2 layers in the misfit compound." In this case, what about the coupling between TaS_2 and the adjacent SnS layer?

4. Considering that the double superconducting gap signal in Fig. 4 appears relatively weak, how do the authors further rule out possible effects from tip states? In addition, the theoretical model presented by the authors seems rather idealized. Are there other theoretical works that support this scenario? Given that completely interlayer-decoupled TaS_2 is effectively equivalent to a freestanding monolayer TaS_2, are there previous theoretical studies that have reached similar conclusions?

The authors have made considerable efforts to revise the manuscript and address the comments raised in the previous round of review. Nevertheless, after carefully evaluating both the revised manuscript and the corresponding responses, I remain unconvinced by several key issues and therefore do not recommend this work for publication in Nature Communications.

1) The authors' responses to Reviewer 1's comments concerning the theoretical model, 4Hb-TaS2, and impurities remain insufficiently convincing.

2) The discussion concerning strain in the samples, as well as the interpretation related to monolayer 1H-TaS2 grown on HOPG, still lacks sufficient evidence and persuasiveness.
